# Interventions for improving executive functions in children with foetal alcohol spectrum disorder (FASD): A systematic review

**DOI:** 10.1002/cl2.1258

**Published:** 2022-11-03

**Authors:** Joseph Lee Betts, Elizabeth Eggins, Ned Chandler‐Mather, Doug Shelton, Haydn Till, Paul Harnett, Sharon Dawe

**Affiliations:** ^1^ School of Applied Psychology Griffith University Brisbane Australia; ^2^ Child Development Service Gold Coast Hospital and Health Service Southport Australia; ^3^ Child Development Service, Gold Coast Hospital and Health Service Southport Australia; ^4^ School of Criminology and Criminal Justice Griffith University Mount Gravatt Australia

## Abstract

**Background:**

The consequences for children born with birth defects and developmental disabilities encompassed by foetal alcohol spectrum disorder (FASD) are profound, affecting all areas of social, behavioural and cognitive functioning. Given the strong evidence for a core deficit in executive functioning, underpinned by impaired self‐regulation skills, there has been a growing focus on the development of interventions that enhance or support the development of executive functions (EFs).

**Objectives:**

The primary objective of this review is to synthesise the evidence for structured psychological interventions that explicitly aim to improve EF in children. The review also sought to ascertain if the effectiveness of interventions were influenced by characteristics of the intervention, participants or type of EF targeted by the intervention.

**Search Methods:**

Sixteen databases, 18 grey literature search locations and 9 trial registries were systematically searched to locate eligible studies (up to December 2020). These searches were supplemented with reference harvesting, forward citation searching, hand searches of topic‐relevant journals and contact with experts.

**Selection Criteria:**

Studies were included in the review if they reported on an impact evaluation of a psychological intervention aiming to improve EF in children 3–16 years who either had confirmed prenatal alcohol exposure or a formal diagnosis falling under the umbrella term of FASDs. Eligible study designs included randomised controlled trials (RCTs) and quasi‐experimental designs with either no treatment, wait list control or an alternative treatment as a comparison condition. Single‐group pre‐post designs were also included.

**Data Collection and Analysis:**

Standard methodological procedures expected by the Campbell Collaboration were used at all stages of this review. Standardised mean differences (SMDs) were used to estimate intervention effects, which were combined with random effects meta‐analysis (data permitting). Risk of bias was assessed using the Cochrane Risk of Bias Tool (RoB2) and Cochrane Risk of Bias in Non‐Randomised Studies‐Interventions tool (ROBINS‐I).

**Main Results:**

The systematic search identified 3820 unique records. After title/abstract and full‐text screening, 11 eligible studies (reported in 21 eligible documents) were deemed eligible, with a combined 253 participants. Of the 11 studies, 6 were RCTs, 1 was a quasi‐experiment and 4 were single‐group pre‐post intervention designs. All studies were rated as having an overall high or serious risk of bias, with some variation across domains for RCTs. For RCT and quasi‐experimental studies, the overall effect of EF interventions on direct and indirect measures of EF generally favoured the experimental condition, but was not statistically significant. There was no difference between intervention and comparison groups on direct measures of auditory attention (*k* = 3; SMD = 0.06, 95% confidence interval [CI] = −1.06, 1.18), visual attention (*k* = 2; SMD = 0.90, 95% CI = −1.41, 3.21), cognitive flexibility (*k* = 2; SMD = 0.23, 95% CI = −0.40, 0.86), attentional inhibition (*k* = 2; SMD = 0.04, 95% CI = −0.58, 0.65), response inhibition (*k* = 3; SMD = 0.47, 95% CI = −0.04, 0.99), or verbal working memory (*k* = 1; *d* = 0.6827; 95% CI = −0.0196, 1.385). Significant heterogeneity was found across studies on measures of auditory attention and visual attention, but not for measures of cognitive flexibility, attentional inhibition or response inhibition. Available data prohibited further exploration of heterogeneity. There was no statistical difference between intervention and comparison groups on indirect measures of global executive functioning (*k* = 2; SMD = 0.21, 95% CI = −0.40, 0.82), behavioural regulation (*k* = 2; SMD = 0.18, 95% CI = −0.43, 0.79), or emotional control (*k* = 3; SMD = 0.01, 95% CI = −0.33, 0.36). Effect sizes were positive and not significant for meta‐cognition (*k* = 1; SMD = 0.23, 95% CI = −0.72, 1.19), shifting (*k* = 2; SMD = 0.04, 95% CI = −0.35, 0.43), initiation (*k* = 1; SMD = 0.04, 95% CI = −0.40, 0.49), monitoring (*k* = 1; SMD = 0.25, 95% CI = −0.20, 0.70) and organisation of materials (*k* = 1; SMD = 0.25, 95% CI = −0.19, 0.70). Effect sizes were negative and not statistically different for effortful control (*k* = 1; SMD = −0.53, 95% CI = −1.50, 0.45), inhibition (*k* = 2; SMD = −0.08, 95% CI = −0.47, 0.31), working memory (*k* = 1; SMD = 0.00, 95% CI = −0.45, 0.44), and planning and organisation (*k* = 1; SMD = −0.10, 95% CI = −0.55, 0.34). No statistically significant heterogeneity was found for any of the syntheses of indirect measures of EF. Based on pre‐post single‐group designs, there was evidence for small to medium sized improvements in EF based on direct measures (cognitive flexibility, verbal working memory and visual working memory) and indirect measures (behavioural regulation, shifting, inhibition and meta‐cognition). However, these results must be interpreted with caution due to high risk of bias.

**Authors' Conclusions:**

This review found limited and uncertain evidence for the effectiveness of interventions for improving executive functioning in children with FASD across 8 direct and 13 indirect measures of EF. The findings are limited by the small number of high‐quality studies that could be synthesised by meta‐analysis and the very small sample sizes for the included studies.

## 1 PLAIN LANGUAGE SUMMARY

### 1.1 Limited evidence on interventions to improve executive functions in children affected by prenatal alcohol exposure

This review found limited evidence for the effectiveness of interventions designed to improve executive function (EF) in children with prenatal alcohol exposure (PAE). However, the ability to detect overall effects is severely limited by a lack of large comparison studies in the area.

### 1.2 What is this review about?

Foetal alcohol spectrum disorder (FASD) is a preventable, life‐long disability which poses significant cost to individuals, families, and societies. Despite growing evidence exploring the profile of FASD, evidence on effective treatments of the condition is sparse.

Given prevalence estimates of around 5% in the general population, and higher in vulnerable samples, rigorous and comprehensive evaluation of feasible interventions for FASD are a necessity.

The focus of this review is studies which assess the impact of psychological interventions designed to improve EF in children impacted by PAE. Eligible outcomes include any measure of EF as per contemporary popular models of the construct (discussed in detail in methodology section of the systematic review). Examples include: attention, working memory, cognitive flexibility, inhibition, planning and organisation.

### 1.3 What is the aim of this review?

This Campbell systematic review examines the impact of interventions designed to improve executive functioning in children with a history of prenatal alcohol exposure. The review summarises evidence from eleven studies, including seven treatment‐comparison studies and four single‐group studies, with pre‐post outcome assessments.

### 1.4 What studies are included?

Eleven studies are included, with only seven randomised or high‐quality quasi‐ experimental studies, and four single group pre‐post intervention studies. The randomised and quasi‐experimental studies are synthesised using meta‐analysis. Of these seven studies, all were carried out in either Canada or the USA.

### 1.5 What are the main findings of this review?

Overall, the studies have important methodological weaknesses that temper the review findings, most notably very small sample sizes or incomplete reporting.

While the pattern of results arising from the synthesis generally suggest positive intervention effects, the analyses did not reach statistical significance. There appear to be no statistically significant differences between children with PAE who participated in EF interventions versus those who did not on the following direct measures of EF: auditory attention, visual attention, cognitive flexibility, attentional inhibition, response inhibition, verbal working memory, or planning.

Similarly, there appear to be no statistically significant differences between children with PAE who participated in EF interventions versus those who did not on the following indirect measures of EF: global executive function, behavioural regulation, emotional control, shifting or inhibition.

### 1.6 What do the findings of this review mean?

Only a small number of eligible comparison group studies are included in the present analyses (*n* = 7), and it is likely that relatively small sample sizes hinder detection of effects across outcomes.

The findings of this review therefore illustrate the need for a greater number of high quality comparison studies with larger sample sizes. This will allow for more definitive conclusions to be drawn regarding the overall effectiveness of interventions for EF in children with FASD.

### 1.7 How up‐to‐date is this review?

The review authors searched for studies up to December 2020.

## 2 BACKGROUND

### 2.1 Description of the condition

Prenatal alcohol exposure (PAE) is associated with profound and lifelong disability. The umbrella term foetal alcohol spectrum disorder (FASD) describes a spectrum of impairments resulting from the deleterious effects of PAE (Chudley et al., 2005). Historically, the spectrum comprised four disorders: foetal alcohol syndrome (FAS), partial FAS (pFAS), alcohol‐related neurodevelopmental disorder (ARND) and alcohol‐related birth defects (ARBD; Lange et al., 2017). All four disorders shared PAE as their aetiological base and all but ARBD were associated with neurological deficits (ARBD comprising physical defects). More recently, the Canadian and Australian guides for the diagnosis of FASD have been published (Bower et al., 2016; Cook et al., 2016). While the core aspects of the condition remain unchanged, the Australian and Canadian guides allow two diagnostic categories for FASD: (i) FASD with three sentinel facial features; or (ii) FASD with less than three sentinel facial features and an additional ‘At Risk’ classification if there is insufficient evidence or uncertainty about the appropriateness of the neurodevelopmental assessment.

While information on prevalence rates remains a significant challenge (Roozen et al., 2016), estimates have been as high as 5% in general population studies in the United States of America (USA) (May et al., 2014). Further, evidence suggests this rate may be higher in subpopulations such as First Nations people (Burd & Moffatt, 1994; Fitzpatrick et al., 2015; Popova et al., 2017) and children involved with the child protection system (Lange et al., 2013). Understanding how to support children with a diagnosis of FASD is particularly important given that the condition has been linked to poor outcomes across a range of developmental domains (Mattson et al., 2019). The condition is linked to a host of poor life outcomes, including increased contact with the justice system, substance misuse (Burd et al., 2010), antisocial and delinquent behaviour, learning disabilities, externalising and aggressive behaviour, as well as a range of other adaptive functioning and mental health problems (Bower, 2016; Kodituwakku Piyadasa, 2009; Rasmussen et al., 2008). Although there are no agreed upon usual treatments following a diagnosis of FASD, much of the clinical literature acknowledges the importance of ensuring families and caregivers have an understanding of how FASD can impact on children's behaviour and cognitions (Reid, 2015).

There is growing evidence that a core deficit underpinning many of these adverse outcomes is impairment in executive functions (EFs) (Khoury et al., 2015; Kodituwakku, 2009). EFs are higher‐order mental processes which allow individuals to deploy attention strategically, hold and manipulate goal‐relevant information, and consciously enforce goal‐directed behaviour (Baggetta & Alexander, 2016; Diamond, 2013). According to Diamond, EFs comprise three core abilities: (i) inhibitory control—the ability to inhibit prepotent responses of attention, behaviour, thoughts and/or emotions in favour of what is appropriate or necessary; (ii) working memory—the ability to hold information in mind and manipulate it in goal‐directed ways; and (iii) cognitive flexibility—the ability to solve problems using different perspectives or rules as they arise. Different combinations of these core EFs produce a range of higher‐order manifestations, including reasoning, problem‐solving, planning and directing attention (Diamond, 2013).

Children with FASD have frequently shown impairment compared to typically developing children across a wide range of both core EFs and higher order manifestations (Rasmussen, 2005). Deficits have been found on cognitive inhibition, verbal and nonverbal fluency (Schonfeld et al., 2001), use of attentional strategies, planning (Green et al., 2009), visual attention, spatial working memory (Rasmussen et al., 2011), behavioural inhibition, the ability to form complex concepts and cognitive flexibility (Rasmussen et al., 2013). Research also suggests that EF impairments are often more severe than would be suggested by IQ deficits alone (Connor et al., 2000).

Importantly, EF deficits may underlie many of the poor outcomes for individuals with FASD. For example, FASD is associated with a higher rate of attention deficit and hyperactivity difficulties (Rasmussen et al., 2010), and research has demonstrated that impaired EF may underpin ADHD. Impaired executive functioning has also been linked to a range of other outcomes found in FASD populations, including: autism (Geurts et al., 2004), obesity (Crescioni et al., 2011), lower quality of life (Davis et al., 2010), poorer school readiness (Shaul & Schwartz, 2014), impaired school performance (Visu‐Petra et al., 2011), financial problems, criminal behaviour and substance misuse (Moffitt et al., 2011). Consequently, addressing EF deficits in FASD populations may offer an important opportunity to improve both individual and societal outcomes.

Targeting children is particularly important as gains may be made with appropriate early intervention strategies. Indeed, there have been a number of studies evaluating the effectiveness of interventions aimed to improve EFs in children with FASD (e.g., Coles et al., 2015; Leung et al., 2015; Nash, 2012; Reid et al., 2017). However, a critical gap in the literature is the absence of an updated, comprehensive systematic review and meta‐analysis in the area. Given the compromised outcomes associated with EF deficits, and the frequency of EF impairment in children with FASD, a rigorous synthesis of the effectiveness of available interventions offers great value to practitioners, individuals with FASD and their families.

### 2.2 Description of the intervention

This review focuses on synthesising evaluation evidence for structured psychological interventions that explicitly aim to improve EF in children using either (i) a face‐to‐face format, (ii) computerised format, or (iii) both formats. in individual or group format, and/or where the intervention is administered either (i) directly to children (e.g., working memory training) or (ii) to children and caregivers/families (e.g., the GOFaR program).

### 2.3 How the intervention might work

#### 2.3.1 Child‐centred interventions

A common intervention of this type is specific or targeted EF training. Training interventions are the intentional teaching of skills through repetitive experience aimed at restoring or improving functions and are generally completed in the presence of a qualified practitioner. These interventions can be delivered face‐to‐face (Loomes et al., 2008) or as computerised training programs (Holmes et al., 2010). Tasks generally aim to improve skills by offering practice of specific abilities unique to EF domains (Holmes et al., 2010). These interventions often involve working through levels of increasing difficulty, allowing for continual optimisation of training impact (Klingberg et al., 2005). Child‐centred interventions can also be longer‐term programs whereby children work their way through a pre‐defined structure over a number of sessions (e.g., the Alert Program; Nash et al., 2015). These programs use similar mechanisms to short‐term training, relying on experiential activities to restore or improve functions.

#### 2.3.2 Parental involvement in EF training

In addition to focusing on the development of EFs in children, some programs provide instruction and support to parents and caregivers. This additional component can sit alongside the EF focus and be provided either concurrently in group or individual formats to parents and caregivers or as separate program components. For example, Coles et al. (2015) supplemented computerised training for children with FASD with a parental workshop on how PAE impacts neurodevelopment. Parents participated in group sessions that were run concurrently with their children's computer training. This aspect of intervention is often designed to improve parents' working knowledge of their child's neurodevelopmental functioning, facilitating the provision of more effective behavioural scaffolding from parent to child. Scaffolding of behaviours by parents and caregivers have been shown to support the generalisation of skills and provide children with an opportunity for support and repeated practice (Hammond et al., 2012).

### 2.4 Why it is important to do this review

#### 2.4.1 Previous relevant reviews and knowledge‐gaps

In recent years, increasing awareness of the prevalence of FASD across the world has sparked new interest in trialling interventions designed to ameliorate the deleterious effects of PAE on functioning. A number of recent systematic reviews have been published in the areas of FASD prevalence (Popova et al., 2017; Roozen et al., 2016), comorbidity (Popova et al., 2016) and to assess the impact of treatment (Peadon et al., 2009; Reid et al., 2015). The most recent review by Reid et al. (2015) provided a synthesis of the effectiveness of treatment interventions for FASD that included an assessment of methodological quality of FASD intervention studies across the lifespan. This narrative review included interventions targeting parenting skills, self‐regulation and attentional control, mathematics skills, nonverbal reasoning and social skills.

A limitation of Reid et al.'s (2015) review is the lack of a quantitative synthesis of effect sizes. By providing a meta‐analysis in parallel to the review, more precise conclusions can be provided regarding the overall effectiveness of interventions that address a core deficit in children with FASD. The current review will therefore provide both an updated review of interventions aimed at enhancing EFs, and a quantitative synthesis of these studies. A secondary objective of this review is to examine whether the effectiveness of interventions vary by a range of factors, including:

1. Variation in the number, setting, delivery and intensity of program components
2. Variation in program participants (e.g., gender, age, comorbid diagnoses, level of PAE)
3. Variation in the type of EF targeted by the intervention.

A systematic review protocol has also recently been published by Singal (2018) aiming to review effectiveness of interventions in FASD populations. The review intends to include meta‐analysis if possible, however aims to evaluate any outcome pertaining to children's physical and mental health as well as cognitive, behavioural and social skills. Whilst this review will provide a comprehensive and broad examination of the extant evaluation literature, a more focused review in a single domain affected by PAE will provide a concise synthesis of the existing evaluation studies and provide clear directions for policy and practice. By focusing on one domain of functioning (EF) and psychological interventions common in FASD practice and research, this review seeks to provide a more targeted synthesis of available literature. Thus, the current review will be the first to provide a comprehensive synthesis of the size of the effect of EF interventions for children with FASD.

#### 2.4.2 Practice and policy relevance

Globally, there have been a range of initiatives aimed at preventing FASD through increased public awareness of the effects of PAE. Concurrently, there has been development of clearer diagnostic processes (e.g., Astley & Clarren, 2000; Bower et al., 2016; Chudley et al., 2005). This review supports major initiatives and policy as outlined in key government documents. For example, the Australian Government's Commonwealth Action Plan seeks to ‘manage the impact of a diagnosis of FASD on the individual and the family to ensure the child and the family are supported through and after the diagnostic process’ (Australian Government, 2014, p. 3). Similarly, the Canadian Government's FASD Framework for Action sets out an objective of ‘meeting the needs of individuals with FASD, their families and communities’ (Public Health Agency of Canada, 2005, p. 9). This includes improving outcomes and helping individuals to reach their full developmental potential. In the United States, the National Task Force on Foetal Alcohol Syndrome (US Department of Health and Human Services, 2009) sets out the objectives of intensifying research initiatives around FASD and promoting comprehensive continuums of care for individuals with FASD.

More broadly, this review will provide evidence that will build the capacity of practitioners and policy‐makers to make informed choices regarding treatment and pathways of care for FASD children globally. It is also hoped that the results of this review will aid policy‐makers tasked with drafting formal public health policy in the area of the impact of alcohol use on citizen health and welfare. This will ultimately drive better outcomes and improve supports as set out in the various government policy agendas.

## 3 OBJECTIVES

This review aimed to answer two research questions. First, how effective are EF‐focused psychological interventions for improving EFs for children with FASD? Second, does the effectiveness of the interventions vary by the (i) characteristics of the intervention (setting, delivery, intensity, number of components, parent involvement); (ii) characteristics of the participants (e.g., gender, age, comorbid diagnoses, level of PAE); or (iii) type of EF targeted by the intervention? To answer these research questions, the review systematically gathers and synthesises published and unpublished impact evaluations of psychological interventions aimed at improving the executive functioning of children with FASD. Data permitting, the review  provides quantitative effect estimates for the overall treatment effect for EFs in children with FASD and whether it varies by the aforementioned factors.

## 4 METHODS

### 4.1 Criteria for considering studies for this review

#### 4.1.1 Types of studies

This review included randomised controlled trials (RCTs) where participants were randomly allocated to an experimental or comparison treatment condition, and also randomised cluster control trials where pre‐defined clusters of groups were randomised to the experimental or comparison condition. This review also included quasi‐experimental designs where participants were allocated to conditions in a manner other than randomisation (e.g., matched comparison group designs). In all of these designs, the experimental or treatment group refers to eligible participants who took part in the intervention designed to improve executive functioning, and the comparison group refers to those who were allocated to a wait‐list control or to receive, treatment‐as‐usual, no treatment or an alternative treatment.

Due to the burgeoning nature of this area of research, single‐group pre‐post designs were also included in the review. These designs are, however, subject to concerns regarding bias and are therefore analysed separately to RCTs and quasi‐experimental studies (Deeks et al., 2020).

#### 4.1.2 Types of participants

Eligible participants were children aged 3–16 years who had either (i) a formal diagnosis of FAS, FASD, pFAS, ARND or ‘at risk of FASD’ using any of the following diagnostic systems: The Institute of Medicine Diagnostic system (Hoyme, 2005), The Washington 4‐Digit Code (Astley & Clarren, 2000), The Canadian Guidelines (Chudley Albert et al., 2005) or the Australian Guide (Bower, 2016); or (ii) classified as having FAS based on facial dysmorphology alone; or (iii) confirmed or suspected PAE (light, moderate or heavy dosages). Children with a sole diagnosis of ARBD were excluded, as this condition is not associated with neurological deficits (Lange et al., 2017).

The minimum age range of 3 years was selected as it is possible to measure EFs at this age (Wiebe et al., 2011). Where the sample included some children that fell outside of the specified age range, the protocol (Betts et al., 2019) specified that study authors would be contacted to request data pertaining only to children within the age range. However, the review did not identify any studies with this issue. In future updates of this review, the procedure specified in the protocol will be followed should this issue occur.

There were no geographical restrictions on study location, however, variability among different countries/cultures that can impact diagnoses were anticipated. As such, the review used clear, formal diagnostic criteria to guide study inclusion. The protocol (Betts et al., 2019) specified that studies that used one of the four formal diagnostic frameworks mentioned above would be synthesised separately to studies where participants were included based solely on confirmed PAE or facial dysmorphology. The review does not separate analyses in this way due to the small number of included studies and the likelihood that the results would be piecemeal and less meaningful. In future updates of this review, the procedure specified in the protocol will be followed should there be sufficient studies to support this approach.

#### 4.1.3 Types of interventions

Studies were included if the focus of the intervention was to improve EFs in children with FASD. Interventions need to be structured psychological interventions that explicitly aimed to improve EFs in children using either (i) a face‐to‐face format, (ii) computerised format, or (iii) both. The review included interventions delivered in individual or group format, and/or where the intervention was administered either (i) directly to children (e.g., working memory training) or (ii) to children and caregivers/families (e.g., the GOFaR program). Studies where participants received both psychological interventions and pharmacological interventions concurrently as part of a focal treatment group were excluded.

The protocol for the review (Betts et al., 2019) specified that interventions needed to take place in a health clinic (e.g., medical, psychological clinic), school setting or in a home‐therapy setting. This inclusion threshold was relaxed for the review as some studies did not report the intervention setting but met all other inclusion criteria for the review.

#### 4.1.4 Types of outcome measures

##### 4.1.4.1 Primary outcomes

Studies were included in the review if they reported a measure of EF as defined by Diamond (2013) and Miyake et al. (2000); a set of higher order cognitive functions which combine to create abilities such as planning, organisation, attentional control and emotional regulation. The definition and categorisation of EFs, and the measures assessing them, tend to vary across the literature. For example, inhibitory control, working memory and cognitive flexibility are considered core EFs. Yet some research does not label outcomes with this exact terminology. There also tends to be variation in the extant literature regarding whether processes are EF or manifestations of EFs (e.g., reasoning, self‐regulation, behavioural regulation, problem‐solving, planning and attention). Therefore, this review took a comprehensive approach by including studies where the authors explicitly referred to the outcomes as EFs, explicitly measured core EFs (inhibitory control, working memory, cognitive flexibility) or included measures that some researchers consider to be manifestations of EFs (e.g., reasoning, self‐regulation, problem‐solving, planning and attention). Where it was unclear if the outcomes reported in a study were EFs, the protocol (Betts et al., 2019) specified that study authors would be contacted to verify the eligibility of the outcomes. This was not required for any studies captured by the review.

Studies were included if the outcome data was gathered using either standardised direct assessment of EF or through indirect parent/teacher reports of EF. Common standardised measures of EF in children include the following (not exhaustive):

1. Tests of variables of attention (T.O.V.A), a direct neuropsychological assessment that measures attention while screening for ADHD (Leark et al., 1996).
2. NIH toolbox, a direct assessment battery which provides measures of a range of cognitive abilities and EF in children aged 3 years+ (Gershon et al., 2013).
3. NEPSY‐II (attention and EF domains), which is a direct standardised assessment consisting of 32 sub‐tests for use in neuropsychological assessment with pre‐schoolers, children and adolescents (Brooks et al., 2009).
4. Behaviour Rating Inventory of Executive Function (BRIEF‐P/BRIEF‐2), which is a standardised indirect psychological assessment tool that measures EFs in pre‐schoolers (Gioia et al., 2003) and children (Gioia et al., 2003, 2016).
5. Child Behavioural Checklist (CBCL; attention and ADHD sub‐scales), which is a parent‐ or teacher‐completed indirect measure that includes broad competencies, adaptive functions and problems in children (Achenbach & Rescorla, 2001).

After identifying the final corpus of eligible studies, outcomes were initially separated in to either direct or indirect measures of EF to separate synthesis. This dichotomy reflects a distinction in the measurement of EF between performance, task‐related data (e.g., NEPSY tasks; direct measurement) and that of itemised, self‐ or other‐reported data (e.g., BRIEF questionnaire; indirect measurement). These forms of measurement were separated as there is evidence demonstrating poor convergence between direct and indirect measures of EF across a range of populations (e.g., Toplak et al., 2008) and in children with FASD (Gross et al., 2015; Mohamed et al., 2019). Thus, these measures were separated to ensure that only homogenous outcomes were synthesised together.

Once separated into direct and indirect measures, outcomes were further categorised into conceptually analogous EFs. Terminology in the EF literature is often used interchangeably, at times producing unclear distinctions between different function classifications. This is compounded by variation in the names of assessment measures and sub‐tests which can measure different components of EFs. To ensure studies were classified and synthesised accurately, the specific tasks assessed for each outcome measure reported in an eligible study were assessed against the conceptual definitions and guidance provided by Diamond (2013) and Miyake et al. (2000). Following this classification, a qualified neuropsychologist was then consulted on the categorisations and various psychometric papers were then used to further refine EF classifications. The final classification of each outcome included in this review is provided (see Table 1).

**Table 1:** Direct measures of executive functions

Measure	Task	Outcome classification
TEA‐Ch (score!)	Children keep a count of the number of target ‘scoring’ sounds presented across multiple trials, as if keeping the score on a computer game.	Attention (auditory)
TEA‐Ch (code transmission)	Children are presented with long lists of numbers and required to recall a single number that occurs before two consecutively presented sevens.	Attention (auditory)
NEPSY (auditory attention)	Children are presented with prerecorded wordlists and required to touch an appropriate circle in a book when a target word is presented.	Attention (auditory)
KiTAP (Ghost's Ball, omission errors)	Children are presented with series of ghosts of different colours in different spatial locations and press a key if two consecutive ghosts are the same colour (duration = 10 min). Errors of omission (not pressing the key when appropriate) are considered measures of inattention. Standard scores are used in scoring and interpretation, whereby higher scores equate to lower levels of inattention.	Attention (visual)
KiTAP (The owls, omission errors)	Children are presented visual and auditory stimulus and press a key when they either hear an owl make an 'incorrect' sequence of tones or when the owl on the screen closes its eyes. Errors of omission (not pressing the key when appropriate) are considered measures of inattention.	Attention (visual)
TOVA (omission errors)	Children are presented with a black screen and given a clicker in their dominant hand. A white square flashes on the screen with a small black square presented in the top or bottom section of the square. Children are required to click the clicker, only when the black square appears at the top of the white square. Errors of omission (not clicking when the white square appears at the top) are considered measures of inattention. Standard scores are used in scoring and interpretation, whereby higher scores equate to lower levels of inattention.	Attention (visual)
TEA‐Ch (Sky Search, DT)	Children are presented with a visual array of similar objects (flying spaceships) and identify perceptually similar, but unique types of spaceships. This required discrimination of target objects among similar distractors. Children are also required to circle similar pairs of objects to control for the impact of motor speed.	Attention (visual)
TEA‐Ch (creature counting)	Children are presented with ‘creatures’ in a tunnel and required to count them. When they encounter an arrow, they are required to verbalise the direction (up or down) and change the direction of their count. Down arrows signify that children need to count backwards from the previous count. Counting time and number of correct responses are considered indicators of performance for this measure.	Cognitive flexibility
NEPSY (inhibition/switching)	Children are presented with a page of black arrows/shapes and are required to verbally name the opposite direction/shape (e.g., circle/square) when the stimulus is coloured white or the correct direction/shape when coloured black.	Cognitive flexibility
CANTAB (intra/extra dimensional shift task)	The task involves presentation of stimuli which vary on either one (initially) or two (later trials) dimensions (e.g., white lines of different shapes, then white lines overlaid on various pink shapes). The rule which determines which stimulus should be selected is altered after six correct responses.	Cognitive flexibility
NIH toolbox (dimensional change card sort)	Children are presented with sets of cards which vary along two dimensions (colour and shape). In each trial, children are instructed to select a target that corresponds to a specific dimension. Rules for selection are changed seemingly randomly throughout the task.	Cognitive flexibility
KiTAP (Ghost's Ball, commission errors)	Children are presented with series of ghosts of different colours in different spatial locations and press a key if two consecutive ghosts are the same colour. Commission errors (pressing the key when not appropriate) are considered a measure of response inhibition. Standard scores are used in scoring and interpretation, whereby higher scores equate to lower levels of inhibition.	Response inhibition
KiTAP (The owls, commission errors)	Children are presented visual and auditory stimulus and press a key when they either hear an owl make an 'incorrect' sequence of tones or when the owl on the screen closes its eyes. Commission errors (pressing the key when not appropriate) are considered a measure of response inhibition.	Response inhibition
KiTAP (Happy‐Sad Ghost, commissions)	Children are presented with visual representations of ghosts and required to press a key when ghosts appear frowning, but not when smiling. They are simultaneously required to ignore distracting stimuli in the periphery.	Response inhibition
ANT‐C (incongruent)	Children are presented with either a cue (informing the child about the timing, location or both of a target) or a blank screen (depending on condition). Following this, a fish (the target) is presented above or below fixation and is surrounded by identical fish pointing in the same direction (congruent trial) or the opposite direction (incongruent trial) to the target fish. Children are required to hit a button to specify which direction the target is facing. Incongruent trials are considered a measure of response inhibition.	Response inhibition
KiTAP (Dragon's Castle, commissions)	Participants must allow blue and green dragons into the castle in alternating colour order by pressing one button for each colour, but the dragons appear in alternating locations, requiring the children to flexibly monitor which button to press. Commission errors occur when children press the button inappropriately and are considered a measure of response inhibition.	Response inhibition
TEA‐Ch (Map Mission)	Children are presented with a map filled with diverse sets of symbols, words and locations. They are given 60 s to identify specific symbols. The number of targets correctly identified in a 60 s interval is the primary indicator of performance. This task requires children to filter information to detect relevant stimuli and reject irrelevant stimuli.	Attentional inhibition
TEA‐Ch (Sky Search, attention score)	Children are presented with a visual array of similar objects (flying spaceships) and identify perceptually similar, but unique types of spaceships. This required discrimination of target objects among similar distractors. Children are also required to circle similar pairs of objects to control for the impact of motor speed.	Attentional inhibition
NIH toolbox (flanker)	Children are required to select the direction of a central target arrow among a group of distractor arrows. On congruent trials distractors face the same way as the target, on incongruent trials distractors face the opposite direction to target.	Response inhibition
TOVA (commission errors)	Children are presented with a black screen and given a clicker in their dominant hand. A white square flashes on the screen with a small black square presented in the top or bottom section of the square. Children are required to click the clicker, only when the black square appears at the top of the white square. Errors of commission (clicking when the white square appears at the bottom) are considered a measure of response inhibition. Standard scores are used in scoring and interpretation, whereby higher scores equate to better inhibition (i.e., lower impulsivity).	Response inhibition
NEPSY (inhibition/inhibition combined)	Child verbally names squares/circles and up/down but names the opposite (e.g., names squares circles).	Response inhibition
CANTAB (stocking of Cambridge task)	Children are shown a coloured display with balls in a specific order, they are required to move another set of coloured balls, one at a time to reflect the same order as the display balls in as few moves as possible.	Planning
NIH toolbox (list sorting)	Children are presented with a series of auditory and visual stimuli in a particular order and required to reproduce the order verbally in the absence of the original stimuli.	Working memory

Studies with any length of follow‐up were included in the review. To synthesise data from studies with different lengths of follow‐up, studies were classified into the following time‐frames analysed separately: short (0−3 months), medium (>3−6 months) and long‐term follow‐up (>6 months).

##### 4.1.4.2 Secondary outcomes

No secondary outcomes were included in the review.

### 4.2 Search methods for identification of studies

A number of sources were searched to ensure comprehensive coverage of the literature. The final search strategy included key psychological and allied health electronic databases, trial registers and grey literature sources. The reference lists of reviews relevant to the review topic were harvested, along with the references lists of studies included in the review. Studies included in the review were also used to conduct forward citation searching in Google Scholar. An additional hand‐search was conducted using key journal titles, covering a period of 12 months before execution of the search date to capture published articles not yet indexed in academic databases during the search period. As FASD was not adequately described before 1973, the search was limited to studies published after 1972. There were no restrictions placed on publication status or language. Each search is reported in Supporting Information: Appendix 1, as per current recommended guidelines (Kugley et al., 2017).

#### 4.2.1 Search terms and structure

Search terms were pilot‐tested and refined based on the rate of eligibility for each iteration, with the final search reflecting the search providing optimum sensitivity and specificity. The search implemented for PsycINFO (OvidSp) is provided below, as an example, and the exact search structure for each search provided in Supporting Information: Appendix 1

1. (executive* adj3 (function* or control* or atten*)).ab,hw,id,ot,ti.
2. (self adj3 regulat*).ab,hw,id,ot,ti.
3. (emotion* adj3 (regulat* or inhibit*)).ab,hw,id,ot,ti.
4. (cognitive adj3 flexib*).ab,hw,id,ot,ti.
5. (‘effortful control’ or ‘working memory’ or ‘set shift*’ or reasoning or planning or attention or inhibit*).ab,hw,id,ot,ti.
6. 1 or 2 or 3 or 4 or 5
7. (alcohol* adj3 (prenatal* or foetal or foetal or fetus* or foetus* or ‘neurodevelopment disorder*’ or ‘birth defect’ or ‘spectrum disorder*’)).ab,hw,id,ot,ti.
8. ‘sentinel facial feature*’.ab,hw,id,ot,ti.
9. foetal alcohol spectrum disorders.mh.
10. 7 or 8 or 9
11. (RCT or randomi* or trial* or experiment* or interven* or therap* or treat* or program* or review* or ‘meta‐analy*’ or ‘meta analy*’).ab,hw,id,ot,ti.
12. 6 and 10 and 11

#### 4.2.2 Electronic searches

The search encompassed January 1972 to December 31, 2020, with specific search dates provided in Supporting Information: Appendix 1. The following electronic databases were searched, with the database platform provided in parentheses:

- Scopus
- PubMed
- Embase (Elsevier)
- PsycINFO (OvidSp)
- PsycEXTRA (OvidSp)
- CINAHL (EBSCOhost)
- ProQuest Platform: Dissertations and Theses Global, Family Health, Psychology Journals, Health and Medical Complete, Nursing and Allied Health, Social Services Abstracts
- Web of Science Platform: Social Science Citation Index, Conference proceedings Index, Current Contents Connect
- Cochrane and Campbell Collaboration libraries

#### 4.2.3 Searching other resources

The following additional sources were systematically searched for the review.

*Websites and research repositories*

- Alcohol and Drug Foundation; URL: https://adf.org.au/
- Alcohol Concern (UK) [now Alcohol Change]; URL: https://alcoholchange.org.uk/
- Australian Centre for Child Protection; URL: https://www.unisa.edu.au/Research/Australian-Centre-for-Child-Protection/
- Australian Research Alliance for Children and Youth; URL: https://www.aracy.org.au/
- CanFASD (Canada FASD Research Network)
- FASD outreach; URL: https://canfasd.ca/
- FASDHub Australia; URL: https://www.fasdhub.org.au/
- Fetal Alcohol Network NZ; URL: https://www.fasd-can.org.nz
- Foundation for Alcohol Research and Education; URL: https://fare.org.au/
- National Institute on Alcohol Abuse and Alcoholism; URL: https://www.niaaa.nih.gov/
- NoFAS.org; URL: www.nofas.org
- NoFASD Australia; URL: https://www.nofasd.org.au/
- Royal Australasian College of Surgeons; URL: https://www.surgeons.org/en
- Russell Family Fetal Alcohol Disorders Association; URL: https://rffada.org/
- Social Science Research Network; URL: https://www.ssrn.com/index.cfm/en/
- Society for Research on Child Development; URL: https://www.srcd.org/
- The Alcohol Pharmacology Education Partnership; URL: https://sites.duke.edu/apep/

One website included in the review protocol (Betts et al., 2019) was no longer available when the systematic search was executed: Alcohol Science Database (ETOH).

*Conference proceedings*

- Australasian FASD Conference; URL: http://fasdconference.com/
- International Research Conference on Adolescents and Adults with FASD; URL: https://interprofessional.ubc.ca/initiatives/fasd2020/
- International Conference on FASD; URL: https://www.emedevents.com/c/medical-conferences-2019/8th-international-conference-on-fetal-alcohol-spectrum-disorder

*Trial registries and hand‐searches*

The following journals were hand‐searched:

- Research in Developmental Disabilities
- Developmental Neurorehabilitation
- Alcoholism: Clinical and Experimental Research
- Child Neuropsychology
- Applied Neuropsychology: Child

The following trial registries were searched:

- Australia and New Zealand Clinical Trials Registry
- ClinicalTrials.gov
- Clinical Trials Results
- Cochrane Central Register of Controlled Trials (CENTRAL)
- ISRCTN Registry (controlled-trials.com)
- NIH RePORTER
- Trials Register of Health Interventions (TRoPHI)
- UK Clinical Research Network (UKCRN Study Portfolio)
- WHO International Clinical Trials Registry

As a final search step, authors of eligible and ongoing studies were contacted to identify any unpublished studies.

### 4.3 Data collection and analysis

#### 4.3.1 Selection of studies

##### 4.3.1.1 Title and abstract screening

All records retrieved by the systematic search were imported to Endnote for removal of duplicates and ineligible document types (e.g., blog posts, book reviews) and the remaining records were exported to *SysReview* (Higginson & Neville, 2014) for screening and coding. Title and abstract screening was completed independently by two review authors (J.B., E.E.) using the following exclusion criteria:

1. Duplicate document
2. Ineligible document type (e.g., book reviews, blog posts)
3. Document does not relate to PAE in humans

Records not excluded at the title and abstract screening stage progressed to full‐text literature retrieval. In the case that full‐text documents could not be retrieved through existing university pipelines, they were ordered through university libraries or authors were contacted for provision of documents.

##### 4.3.1.2 Full‐text eligibility screening

All records retained through title and abstract screening were screened based on the following exclusion criteria (see Supporting Information: Appendix 2 for further details on the standardised screening form):

1. Duplicate document
2. Ineligible document type
3. Document is published before 1973
4. Document does not relate to PAE in humans
5. Ineligible participants
6. Ineligible intervention/no intervention
7. Ineligible outcome measure(s)
8. Ineligible methodological design

The first three criteria were a final check that duplicates, ineligible document‐types and studies unrelated to human PAE had not progressed through to full‐text screening. A random sample of 10% of documents were double‐screened blindly and independently by two authors (J.B., E.E.), with 99% agreement on inclusion/exclusion for this sample. As such, independent screening was conducted for the remainder of documents by two authors (J.B., E.E.). Where an inclusion/exclusion decision could not be made, the relevant screener discussed the study with the other screener. A third author (S.D.) was consulted in the case of disagreement until consensus was achieved.

#### 4.3.2 Data extraction and management

Studies retained following both stages of screening were coded in *SysReview* as per the standardised coding form and relevant risk of bias tool (see section below). Two authors independently coded all eligible studies (J.B., N.C.H.) and discrepancies were resolved through discussion with a third author (E.E., S.D.) until consensus was reached. Studies were coded along the following broad dimensions (see Supporting Information: Appendix 3 for full coding form):

1. Document description (e.g., location, year of publication)
2. Methodological issues (e.g., design, randomisation)
3. Participant information (e.g., child diagnosis, descriptives)
4. Intervention (e.g., training/education, setting, duration)
5. Outcomes (e.g., type of EF, measure used, time‐points)
6. Effect size (e.g., type of effect size, how it was obtained)

#### 4.3.3 Assessment of risk of bias in included studies

The Cochrane Risk of Bias Tool (RoB, Version 2.0; Sterne, 2016a) was used to assess risk of bias for RCTs. Using the signalling questions and guidance provided in the tool, studies were rated as having low risk, some concerns or high risk along the following domains: randomisation process, deviations from intended interventions, missing outcome data, measurement of the outcome and selection of the reported result. Where studies did not use random allocation to treatment and comparison conditions, the Cochrane tool for assessing Risk of Bias in Non‐Randomised Studies of Interventions (ROBINS‐I; Sterne, 2016b) was used. Using the signalling questions and guidance provided in the tool, non‐randomised comparison group studies were rated as having low, moderate, serious or critical risk along the following domains: bias due to confounding, selection of participants, classification of interventions, deviations from intended interventions, missing data, measurement of outcomes and selection of the reported result. The review protocol specified additional biases to be assessed for cluster randomised designs (e.g., recruitment bias, baseline imbalances, loss of clusters and incorrect analyses; Deeks et al., 2022), however, the review did not identify any studies using this design.

Studies using a single‐group pre‐ and post‐intervention design were not formally assessed for risk of bias, particularly in light of the review identifying RCTs and rigorous quasi‐experiments. Although additional considerations have been developed for assessing follow‐up and interrupted time‐series studies with ROBINS‐I, Sterne et al. (2020) argue that studies with a single pre‐ and post‐intervention measure of eligible outcomes are a particular case of uncontrolled before‐after studies generally considered to be at serious or critical risk of bias. The predominant concern with this type of study design is establishing whether changes in outcomes before and after the intervention are due to the intervention or other time‐varying confounders (Paulus et al., 2014; Sterne et al., 2020).

All studies were assessed independently by two authors against their relevant risk of bias tool (J.B., E.E.). Discrepancies were discussed with a third author (S.D.) until consensus was achieved. Coders were not blinded to study details during coding (e.g., author names, institutions, journals and results), as research suggests little benefit of blinding coders because they are often familiar with research that is relevant to the review (Kjaergard et al., 2001).

#### 4.3.4 Measures of treatment effect

**Figure 1:**
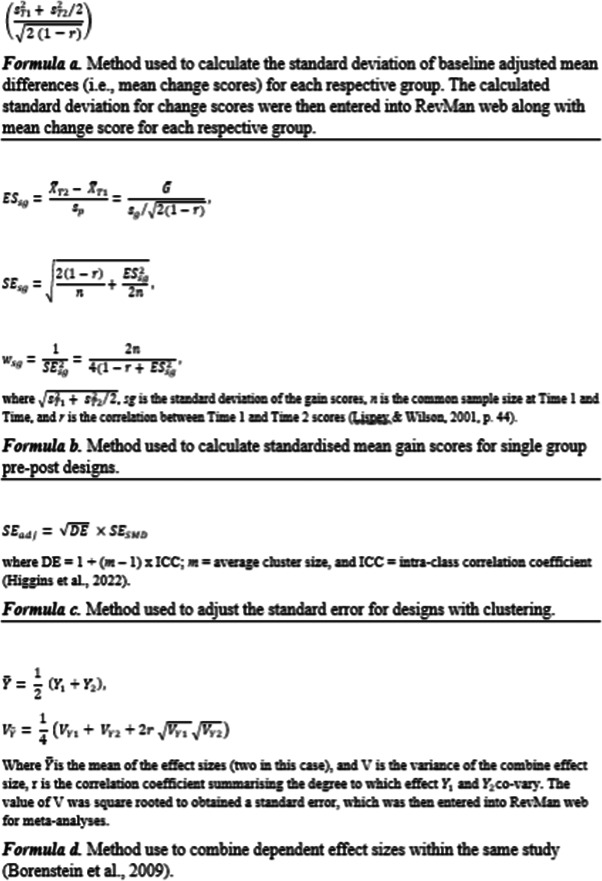
Statistical formulae

Treatment effects were reported as continuous outcome data for all studies. For RCT and quasi‐experimental studies with two independent groups, treatment effects were estimated using standardised mean differences (SMDs) by entering the required data into RevMan (*M*, SD, *n*). Where studies reported baseline and post‐intervention outcome data, SMDs were calculated using baseline adjusted mean differences (i.e., mean change scores), using the formula provided by Lipsey and Wilson (2001) if the standard deviation of the change score was not reported by study authors (see Figure 1, Formula a). In cases where authors did not report the correlation between pre‐post scores, existing research was consulted to obtain test–retest reliabilities. Where test–retest reliability could not be identified, a value of 0.5 was used for *r*.

For studies using a single‐group pre‐ and post‐intervention design, the SMD and standard errors were calculated using the formulae specified by Lipsey and Wilson (2001) for calculating standardised mean gain scores, which incorporate the correlation between baseline and post‐intervention outcomes (see Figure 1, Formula b). The same aforementioned procedure was used to obtain or estimate the correlation between baseline and post‐intervention measures. If studies directly reported effect sizes, these were used in analyses comprised of the single‐group studies.

#### 4.3.5 Unit of analysis issues

The unit of analysis for this review is each study, whereby secondary reports of studies were nested under the first published report of the study. We also anticipated that some studies would report repeated post‐intervention outcome measures. Where studies reported repeated post‐intervention outcome measures, studies were still nested under the primary study, but each time‐point was coded separately and categorised into the following time‐frames for separate syntheses: short (0–3 months), medium (>3–6 months) and long‐term follow‐up (>6 months).

In cases where studies included multiple types of experimental groups, those which most closely matched inclusion criteria were included, and others excluded (Deeks et al., 2022). Where studies employ clustering (e.g., cohorts of treatment groups), data for individuals are not independent due to dependence within clusters and can lead to a unit of analysis error if data are analysed at the individual level. When identified eligible studies contained clustering, the appropriateness of the authors' statistical analysis (e.g., multi‐level modelling) was first assessed. In the case that the statistical analysis had not taken into account the clustering, the approach recommended by Higgins et al., 2022 (Figure 1, Formula c) was used to adjust the standard error of the effect size before meta‐analysis.

Where there were multiple measures of the same construct reported in a study, conceptually similar outcomes were aggregated to form a composite effect size for that specific EF using the approach provided by Borenstein et al. (2009). Specifically, the effect sizes were averaged and an adjusted standard error was calculated, both of which were then used in the relevant meta‐analysis (Figure 1, Formula d). Direct (standardised/normed neuropsychological assessments) and indirect (e.g., parent self‐report questionnaires) measures of executive were not combined, even in the case that they measured the same EF. This is due to differences in measurement error between these methods (Gross et al. 2014). Thus, dependent direct and indirect outcome measures were combined and synthesised separately.

#### 4.3.6 Dealing with missing data

The standardised coding form instructed the coder to record when data was missing from study reports to facilitate the location of the missing data. If data was missing from eligible studies, the study authors were contacted to either clarify ambiguous data or obtain data that was completely missing. Where missing data for effect size calculation could not be obtained, the study was excluded from the relevant meta‐analyses, but the study was still coded and included in the narrative summary of eligible studies. The results section explicitly reports which data is missing and the discussion sections note the potential impact of missing data of the review findings (Deeks et al., 2022).

#### 4.3.7 Assessment of heterogeneity

All studies were initially analysed together using a random effects model and heterogeneity was examined using the *I* ^2^ statistic, *χ* ^2^ test and *τ* ^2^ (Deeks et al., 2022).

#### 4.3.8 Assessment of reporting biases

To minimise publication or reporting bias, this review draws from a comprehensive search of published and published literature. The review protocol (Betts et al., 2019) specified the use of funnel plots and subgroup analyses based on publication status, however, insufficient studies were located to conduct these analyses (Page et al., 2019). Where reporting biases were detected in studies (i.e., reporting only data for significant effects) this is specified in text and considered in the risk of bias assessment for included studies.

#### 4.3.9 Data synthesis

After coding each study and extracting/calculating each effect size, RevMan (version 5.5) was used to conduct random effects inverse variance meta‐analyses with 95% confidence intervals (CIs). If at least two studies with conceptually similar interventions, participants and outcomes were identified, a meta‐analysis was conducted. This resulted in 10 meta‐analyses across five EFs for direct measures and five EFs for indirect measures. If a study was considered too conceptually different to be included in meta‐analyses, single effect sizes were calculated and reported. Single effect sizes and those derived from meta‐analyses were interpreted using Cohen's rule of thumb, whereby 0.2 represents a small effect, 0.5 a medium effect and 0.8 a large effect (Cohen, 1988).

#### 4.3.10 Subgroup analysis and investigation of heterogeneity

The review protocol outlined the intended approach for investigating heterogeneity through specific sub‐group analyses (Betts et al., 2019 and see Section 3). However, the review located too few studies to conduct these statistical analyses. Where heterogeneity was detected, the results integrate a discussion of the differences between the studies based on study coding across the a priori subgroups. For future updates of this review, the protocol procedures will be used to investigate heterogeneity through sub‐group analyses.

#### 4.3.11 Sensitivity analysis

The review protocol outlined the intended approach for conducting sensitivity analyses to examine how factors in the review decision‐making process may have impacted the robustness of the meta‐analytic results (e.g., combining vs. not combing RCTs and quasi‐experiments, removing studies with high risk of bias from meta‐analyses). Only one sensitivity analysis was possible based on the studies located for the review. Sensitivity analyses were used to assess the impact of estimating intra‐class correlation coefficients (ICC) for studies with clustering on the results of meta‐analyses. Of the randomised and two‐group quasi‐experimental studies using direct measures of EFs, only one study—Nash (2012)—had clustering. The authors did not report the ICC, so we conducted sensitivity analyses as suggested by Deeks et al. (2020), which has been implemented in analogous reviews (Armstrong et al., 2018; Barlow et al., 2016). Barlow et al. (2016) located an ICC of 0.03 for one included group‐based psychological intervention study. An ICC of zero was used for meta‐analyses, with accompanying sensitivity analyses to assess difference in meta‐analysis results for ICCs of 0.02, 0.03 and 0.1. For future updates of this review, all the variables and procedure specified in the protocol will be used for sensitivity analyses (Betts et al., 2019).

## 5 RESULTS

### 5.1 Description of studies

#### 5.1.1 Results of the search

**Figure 2:**
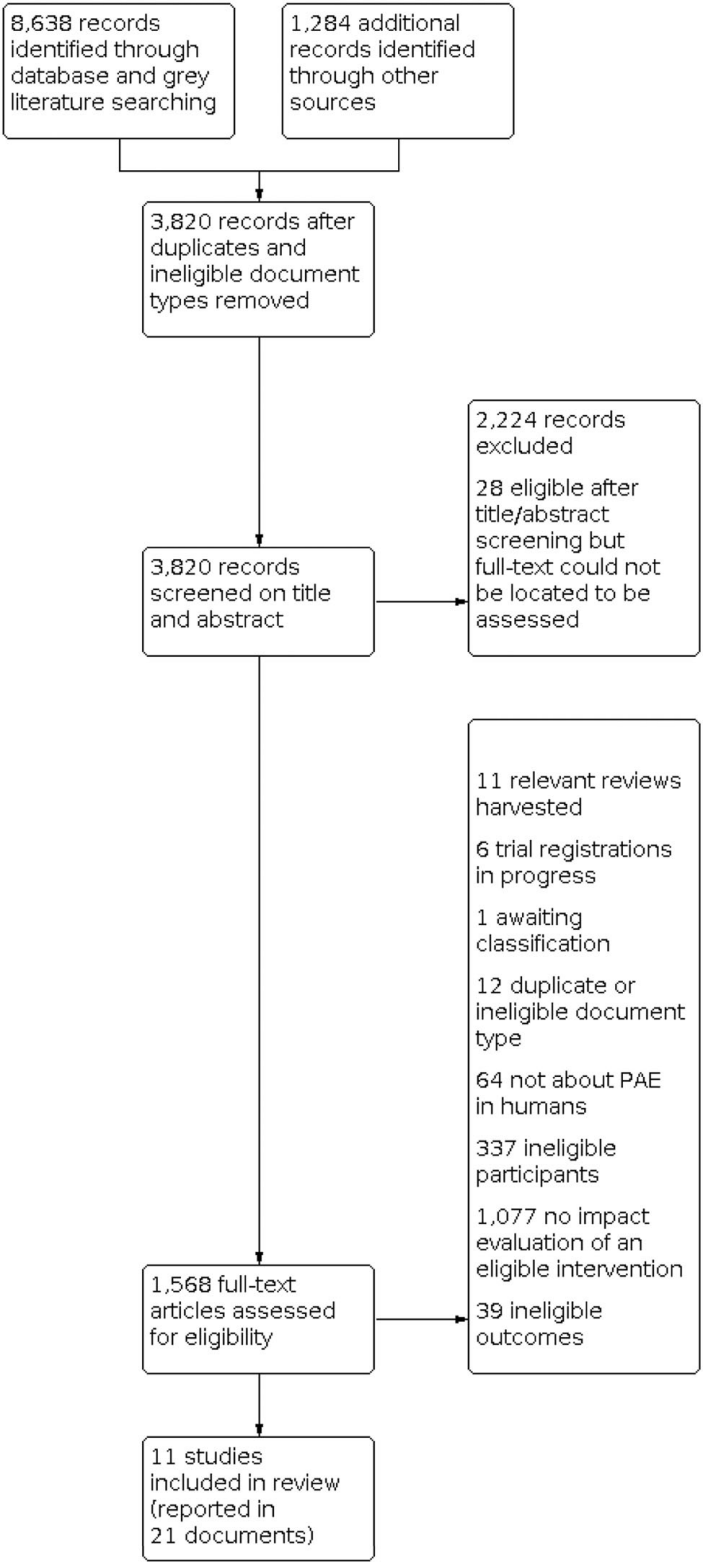
Study flow diagram

Figure 2 summarises the results of search and screening for the review. The systematic search of databases and grey literature sources identified 8638 records and the hand‐searches, reference harvesting, and forward citation searching identified an additional 1284 records. After duplicate removal, a corpus of 3820 records were imported into *SysReview* (Higginson & Neville, 2014) for screening. A total of 1596 records were screened on their title and/or abstract as being potentially about human PAE and were progressed to the full‐text literature retrieval stage.

For 28 records screened as potentially eligible after title and abstract screening, either the full‐text document could not be obtained or the full‐text document was insufficient for confirming eligibility for the review. In all cases, attempts were made to locate documents by ordering through institutional libraries or contacting study authors, but these procedures were unsuccessful. These 28 records are included in the ‘Studies awaiting classification’ reference list. A full‐text was obtained for the remaining 1568 records, which were then screened for final eligibility. Of these, 11 were reviews that were harvested for studies (Bertrand, 2009; Hanscom, 2008; Hanson, 2000; Kodituwakku & Kodituwakku, 2011; Landes et al., 2017; McLean, 2014; Ospina et al., 2011; Peadon et al., 2009; Premji et al., 2007; Reid, 2015; Schaffer & Geva, 2016). A further 6 documents were trial registries for studies that were verified as either ongoing or in data‐analysis stage based on correspondence with study authors, and these are listed in the ‘ongoing studies’ reference list. A single study was deemed potentially eligible, depending on the percentage of the sample with FASD diagnoses, yet correspondence from authors was outstanding at the time of submission (Wagner et al., 2020).

Of the remaining 1550 documents, 1529 were excluded after confirmation that the document/study (a) was a duplicate (*n* = 12); (b) did not relate to PAE in humans (*n* = 64); (c) utilised ineligible participants (*n* = 337); (d) reported ineligible outcomes (*n* = 39); or (e) did not report on or evaluate an eligible intervention using an eligible research design (*n* = 1077). A full list of the excluded studies is available from the authors by request. The remaining 21 documents deemed eligible for the review reported on 11 unique studies. Of these 11 studies, there was variation in the sufficiency of the data to calculate effect sizes for all eligible outcomes, which is reported by outcome in the relevant ‘Effects of interventions’ subsection. Attempts were made to contact authors of studies with insufficient data for effect size calculation, with only one group of study authors able to provide the data required. All 11 eligible studies are included in the study summary tables and included studies section below.

#### 5.1.2 Included studies

A total of 253 participants were included across the 11 included studies (in 21 study reports). The subsections below provide an overview of the included studies with further detail provided in the summary tables for each included study and abbreviated study summaries provided in Table 2.

**Table 2:** Abbreviated study summary table

Study	Design and location	Participants	Intervention
Coles et al. (2015)	RCT United States	Sample: Children with a clinical diagnosis of FAS, partial FAS or significant alcohol‐related physical deformities Total referred sample: 20 Child age (years; *M*/SD): Exp = 7.5 (1.4); Con = 7.0 (1.6) Child gender (% female): Exp = 30%; Con = 30%	Name of intervention: GoFAR
			Setting: Unclear
			Format:
			Individual face‐to‐face parenting sessions Individual child computer game sessions Parent‐and‐child therapy sessions
Hutchison (2015)	Single group pre‐post Canada	Sample: Aged 6–13 years, diagnosis of FASD or receipt of services related to PAE Total referred sample: 10 Age (years; *M*/SD): 10 (range: 6–12 years, no SD reported) Gender (% female): 50%	Name of intervention: The Caribbean Quest (CQ) Setting: School Format: Child computer game sessions (group/individual format not reported)
Kerns et al. (2010)	Single group pre‐post Canada	Sample: Children with FASD diagnosis Total referred sample: 12 Age (years; *M*/SD): 12.3 (2.67) Gender (% female): 40%	Name of intervention: Computerised Progressive Attention Training Setting: School (library or special education area) Format: Individual child computer game sessions
Leung et al. (2015)	Single group pre‐post Canada	Sample: Children with confirmed history of PAE Total referred sample: 27 Child age (years; *M*/SD): 9.2 (2.59) Child gender (% female): 55%	Name of intervention: Cogmed^©^ Working Memory Intervention Setting: Family home Format: Individual face‐to‐face child computer game sessions
Loomes et al. (2008)	Matched quasi‐experimental Canada	Sample: Children previously diagnosed with FASD Total referred sample: Not reported (*n* = 33 tested) Age (years; *M*/SD): Exp = 7 years 5 months (2.32); Con = 7 years 6 months (1.23) Gender (% female): Exp = 52.94%; Con = 31.25%	Name of intervention: Rehearsal training Setting: Unclear Format: Individual face‐to‐face child sessions
Nash (2012)	RCT Canada	Sample: Child aged 8–12 years, previous FASD diagnosis, IQ > 70 Total referred sample: 29 (14 assigned to Exp; 15 assigned to Con) Age (years; *M*/SD): Exp = 10.3 (1.7); Con = 10.4 (1.3) Gender (% female): Exp = 50%; Con = 46%	Name of intervention: *Alert Program for Self Regulation*® Setting: Hospital clinic Format: Individual face‐to‐face child sessions
Pei et al. (2011)	RCT Canada	Sample: children reported to have diagnosis of FASD Total referred sample: 18 Age (years; *M*/range): Exp = 9.12 (range: 6–12); Con = 9.78 (6–12) Gender (% female): Exp = 77.77%; Con = 66.66%	Name of intervention: Cognitive Carnival with meta‐cognitive strategy coaching Setting: School Format: Individual computer game and meta‐cognitive training sessions
Petrenko et al. (2017)	RCT United States	Sample: Children with FASD diagnosis between 4 and 8 years and their caregiver(s) Total eligible referred participants: 30 (Exp = 19; Con = 11) Age (years; *M*/SD): Exp = 6.52 (1.31); Con = 6.59 (1.28) Gender (% female): Exp = 37.5%; Con = 7.1%	Name of intervention: Families on Track
			Setting: Family home (parent consultation component) and university/community settings (child skills group component)
			Format:
			Individual face‐to‐face parent consultations (weekly, 90 min each) Group face‐to‐face child skills groups (fortnightly, 90 min each)
Reid et al. (2017)	Single group pre‐post Australia	Sample: Children with FASD diagnosis and their caregiver(s) Participants beginning treatment: 3 Age (years): Child 1 = 9; Child 2 = 12; Child 3 = 11 Gender (% female): 100%	Name of intervention: Parents under Pressure (PuP), adapted Setting: Family home/clinic (family's choice) Format: Parent‐and‐child therapy sessions
Vernescu (2008)	RCT Canada	Sample: Children with FASD diagnosis Total referred sample: 20 Age (years; *M*/SD): Exp = 9.54 (1.58); Con = 9.5 (1.62) Gender (% female): 55% (group split not reported)	Name of intervention: *Pay Attention!* Program Setting: School Format: Individual face‐to‐face child sessions
Wells et al. (2012)	RCT United States	Sample: Children aged 6–11:9 years who met criteria for FAS, partial FAS or ARND Total referred sample: 90 Age (years; *M*/SD): Exp = 8.12 (1.48); Con = 9.24 (1.36) Gender (% female): Exp = 32.5%; Con = 31.6%	Name of intervention: Neurocognitive Habilitation Program Setting: Unclear
			Format:
			Group face‐to‐face caregiver psychoeducational sessions Group face‐to‐face child intervention sessions Caregiver‐and‐child practice sessions

Abbreviations: FAS, foetal alcohol syndrome; FASD, foetal alcohol spectrum disorder; RCT, randomised controlled trial.

##### 5.1.2.1 Document type, funding and study location

Studies were mainly reported in one or more journal articles (*k* = 14), with a smaller number reported in theses (*k* = 4), technical reports (*k* = 1), trial registries (*k* = 1) or conference abstracts (*k* = 1). Eight studies reported funding support for either the intervention or evaluation (Coles et al., 2015; Kerns et al., 2010; Loomes et al., 2008; Nash, 2012; Pei et al., 2011; Petrenko et al., 2017; Vernescu, 2008; Wells et al., 2012), predominantly from governmental departments or health research institutes. Funding was either not reported or not received for the remaining three included studies (Hutchison, 2015; Leung et al., 2015; Reid et al., 2017). Three studies were conducted in the United States (Coles et al., 2015; Petrenko et al., 2017; Wells et al., 2012), seven in Canada (Hutchison, 2015; Kerns et al., 2010; Leung et al., 2015; Loomes et al., 2008; Nash, 2012; Pei et al., 2011; Vernescu, 2008) and only one in Australia (Reid et al., 2017).

##### 5.1.2.2 Research design and comparison conditions

Six studies were RCTs (Coles et al., 2015; Nash, 2012; Pei et al., 2011; Petrenko et al., 2017; Vernescu, 2008; Wells et al., 2012), with some variation in randomisation strategies. One study used an alternating sequence of allocation (Nash, 2012), one study using block randomisation (Petrenko et al., 2017), one study randomised within matched pairs (Vernescu, 2008), one study used yoked pairs (Coles et al., 2015), one study used an allocation via a table of even and odd numbers (Wells et al., 2012), and one did not report the method used to achieve randomisation (Pei et al., 2011). One study used a quasi‐experimental design with matching on participant age and gender (Loomes et al., 2008), without further specification for how participants were allocated to the comparison or experimental conditions. Four studies used single‐group pre‐post intervention research designs (Hutchison, 2015; Kerns et al., 2010; Leung et al., 2015; Reid et al., 2017). One study used an ineligible comparison group, and as such was treated as a single‐group study in this review (Leung et al., 2015), along with a secondary report of the Pei et al. (2011) study which reported on the intervention group only. For the RCT/quasi‐experimental studies, three studies used a no treatment comparison condition (Coles et al., 2015; Loomes et al., 2008; Wells et al., 2012). Two studies used a treatment‐as‐usual comparison condition, comprised of either neuropsychological evaluation and customised community referrals (Petrenko et al., 2017) or individualised contact at the same level as the experimental participants, with usual individualisation of academic activities based on child needs and skills (Vernescu, 2008). The remaining studies used a wait list control comparison condition (Nash, 2012; Pei et al., 2011).

##### 5.1.2.3 Participant characteristics and recruitment

One included study did not report recruitment information (Vernescu, 2008). Six studies recruited participants via referrals from health practitioners or specialised clinics (Coles et al., 2015; Leung et al., 2015; Loomes et al., 2008; Nash, 2012; Petrenko et al., 2017; Reid et al., 2017). One study recruited through the Department of Children and Family Services (Wells et al., 2012) and three studies reported recruitment via advertisements/information packages sent home with children from school (Hutchison, 2015; Kerns et al., 2010; Pei et al., 2011).

All studies specified that participants needed to have a diagnosis or were identified as having FASD or confirmed PAE. Five studies required a formal diagnosis related to FASD (Kerns et al., 2010; Nash, 2012; Reid et al., 2017; Vernescu, 2008; Wells et al., 2012), with no further specification. Four studies required either a formal diagnosis or (i) a confirmed history of PAE (Leung et al., 2015; Petrenko et al., 2017); or (ii) significant alcohol‐related physical deformities (Coles et al., 2015); or (iii) receipt of services related to PAE (Hutchison, 2015). Two studies did not explicitly state eligibility criteria, however all children in the sample had received a formal diagnosis of a FASD‐related disorder (Loomes et al., 2008) or had been identified as having an FASD diagnosis by practitioners (e.g., teachers, Pei et al., 2011).

##### 5.1.2.4 Intervention characteristics

###### 5.1.2.4.1 Setting

Three studies did not report the setting in which the intervention took place (Coles et al., 2015; Loomes et al., 2008; Wells et al., 2012). Settings varied across other studies, including hospital clinic (Nash, 2012), family home (Leung et al., 2015), school (Hutchison, 2015; Kerns et al., 2010; Pei et al., 2011; Vernescu, 2008) or a combination of home/community/university settings (Petrenko et al., 2017; Reid et al., 2017).

##### 5.1.2.5 Implementing practitioners

Implementing practitioners in six of the 11 studies were allied health practitioners, such as psychologists or occupational therapists (Coles et al., 2015; Hutchison, 2015; Nash, 2012; Petrenko et al., 2017; Reid et al., 2017; Wells et al., 2012). The remaining five studies either used teachers and/or trained research staff to implement the intervention (Kerns et al., 2010; Leung et al., 2015; Loomes et al., 2008; Pei et al., 2011; Vernescu, 2008).

##### 5.1.2.6 Duration and intensity

All eligible studies reported the duration of the intervention; mean duration ranged from 11 days (Loomes et al., 2008) to 30 weeks (Petrenko et al., 2017). The number of intervention sessions ranged from 1 (Loomes et al., 2008) to 31 (Kerns et al., 2010), with most studies using a frequency of approximately one session per week.

##### 5.1.2.7 Format and nature of the intervention

Five studies delivered the intervention in individual format to children only (Kerns et al., 2010; Leung et al., 2015; Loomes et al., 2008; Nash, 2012; Pei et al., 2011; Vernescu, 2008), whereas four studies implemented a mixture of parent/child individual/group sessions (Coles et al., 2015; Petrenko et al., 2017; Reid et al., 2017; Wells et al., 2012). One study did not specify if the intervention was delivered in an individual or group format (Hutchison, 2015).

Five studies included a computerised component, with or without supplementary face‐to‐face EF training (Coles et al., 2015; Pei et al., 2011; Hutchison, 2015; Kerns et al., 2010; Leung et al., 2015). The remaining studies used face‐to‐face therapeutic or training strategies (Loomes et al., 2008; Nash, 2012; Petrenko et al., 2017; Wells et al., 2012), with Reid et al. (2017) supplementing therapy with technology‐facilitated activities.

Two studies evaluated the Alert Program (Nash, 2012) or an adaptation of the Alert program (Wells et al., 2012) designed to improve self‐regulation in children. Another study evaluated the GoFAR program, which uses meta‐cognitive techniques to teach children planning and organisation skills, individual parent behavioural regulation training and opportunities for supported practice (Coles et al., 2015). Vernescu (2008) evaluated the Pay Attention program, a set of training sessions designed to improve various attention‐related outcomes. Petrenko et al. (2017) evaluated the Families on Track intervention, comprised of a mixture of child skill sessions and parent therapy. Loomes et al. (2008) evaluated a form of rehearsal training, designed to improve working memory performance in children through teaching of problem solving strategies. Of the five single‐group studies, four assessed computerised games designed to challenge and improve aspects of EF (Hutchison, 2015; Kerns et al., 2010; Leung et al., 2015; Pei et al., 2011). Reid et al. (2017) evaluated an adaptation of the Parents under Pressure program, in which individualised case plans were developed for participants which focused on strategies aiming to improve self‐regulation in children.

##### 5.1.2.8 Fidelity

Regular supervision with study authors or therapists was the most common form of fidelity monitoring (Nash, 2012; Petrenko et al., 2017; Wells et al., 2012). Two studies used observations of children during the task, recording behavioural evidence of strategy use, and children were subsequently asked about their use of taught strategies (Hutchison, 2015; Loomes et al., 2008). Coles et al., 2015 used a more comprehensive monitoring method, recording a number of indicators (time spent playing intervention game, attention to game, enthusiasm, number of prompts required and also goals achieved), understanding of session content and homework completed in parental therapy sessions. Reid et al. (2017) integrated explicit fidelity checks into the intervention, asking participants to discuss how strategies had been implemented throughout training. Four studies did not specify the monitoring of fidelity (Kerns et al., 2010; Leung et al., 2015; Pei et al., 2011; Vernescu, 2008).

##### 5.1.2.9 Outcome characteristics

Table 1 shows how EFs were directly measured, and Table 3 provides an overview of the outcome measures by included study, highlighting how most included studies reported multiple measures of EFs. Please also see Table 4 for functional descriptions of each outcome. Seven included studies reported direct measures of EF, including: attention (auditory and visual; Coles et al., 2015; Hutchison, 2015; Kerns et al., 2010; Leung et al., 2015; Nash, 2012; Vernescu, 2008), inhibition (attentional and response; Coles et al., 2015; Hutchison, 2015; Kerns et al., 2010; Nash, 2012; Reid et al., 2017; Vernescu, 2008), cognitive flexibility (Hutchison, 2015; Leung et al., 2015; Nash, 2012; Reid et al., 2017; Vernescu, 2008), planning (Nash, 2012) and working memory (Hutchison, 2015; Kerns et al., 2010; Leung et al., 2015).

**Table 3:** Direct and indirect executive function outcome measures by included study

Study	Direct outcomes	Indirect outcomes
Coles et al. (2015)	Attention, auditory (NEPSY: auditory attention) Attention, visual (TOVA: omissions) Inhibition, response (NEPSY: inhibition‐inhibition) Inhibition, response (TOVA: commissions)	Global executive function (BRIEF) Behavioural regulation (BRIEF) Meta‐cognition (BRIEF) Effortful control (CBQ)
Hutchison (2015)	Attention, auditory (NEPSY: auditory attention) Cognitive flexibility (NEPSY: inhibition‐switching) Cognitive flexibility (NEPSY: response set) Cognitive flexibility (NIH toolkit: dimensional change card sort) Inhibition, response (NIH toolkit: Flanker) Inhibition, response (NEPSY: inhibition‐inhibition) Working memory visual (NIH toolkit: list sort working memory) Working memory, visual (WISC‐V: spatial span‐backwards) Working memory, verbal (WISC‐V: digit span‐backwards)	Global executive function (BRIEF) Behavioural regulation (BRIEF) Inhibition (BRIEF) Shift (BRIEF) Meta‐cognition (BRIEF) Working memory (BRIEF)
Kerns et al. (2010)	Attention, auditory (TEA‐Ch: score‐total) Attention, visual (KiTAP: Ghost's Ball‐omissions)^a^ Inhibition, response (KiTAP: owls) Inhibition, response (KiTAP: Happy/Sad Ghost‐commissions) Inhibition, response (KiTAP: Ghost's Ball‐commissions) Inhibition, response (KiTAP: Dragon's Castle‐commissions)^a^ Inhibition, response (ANT‐C: incongruent) Working memory, verbal (children's size ordering task) Working memory, visual (WISC‐V: spatial span‐backwards)	
Leung et al. (2015)	Attention, auditory (NEPSY: auditory attention) Attention, visual (TOVA: omissions) Cognitive flexibility (NEPSY: inhibition‐switching) Cognitive flexibility (NEPSY: response set) Inhibition, response (NEPSY: inhibition‐inhibition) Inhibition, response (TOVA: commissions) Working memory, verbal (AWMA: verbal working memory) Working memory, visual (AWMA: visual spatial working memory) Working memory, visual (WISC‐V: spatial span‐backwards)	Global executive function (BRIEF) Executive function (Conner's Scales) Behavioural regulation (BRIEF) Inhibition (BRIEF) Attention (Conner's Scales) Attention (BASC‐2) Shift (BRIEF) Meta‐cognition (BRIEF) Emotional control (BRIEF) Working memory (BRIEF) Planning (BRIEF) Organisation skills (BRIEF) Monitoring skills (BRIEF)
Loomes et al. (2008)	Working memory, verbal (digit span backwards task modelled off working memory test battery for children)	
Pei et al. (2011)	Meta‐cognition (checklist of strategies)^a^ Attention, auditory (NEPSY: auditory attention)^a^ Attention, visual (farm animals game: omissions)^a^ Attention/Inhibition (KiTAP, no specification of subtest reported)^a^ Inhibition (NEPSY; day/night task; computerised go/no‐go task; farm animals game (commissions); tasks of executive control)^a^ Working memory, visual (WISC‐IV: spatial span; WMTB‐C: block recall)^a^ Working memory, verbal (WMTB‐C: digit recall; tasks of executive control)^a^	General executive function (BRIEF, subscales not specified) Attention (Conner's Scales) Attention (Attention Deficit Disorders Evaluation Scale, ADDES)
Nash (2012)	Attention, auditory (TEA‐Ch: score) Cognitive flexibility (CANTAB: intra‐extra‐dimensional) Cognitive flexibility (NEPSY: inhibition‐switching) Inhibition, attentional (TEA‐Ch: Sky Search‐attention score) Inhibition, response (NEPSY: inhibition‐inhibition) Planning (CANTAB: stocking of Cambridge)	Global executive function (BRIEF) Behavioural regulation (BRIEF) Emotional control (BRIEF) Shift (BRIEF) Inhibition (BRIEF)
Petrenko et al. (2017)		Emotional control (emotion regulation checklist)
Reid et al. (2017)	Cognitive flexibility (NEPSY: inhibition‐switching) Inhibition, response (NEPSY: inhibition‐inhibition)	Global executive function (BRIEF) Behavioural regulation (BRIEF) Meta‐cognition (BRIEF)
Vernescu (2008)	Attention, auditory (TEA‐Ch: code transmission) Attention, auditory (TEA‐Ch: score) Attention, visual (KiTAP: Ghost's Ball‐omissions) Attention, visual (TEA‐Ch Sky Search, DT)^a^ Cognitive flexibility (TEA‐Ch: creature counting) Inhibition, attentional (TEA‐Ch: Sky Search‐attention score) Inhibition, attentional (TEA‐Ch: Map Mission)^a^ Inhibition, response (KiTAP owls)^a^ Inhibition, response (KiTAP: Dragon's Castle‐commissions) Inhibition, response (KiTAP: Happy/Sad Ghost‐commissions) Inhibition, response (KiTAP: Ghost's Ball‐commissions)^a^	
Wells et al. (2012)		Emotional control (BRIEF) Shift (BRIEF) Inhibition (BRIEF) Initiation (BRIEF) Monitoring (BRIEF) Working memory (BRIEF) Planning/organising (BRIEF) Organisation of materials (BRIEF)

^a^ Measured by authors, insufficient data for effect size calculation or data could not be obtained through contact with authors.

**Table 4:** Functional description of executive functions

Executive function	Functional description
*Direct*	
Attentional inhibition	The ability to stop automatic shifts in attention and shift attention back to tasks that are goal‐directed. This skill allows children to not become distracted by surroundings when working on a task, or to redirect attention back to the task if they do become distracted.
Response inhibition	The ability to stop automatic behavioural responses in favour of behaviours that are more goal‐directed for the situation. This skill allows children to stop themselves from calling out in class, for example.
Cognitive flexibility	The ability to tolerate moving between different contexts and understand how each new situation may include changes to rules and expectations and adapt behaviour or perspective accordingly.
Working memory	The ability to hold and integrate multiple pieces of information in mind for the purpose of solving problems or completing tasks. This skill allows children to complete tasks with multi‐step instructions or complete tasks such as mental arithmetic
Planning	The ability to create and carry out a series of steps in sequence to achieve a goal
*Indirect*	
Global executive function (GEC)	This is a composite measure created by adding performance across behavioural regulation, meta‐cognition and emotional control (all described below).
Behavioural regulation	The ability to understand the need for different behaviours in different situations and use this to stop inappropriate behaviours and emotions in favour of more goal‐directed actions
Meta‐cognition	The ability to independently plan and begin tasks, as well as monitor own thinking and progress. This allows children to actively solve problems across a variety of situations
Emotional control	The ability to understand the usefulness of different emotions across context and actively control which emotions are felt or expressed depending on certain rules
Shifting	The ability to tolerate moving between different contexts and understand how each new situation may include changes to rules and expectations and adapt behaviour or perspective accordingly.
Inhibition	The ability to stop a behaviour when deemed appropriate. This ability allows children to control their behaviour in different settings (e.g., not calling out in the classroom).
Initiation	The ability of a child to begin activities, generate ideas or problem solve on their own.
Monitoring	The ability of a child to assess their own performance following completion of a task, or assess the impact their actions may have on the feelings of others.
Working memory	The ability to hold and integrate multiple pieces of information in mind for the purpose of solving problems or completing tasks. This skill allows children to complete tasks with multi‐step instructions or complete tasks such as mental arithmetic
Planning/organising	The ability to understand demands of current and future tasks and activities. This skill allows children to predict future events, set goals based on predictions and step through sequential steps to achieve goals
Organisation of materials	The ability to organise physical materials useful in the pursuit of goals. This refers to things such as a child keeping a neat and tidy workspace or independently cleaning up their belongings.
Attention	The ability to direct conscious awareness towards things that matter. This allows a child to stay focused in class or continue working on a boring task.
Effortful control	The ability to consciously control ones behaviour or emotional response to the environment

Seven studies reported indirect measures of EF, including: Global EF (Coles et al., 2015; Leung et al., 2015; Nash, 2012; Reid et al., 2017), behavioural regulation (Coles et al., 2015; Hutchison, 2015; Leung et al., 2015; Nash, 2012; Reid et al., 2017), meta‐cognition (Coles et al., 2015; Hutchison, 2015; Leung et al., 2015; Reid et al., 2017), effortful control (Coles et al., 2015), emotional control (Leung et al., 2015; Nash, 2012; Petrenko et al., 2017; Wells et al., 2012), shifting (Hutchison, 2015; Leung et al., 2015; Nash, 2012; Wells et al., 2012), inhibition (Hutchison, 2015; Leung et al., 2015; Nash, 2012; Wells et al., 2012), initiation (Wells et al., 2012), monitoring (Leung et al., 2015; Wells et al., 2012), working memory (Leung et al., 2015; Wells et al., 2012), planning/organising (Leung et al., 2015; Wells et al., 2012) and organisation of materials (Leung et al., 2015; Wells et al., 2012), and attention (Leung et al., 2015).

All studies reported baseline outcome assessment and post‐intervention outcome assessment (immediately after to 3 months after the intervention), aside from one study which conducted post‐intervention measures approximately 6 months after the completion of the intervention (1 year after baseline; Petrenko et al., 2017). Three studies also reported follow‐up assessments of at least one outcome measure at 5 weeks (Leung et al., 2015), 3 months after completing the intervention (Reid et al., 2017) and 12 months after completion of the intervention (18 months after random assignment; Petrenko et al., 2017).

#### 5.1.3 Excluded studies

Due to the large number of full‐text records screened for eligibility, a comprehensive description of all excluded studies is not included here. Included in this section is discussion of a smaller subset of studies which met more than two inclusion criteria (*n* = 34). A total of 31 studies explored improving outcomes in children with FASD but did not evaluate an intervention exclusively or clearly focused on improving EF (Chandrasena et al., 2009; Baker, 2013; Coles et al., 2009; Coriale et al., 2013; Cyr, 2011; Davis et al., 2011; De villiers, 2005; Dorrie et al., 2014; Fernández‐Jaén, 2011; Hollar, 2012; Kodituwakku, 2010; Koren et al., 2014; Kully‐Martens et al., 2012, 2013, 2018; Millians & Coles, 2014; Moore & Tolle, 2008; Mulvihill, 2008; Olson & Montague, 2011; Paley & O'Connor, 2009, 2011; Peadon & Elizabeth, 2010; Petrenko et al., 2017; Sabou et al., 2012; Senturias & Burns, 2014; Singal et al., 2018; Timler et al., 2005; Turner et al., 2015; Watson & Westby, 2003; Zarnegar et al., 2016; Zevenbergen & Ferraro, 2001).

Example interventions that did not explicitly aim to improve EF included:

1. Mindfulness meditation: Participants engaged in a mindfulness meditation which involves directing attention to breathing and purposefully observing internal processes without judgement (Baker, 2013).
2. Social communication intervention: This included role‐plays of social scripts from both child and adult perspectives, and clinician modelling of socially appropriate verbal responses (Timler et al., 2005).
3. Strongest Families FASD: Participants completed a program adapted specifically for families with FASD children. Parents worked through a series of web‐based content, designed to equip parents with skills to better manage behavioural issues in children with FASD (Turner et al., 2015).
4. Therapeutic Horseback riding: Equine‐assisted therapy designed to improve awareness in children with FASD (De villiers, 2005).
5. The Math Interactive Learning Experience (MILE) Program: A program designed to improve mathematics ability in children through direct teaching of skill development in arithmetic (Coles et al., 2009; Kully‐Martens et al., 2013, 2018; Millians & Coles, 2014).
6. Individual therapy: Children with FASD engaged in therapy with licensed clinicians who provided a combination of Child‐Parent Psychotherapy and Mindful Parenting Education (Zarnegar et al., 2016)

One study evaluated parent training workshops designed to teach behavioural regulation skills to children with FASD but did not include any clear EF outcome measures (Kable et al., 2012). One clinical trial registry record could not be verified in terms of eligibility due to a lack of information available (Coles, 2005). One study included an evaluation of an eligible EF intervention and eligible outcome measures but used a sample which comprised a combination of children with FASD and Autism Spectrum Disorder (Kerns et al., 2017) and so was excluded from the review.

### 5.2 Risk of bias in included studies

#### 5.2.1 RCTs

**Figure 3:**
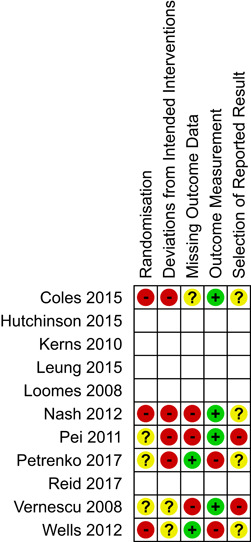
Risk of bias summary: review authors' judgements about each risk of bias item for included randomised controlled trials.

**Figure 4:**
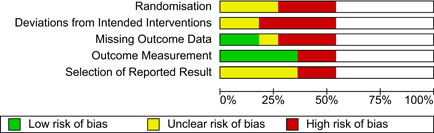
Risk of bias graph

All RCTs studies were rated as having an overall high risk of bias due to at least one of the five RoB2 domains being rated as ‘high’ (see Figures 3 and 4 and characteristics of included studies). Although labelled as RCTs, little to no information was reported on the randomisation sequence or concealment, and there was variable assessment of baseline differences. This meant that the studies were rated as having unclear or high risk of bias for the randomisation domain. In relation to the ‘Deviations from intended interventions’ domain, all studies were rated as either high or unclear risk of bias. As is often the case in applied psychological intervention research, participants and implementers are likely aware of the condition to which they have been assigned. There was only one report of deviations from the intended intervention (Petrenko et al., 2017), and most authors conducted appropriate analyses to estimate the effect of assignment to conditions (e.g., intention‐to‐treat vs. per‐protocol).

The ratings for the ‘Missing outcome data’ domain were mixed. Four studies (Coles et al., 2015; Nash, 2012; Pei et al., 2011; Vernescu, 2008) were rated as having high or unclear risk of bias due to either having <95% available data for participants and/or no assessment of the impact of missing data (likely due to small sample sizes). Studies inconsistently reported reasons for missing data, making it difficult to determine if the missing data was related to its true value in all cases. or whether it could be related to the true value of the outcome. Two studies were rated as having low risk of bias due to reporting data for all participants randomised (Petrenko et al., 2017; Wells et al., 2012).

In terms of the measurement of outcomes, all but two studies were rated as having low risk of bias due to the use of standardised direct assessments of EF, which are less likely to be influenced by knowledge of the intervention. However, studies inconsistently reported whether outcome assessors were blind to participant assignment. Two studies were rated as having high risk of bias as only indirect parent‐ or teacher‐report measures were used to assess EF and this type of outcome measure may be influenced by knowledge of the intervention (Petrenko et al., 2017; Wells et al., 2012).

All studies were rated as having either high or unclear risk of bias for the ‘Selection of the reported result’ domain, predominantly because no pre‐specified analysis plan or protocol could be located. Most studies included more than one measurement of an eligible outcome and tended to vary on the completeness of reporting, with some selective reporting based on statistical significance.

#### 5.2.2 Quasi‐experimental comparison group studies

The single eligible quasi‐experiment with a comparison group (Loomes et al., 2008) was rated as having an overall serious risk of bias due to a rating of serious for at least one of the domains for the ROBINS‐I tool. While there was baseline assessments of the outcome measures, other potential baseline confounders or time‐varying confounders were either not measured or accounted for in analyses (e.g., demographics, time of measurements between groups), leading to a rating of serious risk of bias for the confounding domain. Selection of study participants did not appear to be based on participant characteristics observed after the start of intervention (selection domain), suggesting that participants who would have been eligible were included in the study. However, it is unclear whether the start of follow‐up and start of the intervention coincide for participants in each group. As a result, this domain was rated as being at moderate risk of bias.

The intervention and comparison conditions were clearly articulated by study authors, presumably before the start of the intervention, leading to a rating of low risk of bias for the classification of interventions domain. There were also no reported deviations from the intended intervention, leading to a low risk of bias for this domain. Data for all recruited participants appeared to be included in analyses, also leading to a rating of low risk of bias for the missing data domain. Similar to the included RCTs, the measurement of outcomes domain was also rated as having low risk of bias because knowledge of assignment to conditions was unlikely to have affected the standardised direct measures of EF and because the methods of outcome assessment appeared comparable between the intervention and comparison groups. Finally, the selection of reported results domain was rated as having moderate risk of bias due to there being no prospectively published protocol or analysis plan for the study, however, there was only one measurement and analysis of the eligible outcome which was clearly reported in the study report.

### 5.3 Effects of interventions

#### 5.3.1 Direct measures of EF

##### 5.3.1.1 Attention (auditory)

Four RCT/quasi‐experimental studies with a comparison group reported direct measures of auditory attention (Coles et al., 2015; Nash, 2012; Pei et al., 2011; Vernescu, 2008). One study (Vernescu, 2008) reported two direct measures of auditory attention, which were combined using the approach detailed in Section 4.3.5. Another study reported measuring auditory attention via the NEPSY and one other potentially eligible measure of auditory attention (KiTAP, subtest unspecified), but no data was reported in any study reports (Pei et al., 2011). Efforts to obtain the required data from authors for effect size calculation were unsuccessful, so only three studies were meta‐analysed.

**Figure 5:**
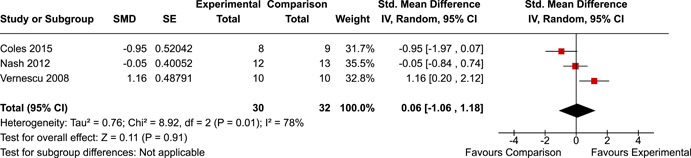
(Analysis 2.1) Forest plot for auditory attention (direct, ICC = 0.00)

**Figure 6:**
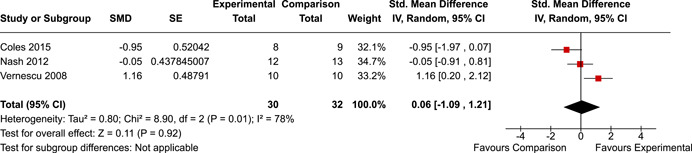
(Analysis 2.2) Forest plot for auditory attention (direct, ICC = 0.02)

**Figure 7:**
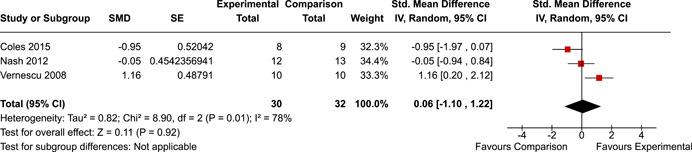
(Analysis 2.3) Forest plot for auditory attention (direct, ICC = 0.03)

**Figure 8:**
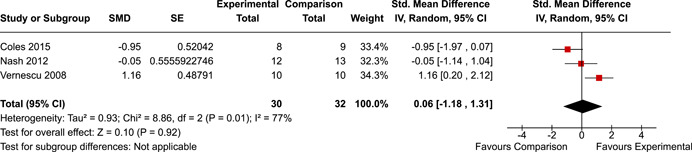
(Analysis 2.4) Forest plot for auditory attention (direct, ICC = 0.1)

The meta‐analysis included a total of 62 participants (experimental *n* = 30; comparison *n* = 32). The overall effect for auditory attention across studies was small in size and positive (SMD = 0.06, 95% CI = −1.06, 1.18), but not statistically significant. This indicates that participants receiving an EF‐focused psychological intervention did not differ from comparison participants on direct measures of auditory attention. A forest plot of the distribution of effect sizes is presented in Figure 5. Sensitivity analyses indicated that when the standard errors of the single study with clustering (Nash, 2012) were adjusted, the results did not change for an ICC of 0.02, 0.03 or 0.1 (see Figures 6, 7, 8). There was significant heterogeneity across studies (*τ*² = 0.76; *χ*² = 8.92, *df* = 2; *p* = 0.01; *I*² = 78%). However, the small number of studies precludes an accurate estimate of the variance component, *τ* ^2^. Potential sources of this heterogeneity (see Table 5) could be the format of the intervention, the use of different practitioners to implement the intervention or the developmental age of the child participants.

**Table 5:** Potential sources of heterogeneity across included studies included in meta‐analyses

Study	Design	Comparison	Measurement time‐points	Child age (years)	Intervention format	Duration and intensity	Intervention setting	Implementer	Diagnostic process
Coles et al. (2015)	RCT	No treatment	Baseline, post‐intervention (short)	Mean = 7–7.5	One‐on‐one F2F + computer Children and parents	5 weeks Weekly sessions	Unclear	Allied health and educational professionals	Institute of Medicine/Hoyme revision
Vernescu (2008)	RCT	TAU	Baseline, post‐intervention (short)	Mean = 9.5–9.54	One‐on‐one F2F Children only	2.5–3.5 weeks 12 × 30 min sessions	School	Teachers	Specific diagnostic framework not reported, but formal diagnosis by physician required for participant eligibility
Nash (2012)	RCT	Wait list control	Baseline, post‐intervention (short)	Mean = 10.3–10.5	One‐on‐one F2F Children only	12–14 weeks 12 × 60 min sessions	Hospital Clinic	Graduate clinical psychology students	Canadian Diagnostic Guidelines (*n* = 16) Washington 4‐digit (*n* = 9)
Petrenko et al. (2017)	RCT	TAU	Baseline, post‐intervention (medium/long) and follow‐up (medium)	Mean = 6.52–6.49	One‐on‐one + group F2F Children and parents	30‐weeks Weekly individual Fortnightly group	Family home	Graduate psychology students (clinical or educational)	FASD diagnosis using diagnostic guidelines (Revised Institute of Medicine, Hoyme et al., 2005) or confirmed PAE
Wells et al. (2012)	RCT	No treatment	Baseline, post‐intervention (short)	Mean = 8.12–9.24	Group for children, group for parents (concurrent), child‐parent practice component F2F	12‐weeks Weekly 75 min sessions	Unclear	Graduate‐level therapists (e.g., occupational therapists)	Met criteria for FAS, partial FAS or ARND

Abbreviations: FAS, foetal alcohol syndrome; FASD, foetal alcohol spectrum disorder; F2F, face‐to‐face; RCT, randomised controlled trial; TAU, treatment‐as‐usual.

Three single‐group pre‐post studies reported direct measures of auditory attention before and after an eligible intervention (Hutchison, 2015; Kerns et al., 2010; Leung et al., 2015). However, Leung et al. (2015) (*N* = 20) did not report post‐intervention data (NEPSY: Auditory Attention). Hutchison (2015) and Kerns et al. (2010) both reported Cohen's *d,* but no other data which could be used to combine the effect sizes with meta‐analysis (e.g., pre‐post means, SD, *p*‐value of *d* or confidence intervals)*.* Hutchison (2015) reported a Cohen's *d* of 0.83 (*N* = 9) and Kerns et al. (2010) reported Cohen's *d* of 1.26 (*N* = 10). All authors were contacted to obtain missing data and permit meta‐analysis, however, author contact details could not be located for Hutchison (2015) and Leung et al. (2015) could not provide the required data, and no response was received from Kerns et al. (2010). While both of these reported effects suggest more positive and larger effects than the two group experimental studies, they must be interpreted with caution due to the tendency for single‐group studies to over‐estimate true effects and high risk of bias.

##### 5.3.1.2 Attention (visual)

Three RCT/quasi‐experimental studies with a comparison group directly measured visual attention and reported this as an outcome (Coles et al., 2015; Pei et al., 2011; Vernescu, 2008). Vernescu (2008) measured visual attention directly using KiTAP Ghost's Ball (omission errors) and TEA‐CH Sky Search DT, however, the required data to calculate an effect size for TEA‐Ch Sky Search DT was not reported. Efforts to contact the author for this data were unsuccessful, so only one effect size—rather than an aggregated effect size of the dependent outcomes—was used in the meta‐analysis. Pei et al. (2011) reported measuring visual attention and two other potentially eligible measures of auditory attention (KiTAP, subtest unspecified; Farm Animals Game), but no data was reported in any study reports. While Coles et al. (2015) directly measured various aspects of attention using the TOVA, the errors of omission scale is considered the most direct assessment of visual attention and so was used for effect size calculation to calculate an effect size for visual attention (see Table 1). Efforts to obtain the required data from authors for effect size calculation were unsuccessful, so only two studies were meta‐analysed.

**Figure 9:**
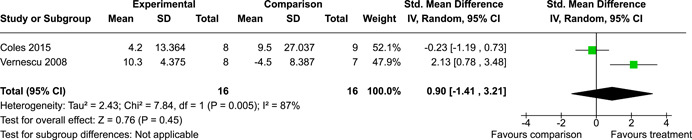
(Analysis 1.1) Forest plot for visual attention (direct)

The meta‐analysis included a total of 32 participants (experimental *n* = 16; comparison *n* = 16) and the overall effect for visual attention across studies was medium in size and positive, but not statistically significant (SMD = 0.90, 95% CI = −1.41, 3.21). This indicates that participants receiving an EF‐focused psychological intervention did not differ from comparison participants on direct measures of visual attention. A forest plot of the distribution of effect sizes is presented in Figure 9. There was significant heterogeneity across the two studies (*τ* ^2^ = 2.43; *χ* ^2^ = 7.84, *df* = 1, *p* = 0.005; *I* ^2^ = 87%). However, the small number of studies precludes an accurate estimate of the variance component, *τ* ^2^. Potential sources of this heterogeneity (see Table 5) could be the format of the intervention, the use of different practitioners to implement the intervention or the presentation format for the test used to measure the outcome (e.g., KiTAP may be more interesting for children when engaging in the task).

Two single‐group pre‐post studies reported a direct measure of visual attention before and after an eligible intervention (Kerns et al., 2010; Leung et al., 2015). While Kerns et al. (2010) reported measuring KiTAP Ghost's Ball (omissions), the data was not reported by the authors and attempts to locate the missing data via contact with the authors were not successful. Leung et al. (2015) reported a nonsignificant increase in visual attention between baseline (*N* = 20; *M* = 54.79, SD = 21.04) and posttreatment (*M* = 65.11; SD = 22.92), with a standardised mean gain score of 0.25 (SE = 0.125). Leung et al. (2015) did not report data for the direct measure of visual attention at the follow‐up period (5 weeks after intervention completion). However, visual inspection of the figures in the associated thesis report suggests that the direct measure of visual attention (TOVA omissions) reduced to almost baseline levels.

##### 5.3.1.3 Cognitive flexibility

**Figure 10:**
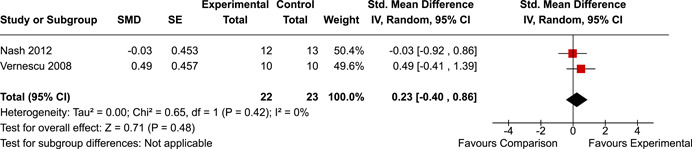
(Analysis 3.1) Forest plot for cognitive flexibility (direct)

**Figure 11:**
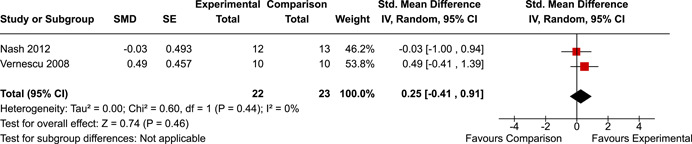
(Analysis 3.2) Forest plot (3.2 Cognitive flexibility [direct, ICC = 0.02])

**Figure 12:**
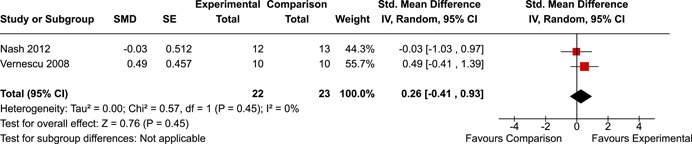
(Analysis 3.3) Forest plot (3.3 Cognitive flexibility [direct, ICC = 0.03])

**Figure 13:**
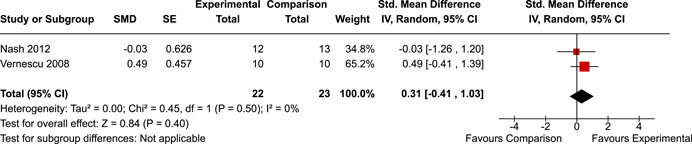
(Analysis 3.4) Forest plot (3.4 Cognitive flexibility [direct, ICC = 0.1])

Two RCT/quasi‐experimental studies with a comparison group reported direct measures of cognitive flexibility as an outcome (Nash, 2012; Vernescu, 2008), with Nash (2012) reporting two direct measures of cognitive flexibility. The two direct measures of cognitive flexibility reported in Nash (2012) were combined before meta‐analysis using the approach specified in the Unit of Analysis section. The meta‐analysis included a total of 45 participants (experimental *n* = 22; comparison *n* = 23). The overall effect for cognitive flexibility across studies was positive (SMD = 0.23, 95% CI = −0.40, 0.86), but small and not statistically significant. This indicates that participants receiving an EF‐focused psychological intervention did not differ from comparison participants on direct measures of cognitive flexibility. A forest plot of the distribution of effect sizes is presented in Figure 10. There was no significant heterogeneity across studies: *τ*² = 0.00; *χ*² = 0.65, *df* = 1, *p* = 0.42; *I*² = 0%. However, the small number of studies precludes an accurate estimate of the variance component, *τ* ^2^. Sensitivity analyses indicated that when the standard errors of the single study with clustering (Nash, 2012) were adjusted, the results did not change for an ICC of 0.02, 0.03 or 0.1 (see Figures 11, 12, 13).

Four single‐group pre‐post studies reported direct measures of cognitive flexibility before and after an eligible intervention (*N* = 41; Hutchison, 2015; Kerns et al., 2010; Leung et al., 2015; Reid et al., 2017). However, Leung et al. (2015) (*N* = 20) did not report post‐intervention data to allow calculation of an effect size (NEPSY: inhibition/switching (switching subtest); NEPSY: response set). Hutchison (2015) and Kerns et al. (2010) both reported Cohen's *d,* but no other data which could be used to combine the effect sizes with meta‐analysis (e.g., pre‐post means, SD, *p*‐value of *d* or confidence intervals)*.* Hutchison (2015) (*N* = 9) reported a Cohen's *d* of 0.98, 0.17 and 0.32 across the three direct measures of cognitive flexibility (NEPSY: inhibition/switching (switching subtest); NEPSY response set combined; NIH: dimensional card sort respectively). Kerns et al. (2010) (*N* = 10) reported a Cohen's *d* of 0.58 for KiTAP (owls). All authors were contacted to obtain missing data and permit meta‐analysis, however, author contact details could not be located for Hutchison (2015), Leung et al. (2015) could not provide the required data, and no response was received from Kerns et al. (2010). Reid et al. (2017) reported individual participant data using the NEPSY: inhibition/switching subtest (switching errors) at three time‐points for two children: baseline (Child 1 errors = 16; Child 2 errors = 3); post‐intervention (Child 1 errors = 16; Child 2 errors = 1); follow‐up (Child 1 errors = 7; Child 2 errors = 0). While these reported results suggest larger effects than the two group experimental studies, they must be interpreted with caution due to the tendency for single‐group studies to over‐estimate true effects and their high risk of bias.

##### 5.3.1.4 Inhibition (attentional)

**Figure 14:**
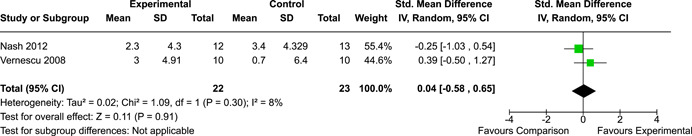
(Analysis 4.1) Forest plot (4.1 Attentional inhibition [direct, ICC = 0.00])

**Figure 15:**
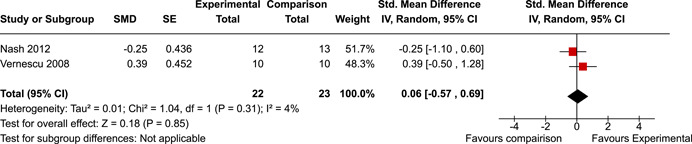
(Analysis 4.2) Forest plot (4.2 Attentional inhibition [direct, ICC = 0.02])

**Figure 16:**
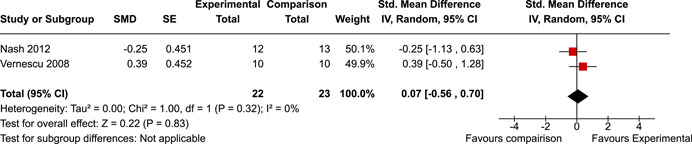
(Analysis 4.3) Forest plot (4.3 Attentional inhibition [direct, ICC = 0.03])

**Figure 17:**
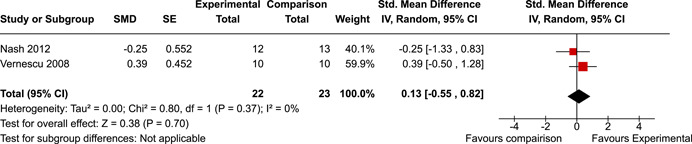
(Analysis 4.4) Forest plot (4.4 Attentional inhibition [direct, ICC = 0.1])

Two RCT/quasi‐experimental studies with a comparison group reported direct measures of attentional inhibition as an outcome. A third study potentially measured attentional inhibition, however the distinction between attentional and response inhibitions was not possible based on the description of measures in available reports of the study (Pei et al., 2011). In addition, Pei et al. (2011) reported no data that could be used to calculate effect sizes and attempts to obtain the data were unsuccessful. As such, this study was omitted from meta‐analyses. Vernescu (2008) measured attentional inhibition directly using TEA‐Ch Map Mission and Sky Search (attention score), however, the required data to calculate an effect size for TEA‐Ch Sky Search was not reported. Efforts to contact the author for this data were unsuccessful, so only one effect size—rather than an aggregated effect size of the dependent outcomes—was used in the meta‐analysis. The meta‐analysis included a total of 45 participants (experimental *n* = 22; comparison *n* = 23). The overall effect for attentional inhibition across studies was positive (SMD = 0.04, 95% CI = −0.58, 0.65), but small and not statistically significant. This indicates that participants receiving an EF‐focused psychological intervention did not differ from comparison participants on direct measures of attentional inhibition. A forest plot of the distribution of effect sizes is presented in Figure 14. There was no significant heterogeneity across studies: *τ*² = 0.02; *χ*² = 1.09, *df* = 1, *p* = 0.30; *I*² = 8%. However, the small number of studies precludes an accurate estimate of the variance component, *τ* ^2^. Sensitivity analyses indicated that when the standard errors of the single study with clustering (Nash, 2012) were adjusted, the overall effect size slightly increased, but the confidence intervals still included zero (see Figures 15, 16, 17).

One single‐group study reported a direct measure of attentional inhibition as an outcome before and after an eligible intervention (*N* = 10; Kerns et al., 2010). Kerns et al. (2010) reported Cohen's *d* of 1.07, but no confidence interval or standard error, or data that could be used to calculate these by hand. The lead author was contacted for the missing data, but no response was received. While this suggests a larger effect of the intervention than studies that included a comparison group, the effect must be interpreted with caution due to the tendency for single‐group studies to over‐estimate true effects and their high risk of bias.

##### 5.3.1.5 Inhibition (response)

Three RCT/quasi‐experimental studies with a comparison group reported direct measures of response inhibition as an outcome (Coles et al., 2015; Nash, 2012; Vernescu, 2008). A fourth study potentially measured attentional inhibition, however the distinction between attentional and response inhibitions was not possible based on the description of measures in available reports of the study (Pei et al., 2011). In addition, Pei et al. (2011) reported no data that could be used to calculate effect sizes and attempts to obtain the data were unsuccessful. As such, this study was omitted from meta‐analyses. Coles et al. (2015) reported two direct measures of response inhibition and Vernescu (2008) reported four direct measures of response inhibition, but post‐intervention data for only two of the measures (only significant results were reported).

**Figure 18:**
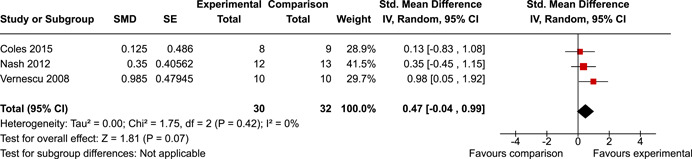
(Analysis 5.1) Forest plot (5.1 Response inhibition [direct, ICC = 0.00])

**Figure 19:**
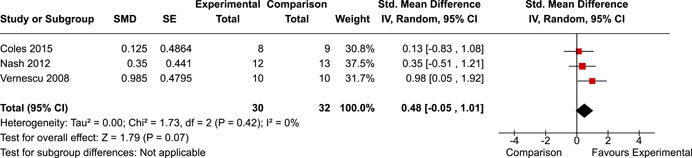
(Analysis 5.2) Forest plot (5.2 Response inhibition [direct, ICC = 0.02])

**Figure 20:**
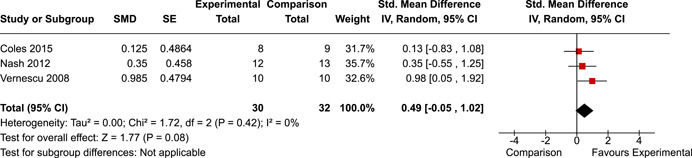
(Analysis 5.3) Forest plot (5.3 Response inhibition [direct, ICC = 0.03])

**Figure 21:**
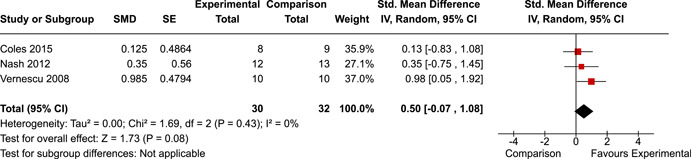
(Analysis 5.4) Forest plot (5.4 Response inhibition [direct, ICC = 0.1])

For outcomes with sufficient data, the effects sizes were combined within each study before meta‐analysis using the approach specified in Section 4.3.5. The meta‐analysis included a total of 62 participants (experimental *n* = 30; comparison *n* = 32). The overall effect for response inhibition across studies was positive, medium‐sized, but not statistically significant (SMD = 0.47, 95% CI = −0.04, 0.99). This indicates that participants receiving an EF‐focused psychological intervention did not differ from comparison participants on direct measures of response inhibition. A forest plot of the distribution of effect sizes is presented in Figure 18. Sensitivity analyses indicated that when the standard errors of the single study with clustering (Nash, 2012) were adjusted, the overall effect size slightly increased, with confidence intervals slightly increasing with the ICC value (see Figures 19, 20, 21). There was no significant heterogeneity across studies (*τ* ^2^ = 0.00; *χ* ^2^ = 1.75, *df* = 2, *p* = 0.42; *I* ^2^ = 0%).

Four single‐group pre‐post studies reported direct measures of response inhibition before and after an eligible intervention (Hutchison, 2015; Kerns et al., 2010; Leung et al., 2015; Reid et al., 2017). Hutchison (2015) and Kerns et al. (2010) both reported Cohen's *d,* but no other data which could be used to combine the effect sizes with meta‐analysis (e.g., pre‐post means, SD, *p*‐value of *d* or confidence intervals)*.* The authors were contacted to obtain missing data and permit meta‐analysis, however, author contact details could not be located for Hutchison (2015) and no response was received from Kerns et al. (2010).

Hutchison (2015) (*N* = 9) reported Cohen's *d*s of 0.6 and 0.32 for two different direct measures of response inhibition. Kerns et al. (2010) (*N* = 10) reported Cohen's *ds* of 1.07, 1.00, 0.58 and 0.3 across four measures of response inhibition. Leung et al. (2015) (*N* = 20) used two direct measures of response inhibition: TOVA (commissions) and NEPSY (Inhibition) but only reported post‐intervention data for the TOVA. Leung et al. (2015) reported a significant increase in response inhibition between baseline (*M* = 66.95; SD = 23.84) and posttreatment (*M* = 78.74; SD = 27.18), with a standardised mean gain score of 0.24 (SE = 0.124). Leung et al. (2015) did not report data for the direct measure of response inhibition at the follow‐up period (5 weeks after intervention completion). However, visual inspection of the figures in the associated thesis report suggests that the direct measure of visual attention (TOVA commissions) showed continued improvement over time. Reid et al. (2017) reported individual participant data using the NEPSY: Inhibition subtest at three time‐points for two children: baseline (Child 1 errors = 12; Child 2 errors = 2); post‐intervention (Child 1 errors = 19; Child 2 errors = 0); follow‐up (Child 1 errors = 3; Child 2 errors = 0). While these reported results suggest similar effects to the experimental studies with comparison groups, they must be interpreted with caution due to the tendency for single‐group studies to over‐estimate true effects and their high risk of bias.

##### 5.3.1.6 Planning

One of the RCT/quasi‐experimental studies with a comparison group reported a direct measure of planning as an outcome. Nash (2012) included a total of 25 participants (experimental *n* = 12; comparison *n* = 13) and the effect of the intervention was positive (SMD = 0.17, 95% CI = −0.61, 0.96) and not statistically significant. This indicates that participants receiving the psychological EF‐focused intervention did not significantly differ from comparison participants on the direct measure of planning.

No single‐group studies included any direct measures of planning.

##### 5.3.1.7 Working memory (verbal)

Two RCT/quasi‐experimental studies with a comparison group reported a direct measure of verbal working memory as an outcome, yet both reported insufficient data to calculate an effect size using RevMan analysis tools (Loomes et al., 2008; Pei et al., 2011). Efforts to obtain missing data from the study authors was unsuccessful. Loomes et al. (2008) reported sufficient data to enable calculation of a Cohen's *d* using the *t*‐value reported for the between group effect after the intervention. This effect size was calculated using David B. Wilson's suite of effect size calculators (campbellcollaboration.org/escalc/html/EffectSizeCalculator-SMD2.php). The effect size calculated was moderate and favoured the experimental group participants (*d* = 0.6827; 95% CI = −0.0196, 1.385) but was not statistically significant.

Three single‐group pre‐post studies reported direct measures verbal working memory as an outcome before and after an eligible intervention (*N* = 39; Hutchison, 2015; Kerns et al., 2010; Leung et al., 2015). Hutchison (2015) and Kerns et al. (2010) both reported Cohen's *d,* but no other data which could be used to combine the effect sizes with meta‐analysis (e.g., pre‐post means, SD, *p*‐value of *d* or confidence intervals)*.* Hutchison (2015) reported a Cohen's *d* of 0.15 for a direct measure of verbal working memory (WISC Digit Span, Backwards). Kerns et al. (2010) reported a Cohen's *d* of 0.37 a direct measure of verbal working memory (children's size ordering task). Leung et al. (2015) reported a significant increase in verbal working memory between baseline (*M* = 93.25, SD = 14.10) and posttreatment (*M* = 99.41; SD = 16.00), with a standardised mean gain score of 0.32 (SE = 0.183). Leung et al. (2015) did not report data for the direct measure of verbal working memory at the follow‐up period (5 weeks after intervention completion). However, visual inspection of the figures in the associated thesis report suggests that the direct measure of verbal working memory continued to increase, albeit at a slower rate than between baseline and post‐intervention. While these studies collectively suggest variable sized effects that somewhat align with the meta‐analysis of the two group experimental studies, the effect sizes must be interpreted with caution due to the tendency for single‐group studies to over‐estimate true effects and their high risk of bias.

##### 5.3.1.8 Working memory (visual)

One of the RCT/quasi‐experimental studies with a comparison group reported a direct measure of visual working memory as an outcome, yet reported insufficient data to calculate an effect size (Pei et al., 2011). Efforts to obtain the missing data from study authors were unsuccessful.

Three single‐group pre‐post studies reported direct measures of visual working memory before and after an eligible intervention. However, Leung et al. (2015) did not report post‐intervention data to allow calculation of effect sizes. Hutchison (2015) and Kerns et al. (2010) both reported Cohen's *d,* but no other data which could be used to combine the effect sizes with meta‐analysis (e.g., pre‐post means, SD, *p*‐value of *d* or confidence intervals)*.* Hutchison (2015) (*N* = 9) reported a Cohen's *d* of 0.15 and 0.14 across two direct measures of visual working memory (WISC spatial span, backwards and NIH list sorting, respectively). Kerns et al. (2010) (*N* = 10) reported a Cohen's *d* of 0.63 for a direct measure of verbal working memory (WISC spatial span, backwards). While these studies collectively suggest small to medium‐sized effects, they must be interpreted with caution due to the tendency for single‐group studies to over‐estimate true effects and their high risk of bias.

#### 5.3.2 Indirect measures of EF

Most eligible indirect outcome measures were derived from the BRIEF, which is a questionnaire designed to measure caregiver or teacher perceptions of behaviours in children which reflect various EFs. The measure is comprised of nine subscales (inhibit, self‐monitoring, shifting, emotional control, initiate, working memory, plan/organise, task‐monitoring and organisation of materials) and three composite scales that combine the subscales (Global Executive Composite [GEC], Behavioural Regulation Index and Meta‐cognitive Index). Other similar indirect measures of executive functioning follow a similar approach (e.g., Child Behaviour Questionnaire [CBQ]). Based on the structure of these measures, the sections below begin with a synthesis of composite measures of EF, followed by a synthesis of specific EF subscales from the indirect measures.

##### 5.3.2.1 Composite measures of EF

###### 5.3.2.1.1 Global Executive Composites (GEC)

**Figure 22:**
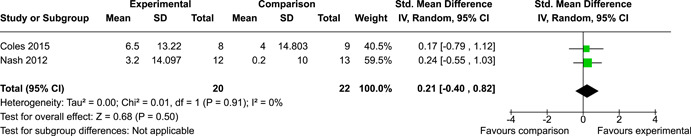
(Analysis 7.1) Forest plot (7.1 BRIEF—global Executive Composite)

**Figure 23:**
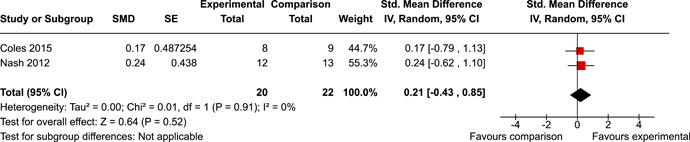
(Analysis 7.2) Forest plot (7.2 Global Executive Composite [indirect, ICC = 0.02])

**Figure 24:**
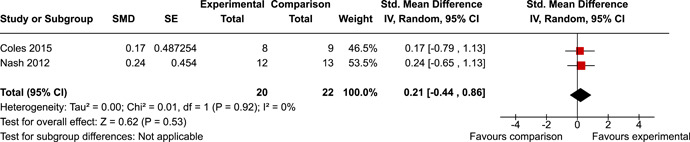
(Analysis 7.3) Forest plot (7.3 Global Executive Composite [indirect, ICC = 0.03])

**Figure 25:**
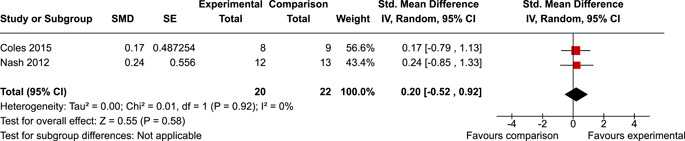
(Analysis 7.4) Forest plot (7.4 Global Executive Composite [indirect, ICC = 0.1])

Three RCT/quasi‐experimental studies with a comparison group used the GEC from the BRIEF as an indirect measure of global EF (Coles et al., 2015; Nash, 2012; Pei et al., 2011), with one reporting insufficient data to calculate an effect size (Pei et al., 2011). Efforts to obtain the missing data from study authors were unsuccessful. The GEC composite is comprised of all eight of the BRIEF subscales, each of which represents a different component of executive functioning. The meta‐analysis of two studies with available data included a total of 42 participants (experimental *n* = 20; comparison *n* = 22). The overall effect for global EF across studies was positive (SMD = 0.21, 95% CI = −0.40, 0.82) but not statistically significant. This indicates that participants receiving an EF‐focused psychological intervention did not differ from comparison participants on indirect measures of global EF. A forest plot of the distribution of effect sizes is presented in Figure 22. There was no significant heterogeneity across studies (*τ* ^2^ = 0.00; *χ* ^2^ = 0.01, *df* = 1, *p* = 0.91; *I* ^2^ = 0%). Sensitivity analyses indicated that when the standard errors of the single study with clustering (Nash, 2012) were adjusted, the results did not change for an ICC of 0.02, 0.03 or 0.1 (see Figures 23, 24, 25).

Three single‐group pre‐post studies reported indirect measures of global EF before and after an eligible intervention (Hutchison, 2015; Leung et al., 2015; Reid et al., 2017). Hutchison (2015) and Leung et al. (2015) had missing data, which precluded combining the studies with meta‐analysis. Authors of both studies were contacted to obtain missing data, however, author contact details could not be located for Hutchison (2015). Leung et al. (2015) (*N* = 9) reported a Cohen's *d,* but no other data which could be used to combine the effect sizes with meta‐analysis (e.g., pre‐post means, SD, *p*‐value of *d* or confidence intervals)*.* Leung et al. (2015) (*N* = 20) did not report post‐intervention data to allow calculation of effect sizes for either measure of global EF (BRIEF: GEC; Conner's EF composite).

Hutchison (2015) (*N* = 9) reported a large Cohen's *d* of 0.925 for the teacher‐report BRIEF. Reid et al. (2017) reported individual participant data using the parent‐report BRIEF, with one report provided by each parent for each child at three time‐points for two children: baseline (Child 1 GEC = 67/74; Child 2 GEC = 69/76); post‐intervention (Child 1 GEC = 64/62; Child 2 GEC = 67/75); follow‐up (Child 1 GEC = 58/62; Child 2 GEC = 63/73). Reliable Change Index scores reported by the authors suggest that (a) Child 1 ‘recovered’ when using one parent's report and ‘improved’ for the other parent; and (b) Child 2's GEC was ‘unchanged’ across both parents. While these reported results suggest larger effects than the two group experimental studies, the effects must be interpreted with caution due to the tendency for single‐group studies to over‐estimate true effects and their high risk of bias.

###### 5.3.2.1.2 Effortful control (CBQ)

One RCT/quasi‐experimental study with a comparison group reported an indirect measure of effortful control as an outcome. This is a composite measure of four subscales from the CBQ: attentional focusing, inhibitory control, low‐intensity pleasure and perceptual sensitivity. Coles et al. (2015) included a total of 17 participants (experimental *n* = 8; comparison *n* = 9) and the effect of the intervention was negative (SMD = −0.53, 95% CI = −1.50, 0.45). This indicates that participants receiving the psychological EF‐focused intervention did not significantly differ from comparison participants on the indirect measure of effortful control.

No single‐group studies included any indirect measures of effortful control.

###### 5.3.2.1.3 Behavioural regulation (BRIEF, Behavioural Regulation Index)

**Figure 26:**
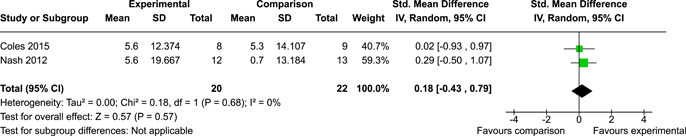
(Analysis 9.1) Forest plot (9.1 Behavioural regulation [indirect])

**Figure 27:**
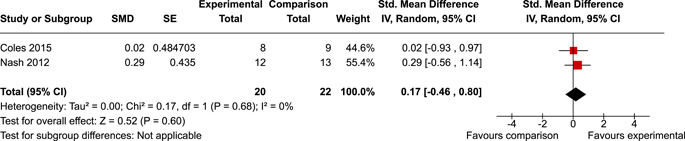
(Analysis 9.2) Forest plot (9.2 Behavioural regulation [indirect, ICC = 0.02])

**Figure 28:**
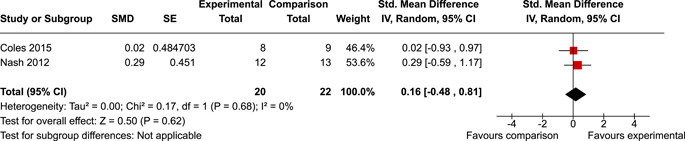
(Analysis 9.3) Forest plot (9.3 Behavioural regulation [indirect, ICC = 0.03])

**Figure 29:**
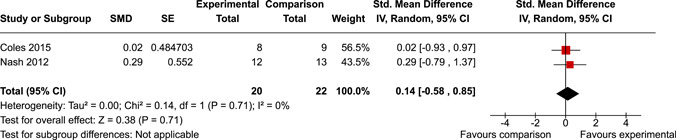
(Analysis 9.4) Forest plot (9.4 Behavioural regulation [indirect, ICC = 0.1])

Two RCT/quasi‐experimental studies with a comparison group used the BRIEF BRI as an indirect measure of EF (Coles et al., 2015; Nash, 2012). The meta‐analysis included a total of 42 participants (experimental *n* = 20; comparison *n* = 22) and the overall effect was positive (SMD = 0.18, 95% CI = −0.43, 0.79) but not statistically significant. This indicates that participants receiving an EF‐focused psychological intervention did not differ from comparison participants on indirect measures of global behavioural regulation. A forest plot of the distribution of effect sizes is presented in Figure 26. There was no significant heterogeneity across studies (*τ* ^2^ = 0.00; *χ* ^2^ = 0.18, *df* = 1, *p* = 0.68; *I* ^2^ = 0%). Sensitivity analyses indicated that when the standard errors of the single study with clustering (Nash, 2012) were adjusted, the results did not change for an ICC of 0.02, 0.03 or 0.1 (see Figures 27, 28, 29).

Three single‐group pre‐post studies reported an indirect measure of behavioural regulation using the BRIEF BRI composite before and after an eligible intervention (Hutchison, 2015; Leung et al., 2015; Reid et al., 2017). Hutchison (2015) and Leung et al. (2015) had missing data, which precluded combining the studies with meta‐analysis. Authors of both studies were contacted to obtain missing data, however, author contact details could not be located for Hutchison (2015). Leung et al. (2015) (*N* = 9) reported a Cohen's *d,* but no other data which could be used to allow effect sizes combine the effect sizes with meta‐analysis (e.g., pre‐post means, SD, *p*‐value of *d* or confidence intervals)*.* Leung et al. (2015) (*N* = 20) did not report post‐intervention data to allow calculation of an effect size.

Hutchison (2015) (*N* = 9) reported a small Cohen's *d* of 0.267 for the teacher‐report BRIEF. Reid et al. (2017) reported individual participant data using the parent‐report BRIEF, with one report provided by each parent for each child at three time‐points for two children: baseline (Child 1 BRI = 62/64; Child 2 BRI = 73/74); post‐intervention (Child 1 BRI = 57/52; Child 2 BRI = 74/81); follow‐up (Child 1 BRI = 57/51; Child 2 BRI = 67/79). Reliable Change Index scores reported by the authors suggest that (a) Child 1 was ‘unchanged’ when using one parent's report and ‘recovered’ for the other parent; and (b) Child 2's BRI was ‘unchanged’ across both parents. While the reported results from single‐group studies suggest larger effects than the two group experimental studies, the effects must be interpreted with caution due to the tendency for single‐group studies to over‐estimate true effects and their high risk of bias.

###### 5.3.2.1.4 Meta‐cognition (BRIEF, Meta‐Cognition Index)

One RCT/quasi‐experimental study with a comparison group reported an indirect measure of meta‐cognition as an outcome. Coles et al. (2015) included a total of 17 participants (experimental *n* = 8; comparison *n* = 9) and the effect of the intervention was positive (SMD = 0.23, 95% CI = −0.72, 1.19), but not statistically significant. This indicates participants receiving the psychological EF‐focused intervention did not significantly differ from comparison participants on the indirect measure of inhibition.

Three single‐group pre‐post studies reported an indirect measure of meta‐cognition using the BRIEF MI composite before and after an eligible intervention (Hutchison, 2015; Leung et al., 2015; Reid et al., 2017). Hutchison (2015) and Leung et al. (2015) had missing data, which precluded combining the studies with meta‐analysis. Authors of both studies were contacted to obtain missing data, however, author contact details could not be located for Hutchison (2015). Leung et al. (2015) (*N* = 9) reported a Cohen's *d,* but no other data which could be used to combine the effect sizes with meta‐analysis (e.g., pre‐post means, SD, *p*‐value of *d* or confidence intervals)*.* Leung et al. (2015) (*N* = 20) did not report post‐intervention data to allow calculation of an effect size.

Hutchison (2015) (*N* = 9) reported a large Cohen's *d* of 1.6 for the teacher‐report BRIEF. Reid et al. (2017) reported individual participant data using the parent‐report BRIEF, with one report provided by each parent for each child at three time‐points for two children: baseline (Child 1 MI = 68/78; Child 2 MI = 65/69); post‐intervention (Child 1 MI = 66/66; Child 2 MI = 61/70); follow‐up (Child 1 MI = 58/69; Child 2 MI = 94/68). Reliable Change Index scores reported by the authors suggest that (a) Child 1 ‘recovered’ when using one parent's report and ‘improved’ for the other parent; and (b) Child 2's BRI was ‘unchanged’ across both parents. While the reported results from single‐group studies suggest larger effects than the two group experimental studies, the effects must be interpreted with caution due to the tendency for single‐group studies to over‐estimate true effects and their high risk of bias.

##### 5.3.2.2 Attention

One RCT/quasi‐experimental study with a comparison group reported an indirect measure of attention as an outcome (Pei et al., 2011) but reported insufficient data to calculate an effect size for either indirect measure of attention (Conner's; Attention Deficit Disorders Evaluation Scale). One single‐group pre‐post study reported an indirect measure of attention before and after an eligible intervention (Leung et al., 2015) and also did not report post‐intervention data to allow calculation of an effect size for either indirect measure of attention (Conner's; BASC‐2).

##### 5.3.2.3 Emotional control

**Figure 30:**
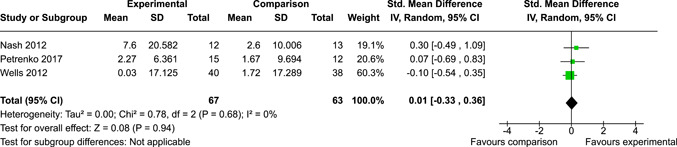
(Analysis 11.1) Forest plot (11.1 Emotional control [indirect])

**Figure 31:**
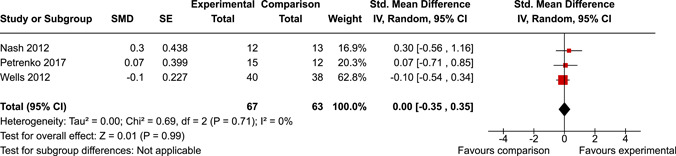
(Analysis 11.2) Forest plot (11.2 Emotional control [indirect, ICC = 0.02])

**Figure 32:**
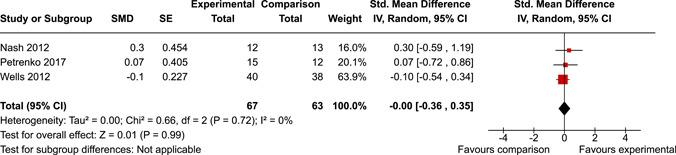
(Analysis 11.3) Forest plot (11.3 Emotional control [indirect, ICC = 0.03])

**Figure 33:**
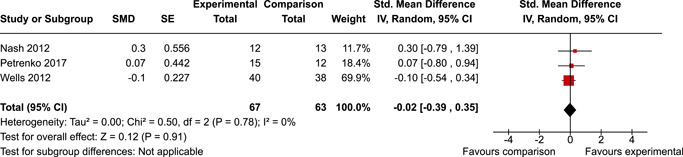
(Analysis 11.4) Forest plot (11.4 Emotional control [indirect, ICC = 0.1])

Three RCT/quasi‐experimental studies with a comparison group reported an indirect measure of emotional control as an outcome (Nash, 2012; Petrenko et al., 2017; Wells et al., 2012). The meta‐analysis included a total of 130 participants (experimental *n* = 67; comparison *n* = 63). The overall effect for emotional control across studies was small and positive (SMD = 0.01, 95% CI = −0.33, 0.36) but not statistically significant. This indicates that participants receiving psychological EF‐focused interventions did not significantly differ from comparison participants on indirect measures of emotional control. A forest plot of the distribution of effect sizes is presented in Figure 30. There was no significant heterogeneity across studies (*χ* ^2^ = 0.78, *df* = 2, *p* = 0.68; *I* ^2^ = 0%; *τ* ^2^ = 0.00). Sensitivity analyses indicated that when the standard errors of the two studies with clustering (Nash, 2012; Petrenko et al., 2017) were adjusted, the overall effect size to zero for an ICC of 0.02, 0.03 or 0.1 (see Figures 31, 32, 33).

Petrenko et al. (2017) conducted a 6‐month follow‐up with study participants (experimental *n* = 14; comparison *n* = 10), which indicated that the emotional control in both groups remained mostly stable. The effect size for their indirect measure of emotional control between the post‐intervention and follow‐up measurement continued to favour the experimental group, but was small in size and not statistically significant (SMD = 0.23, 95% CI = −0.59, 1.04).

No single‐group studies included any indirect measures of emotional control.

##### 5.3.2.4 Inhibition

**Figure 34:**
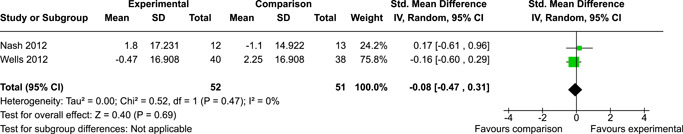
(Analysis 12.1) Forest plot (12.1 Inhibition [indirect])

**Figure 35:**
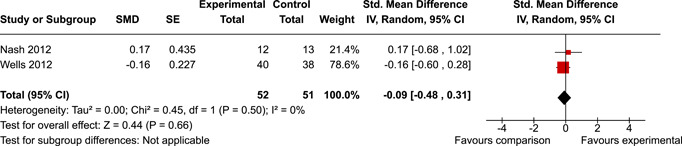
(Analysis 12.2) Forest plot (12.2 Inhibition [indirect, ICC = 0.02])

**Figure 36:**
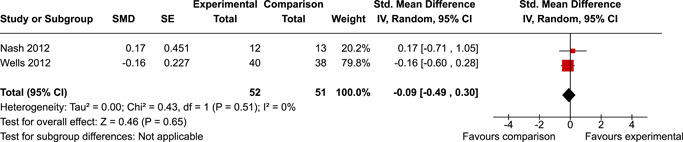
(Analysis 12.3) Forest plot (12.3 Inhibition [indirect, ICC = 0.03])

**Figure 37:**
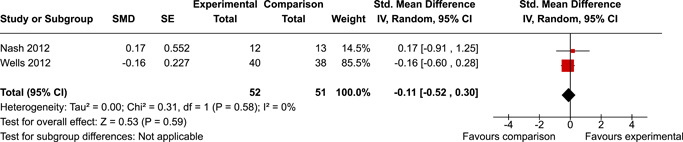
(Analysis 12.4) Forest plot (12.4 Inhibition [indirect, ICC = 0.1])

Two RCT/quasi‐experimental studies with a comparison group reported an indirect measure of inhibition as an outcome. The meta‐analysis included a total of 103 participants (experimental *n* = 52; comparison *n* = 51). The overall effect for inhibition across studies was negative (SMD = −0.08, 95% CI = −0.47, 0.31) but not statistically significant. This indicates that participants receiving an EF‐focused intervention did not significantly differ from comparison participants on indirect measures of inhibition. A forest plot of the distribution of effect sizes is presented in Figure 34. There was no significant heterogeneity across studies (*χ* ^2^ = 0.52, *df* = 1, *p* = 0.47; *I* ^2^ = 0%; *τ* ^2^ = 0.00). Sensitivity analyses indicated that when the standard errors of one study with clustering (Nash, 2012) were adjusted, the overall effect size to zero for an ICC of 0.02, 0.03 or 0.1 (see Figures 35, 36, 37).

One single‐group pre‐post study reported an indirect measure of inhibition before and after an eligible intervention. Hutchison (2015) (*N* = 9) reported a small and positive Cohen's *d* of 0.168 for the teacher‐report BRIEF. While the reported results from this single‐group study differs from the synthesis of the two group experimental studies, the effect must be interpreted with caution due to the tendency for single‐group studies to over‐estimate true effects and their high risk of bias.

##### 5.3.2.5 Shifting

**Figure 38:**
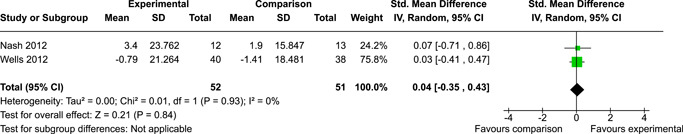
(Analysis 13.1) Forest plot (13.1 Shifting [indirect])

**Figure 39:**
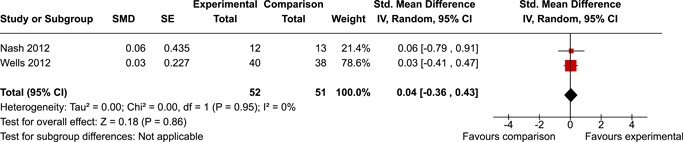
(Analysis 13.2) Forest plot (13.2 Shifting [indirect, ICC = 0.02])

**Figure 40:**
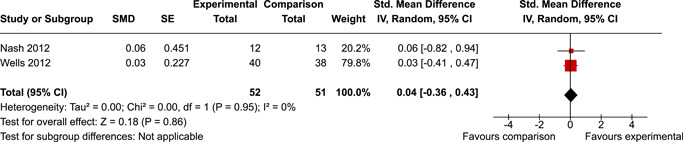
(Analysis 13.3) Forest plot (13.3 Shifting [indirect, ICC = 0.03])

**Figure 41:**
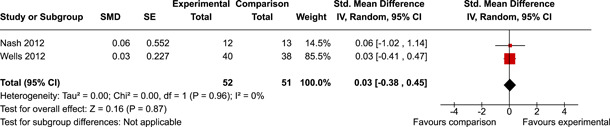
(Analysis 13.4) Forest plot (13.4 Shifting [indirect, ICC = 0.1])

Two RCT/quasi‐experimental studies with a comparison group reported an indirect measure of shifting as an outcome (Nash, 2012; Wells et al., 2012). The meta‐analysis included a total of 103 participants (experimental *n* = 52; comparison *n* = 51) and the overall effect for shifting was positive (SMD = 0.04, 95% CI = −0.35, 0.43) but not statistically significant. This indicates that participants receiving an EF‐focused intervention did not significantly differ from comparison participants on indirect measures of shifting. A forest plot of the distribution of effect sizes is presented in Figure 38. There was no significant heterogeneity across studies (*χ* ^2^ = 0.01, *df* = 1, *p* = 0.93; *I* ^2^ = 0%; *τ* ^2^ = 0.00). Sensitivity analyses indicated that when the standard errors of one study with clustering (Nash, 2012) were adjusted, the overall effect size to zero for an ICC of 0.02, 0.03 or 0.1 (see Figures 39, 40, 41).

One single‐group pre‐post study reported an indirect measure of shifting before and after an eligible intervention. Hutchison (2015) (*N* = 9) reported a medium and positive Cohen's *d* of 0.55 for the teacher‐report BRIEF. While the reported results from this single‐group study suggests larger effects than the synthesis of the two group experimental studies, the effect must be interpreted with caution due to the tendency for single‐group studies to over‐estimate true effects and their high risk of bias.

##### 5.3.2.6 Working memory

One of the RCT/quasi‐experimental studies with a comparison group reported an indirect measure of working memory as an outcome. Wells et al. (2012) included a total of 78 participants (experimental *n* = 40; comparison *n* = 38) and the effect of the intervention was negligible (SMD = 0.00, 95% CI = −0.45, 0.44). This indicates participants receiving the psychological EF‐focused intervention did not differ from comparison participants on the indirect measure of working memory.

One single‐group pre‐post study reported an indirect measure of working memory before and after an eligible intervention. Hutchison (2015) (*N* = 9) reported a large and positive Cohen's *d* of 1.01 for the teacher‐report BRIEF. While the reported results from this single‐group study suggests larger effects than the two group experimental study, the effect must be interpreted with caution due to the tendency for single‐group studies to over‐estimate true effects and their high risk of bias.

##### 5.3.2.7 Other singular BRIEF EF subscales

One of the RCT/quasi‐experimental studies with a comparison group reported indirect measures of EF using the individual subscales of the BRIEF with a total of 78 participants (experimental *n* = 40; comparison *n* = 38; Wells et al., 2012). Listed below are the calculated effect sizes for each subscale:

- Initiate (SMD = 0.04, 95% CI = −0.40, 0.49)
- Monitoring (SMD = 0.25, 95% CI = −0.20, 0.70)
- Planning and organisation (SMD = −0.10, 95% CI = −0.55, 0.34)
- Organisation of materials (SMD = 0.25, 95% CI = –0.19, 0.70)

These single effect sizes suggest that participants receiving a psychological EF‐focused intervention did not significantly differ from comparison participants on indirect measures of various EFs.

One single‐group pre‐post study also reported the above BRIEF indirect measures of EF before and after an eligible intervention (Leung et al., 2015), yet did not report post‐intervention data to allow calculation of effect sizes.

## 6 DISCUSSION

### 6.1 Summary of main results

This review synthesises effect estimates from 11 studies to explore the impact of interventions designed to improve EF in children diagnosed with FASD or confirmed PAE. The current review separated EF by direct and indirect measures. This was based on literature highlighting poor convergence between these two forms of measurement across a range of conditions associated with impaired EF (Anderson et al., 2002; Mahone et al., 2002; Vriezen & Pigott, 2002), including FASD (Nguyen et al., 2014), with performance on standardised measures of EF conducted under ideal testing conditions not reflected in day‐to‐day functioning as reported by carers (Cross, 2014).

The meta‐analyses of RCT/quasi‐experimental studies reporting direct measures suggest that the effect of EF‐focused psychological interventions was in the expected direction (favouring the experimental condition), but did not reach statistical significance, for visual attention, cognitive flexibility, or attentional inhibition. Across studies using single‐group pre‐post intervention measures, the effect sizes ranged from small to large and suggest improvement in EFs, as measured by direct assessments. However, these single group studies are at high risk of bias and should be interpreted with caution.

The effect estimates for RCT/quasi‐experimental studies reporting indirect measures indicate that EF‐focused psychological interventions do not improve EFs compared to children who received no EF‐focused intervention (attention, behavioural regulation, effortful control, emotional control, global EF, inhibition, monitoring, organisation of materials, planning, shifting, working memory). The direction of the effects was mixed, with some effect estimates favouring the comparison condition and others favouring EF‐focused interventions. Across studies using single‐group pre‐post intervention measures, the effect sizes ranged from small to large and suggest improvement in EFs, as measured by indirect assessments. However, these single group studies are at high risk of bias and should be interpreted with caution.

### 6.2 Overall completeness and applicability of evidence

This review synthesises current evaluation evidence which evaluates the effectiveness of EF interventions for children with FASD or confirmed PAE. However, several considerations hinder the overall completeness and applicability of the evidence. First, the conclusions of this review are limited by a relatively small number of eligible studies (*k* = 11), all with small sample sizes and almost half of which are single‐group pre‐post intervention studies (*k* = 4). Second, some effect estimates are incomplete due to missing data in study reports and inability to obtain missing data from study authors. Third, the review was also unable to assess whether the impact of EF‐focused interventions change overtime, with almost all studies only reporting post‐intervention data or follow‐up data only for significant effects or the treatment group. Similarly, the review was unable to assess whether the impact of EF‐focused interventions varies by a range of important moderating variables (e.g., characteristics of the intervention, characteristics of the participants, type of EF targeted by the intervention). Fourth, most of the included studies were also conducted in North America (*k* = 10), with only one study conducted elsewhere (Australia). Therefore, the findings may not generalise to other cultures or geographical settings. This may be particularly pertinent in the area of FASD due to the strong cultural influences around patterns of alcohol consumption and differential expectations regarding normative child development across different cultural settings (Wang, 2018).

### 6.3 Quality of the evidence

The results of this review should be interpreted with utmost caution due to issues with the quality of the located and included evidence. First, effect estimates are based on either meta‐analyses using very few studies (*k* = 2–3 per analysis) or single studies. Second, all studies had very small sample sizes which impacts the precision and power of the estimates. Third, there is high to serious risk of bias across the corpus of studies. Collectively, these issues reduce the certainty and confidence in the review findings.

### 6.4 Potential biases in the review process

The systematic search for this review included 17 academic databases, 27 potential sources of grey literature, and various supplementary hand search strategies. As such, it is unlikely that any eligible studies were not captured by the review. The systematic search was updated to December 2020 to ensure the review provides a current representation of the state of the field. A potential bias in the review process was the omission of specialised education databases. We aim to include these in future updates of the review (e.g., ERIC).

All data extraction and effect size calculation was completed by two independent reviewers, thereby minimising the potential for any bias in the results of the review. Decision‐making regarding syntheses are clearly reported in this review, with sensitivity analyses conducted where possible to examine the impact of that decision‐making on the results. Overall, the review adhered to current Campbell Collaboration guidelines for reporting and conduct standards. Therefore, there is limited potential for bias arising from review process.

### 6.5 Agreements and disagreements with other studies or reviews

The current review found a lack of high quality evidence assessing the impact of interventions on EF in children diagnosed with FASD. According to the small number of extant studies, results of meta‐analysis suggest that there is limited evidence to support EF‐focused psychological interventions for children with FASD. This conclusion tends to differ from the included studies, many of which report favourable conclusions, albeit not consistently based on the full range of outcome data in study reports. Results of the current review echo conclusions of a number of previous reviews in the area, which also emphasise that the ability to draw strong conclusions regarding efficacy is limited by the relative dearth of research on interventions in FASD (Ospina et al., 2011; Peadon et al., 2009; Premji et al., 2007) and calls for further research (Bergeson, 2016). One review (Reid et al., 2015) concluded that there is emerging evidence for the effectiveness of interventions in early/middle childhood FASD. Similarly, Bertrand (2009) concluded that several interventions were shown effective in improving outcomes in FASD. However, both of these reviews included interventions targeting a broader range of outcomes (e.g., social skills, mathematics, safety skills). Additionally, meta‐analysis was not possible due to significant heterogeneity resulting from the broad scope of the review. This highlights the importance of conducting rigorous reviews and syntheses to support dissemination of more precise effect estimates to practitioners, researchers and policy‐makers.

## AUTHORS' CONCLUSIONS

### Implications for practice

There has been a notable increase in awareness in recent decades regarding the dangers and impacts of PAE. This is evidenced by the growing allocation of public funds across the developed world to understanding the consequences of prenatal exposure to alcohol, and the development of clear diagnostic guidelines and criteria across multiple countries. While much of the research in previous decades focused on the description and prevalence of FASD, greater emphasis is now being placed on interventions and their potential to improve the outcomes of children affected by FASD.

When considering the diffuse neurobiological impacts of PAE, damage to EFs may be among the most damaging to overall outcomes (Moffitt et al., 2011). EFs develop from an early age and underlie a range of everyday abilities in children and adults (Anderson et al., 2002) and so are an important area to target in early‐intervention efforts in the context of FASD. Yet the findings from this review suggest that EF‐focused psychological interventions may not be effective for improving EF in children with FASD, at least in the short‐term.

However, as noted above, these findings need to be interpreted with the utmost caution due to the limited evidence currently available for estimating overall effectiveness. Despite this limited evidence, these findings should not discourage further investigations of potential interventions to support children with FASD. FASD is associated with diffuse neurodevelopmental impairments that vary across individuals (Kodituwakku, 2009). It is possible that the most effective interventions may need to target multiple neurodevelopmental domains rather than focus predominantly on EF. In addition, outcomes may need to be tracked over longer periods of time to account for possible ‘sleeper effects’. That is, the true impact of interventions on EF may not be realised until later developmental years, as has been found in other early intervention programs (Bierman et al., 2014). Overall, there is much to be done to ensure that the quality of evaluation evidence keeps pace with the growing ambition to understand how best to treat children impacted by PAE.

### Implications for research

This review highlights the limited high‐quality evidence available for assessing the impact of EF‐focused psychological interventions on the EF of children with FASD. Therefore, there is an urgent need for more rigorous evaluation research to move beyond what should be considered to be only preliminary findings reported in this review. Future research should be conducted across different cultures and geographical settings to determine if these factors influence intervention effectiveness and thereby create the requirement to adjust standardised intervention approaches. In addition, future research needs to include larger sample sizes and pay close attention to design features that will improve the precision of effect estimates (e.g., RCT designs) and examine impact of interventions over the course of children's development (i.e., follow‐up measures).

## CONTRIBUTIONS OF AUTHORS

Joseph Betts is a registered psychologist with experience leading analytic projects as a statistician for central government (United Kingdom) and previous experience and training in systematic reviews. Joseph's PhD research is focused on exploring new mechanisms of early detection and treatment in FASD populations.

Elizabeth Eggins is a registered psychologist and a Research Fellow with a PhD in Clinical Psychology. She has co‐authored and managed numerous research projects grounded in systematic review methodology, including Campbell Collaboration and industry funded systematic, rapid and scoping reviews. She is an editor for the Campbell Collaboration Crime and Justice Coordinating Group.

Doctor Doug Shelton is a registered Paediatrician who specialises in community health and child development. Doctor Shelton's special interest area is FASD, for which he has received accolades for investigating the production of a comprehensive assessment and intervention service of children with FASD.

Doctor Haydn Till is a registered psychologist and endorsed Clinical Neuropsychologist with clinical and research experience across the age spectrum. His PhD was a systematic review and meta‐effect size analysis of cognition research in cardiac surgery. He currently holds the position of Clinical Associate Professor of Neuropsychology and sub‐investigator on genetic and clinical drug trials for dementia with the Queensland University of Technology. He currently leads the neuropsychological services of the Child Development Service at Gold Coast Health and supervises the clinical assessments conducted by the FASD clinics.

Paul Harnett is a Clinical Psychologist and Associate Professor in Criminology. He has worked clinically and conducted research in the field of child protection for three decades. He has an interest in program development for high risk families. His research includes evaluation of program efficacy and implementation, as well as decision making in the context of child protection.

Ned Chandler‐Mather is a provisionally registered psychologist with experience in leading experimental research and clinical trials in paediatric populations. Ned's PhD is focused on understanding and treating sleep problems in children with FASD.

Sharon Dawe is a Professor of Clinical Psychology and has a strong background in research pertaining to parental substance use and child/family functioning. Professor Dawe has made significant contributions in research, policy and practice areas.

Review tasks were distributed as follows:

- Content: Dawe, Betts, Eggins, Shelton, Till, Harnett
- Systematic review methods: Eggins, Betts, Chandler‐Mather
- Statistical analysis: Betts, Eggins
- Information retrieval: Betts, Eggins, Chandler‐Mather

## DECLARATIONS OF INTEREST

Professor Sharon Dawe and Associate Professor Paul Harnett are co‐founders and have been involved in the evaluation of the Parents under Pressure (PuP) program. PuP is a parenting program designed to improve outcomes (including executive function) in high‐risk, substance misusing families and would therefore qualify for inclusion as an eligible intervention. Both Dawe and Harnett have co‐authored a recent systematic review on interventions for improving outcomes in the context of FASD (Reid et al., 2015). To minimise potential bias, other authors screened and coded any papers co‐authored by Dawe or Harnett.

Professor Sharon Dawe, Doug Shelton and Haydn Till are investigators on the grant supporting this review (see Acknowledgements) and have published in the area of assessment and treatment of children with FASD. In addition, all study authors have been involved—to varying degrees—in the diagnostic assessment of children for FASD and their treatment. To minimise potential bias three procedures were implemented while producing this review: (1) authors Dawe, Shelton and Till were not involved in the coding or analysis stages of the review; (2) all studies were double‐coded independently by two authors (JB, NCH) and then checked for consistency and translated into summary tables by a third author (EE); and (3) all effect size calculations and analyses were conducted by Betts and then reviewed by Eggins.

Elizabeth Eggins is an Editor for the Campbell Crime and Justice Coordinating Group, however, she will not be privy to any internal editorial or Campbell Social Welfare Coordinating Group communications during the peer‐review process.

## DIFFERENCES BETWEEN PROTOCOL AND REVIEW

The review makes two deviations from the protocol. The protocol stated that studies would be included in the intervention was conducted in schools, hospitals or other clinics. Upon screening potentially eligible studies, it became clear that the intervention setting was not consistently or clearly reported. To provide a comprehensive review, studies that did not clearly specify the setting, but met all other eligibility criteria were included in the review.

The second deviation was adjusting the full‐text screening strategy due to the large number of documents. As independent double screening is not a mandatory MECCIR standard, an approach similar to other reviews published by the Campbell Collaboration was taken (e.g., Mazerolle et al., 2020). Specifically, a random sample of excluded full‐texts were reviewed by a second author to assess the consistency and accuracy of screening decisions. A 10% random sample was drawn across the full‐text exclusion criteria and only two disagreements were identified. The final inclusion/exclusion decision for these two studies was made via discussion between review authors.

## PUBLISHED NOTES

### 1 Characteristics of studies

**Characteristics of included studies**

Coles et al. (2015)

**Table:** 

Methods	Research design: Randomised controlled trial, yoked pairs*
	Comparison condition: No treatment
	Recruitment: Eligible children were identified by clinicians using medical records upon applying for clinical services at a paediatric neurodevelopmental exposure clinic. Information was provided to parents regarding the program before seeking consent and randomisation to conditions.
	Clustering: Unclear
	*Note: This study randomised participants to three conditions (GoFAR, FACELAND, control). The second treatment condition was equivalent to the GoFAR intervention, but included a computerised program (FACELAND) which aims to foster children's skills in identifying emotions (self and other). As this is an alternative intervention not aimed at fostering executive function, all comparisons are based on the GoFAR and control group participants.
Participants	Eligibility criteria: Children with a clinical diagnosis of FAS, or partial FAS, or significant alcohol‐related physical deformities (scores of more than 10 on dysmorphia checklist, Institute of Medicine/Hoyme Revision).
	Total referred sample: 20 (10 in both groups)
	Participants completing treatment: Exp = 7; Con = 9
	Child age (years; *M*/SD): Exp = 7.5 (1.4); Con = 7.0 (1.6)
	Child gender (% female): Exp = 30%; Con = 30%
	Child ethnicity:
	Caucasian: Exp = 40%; Con = 50% African American: Exp = 10%; Con = 40% Mixed: Exp = 40%; Con = 0%
	Socioeconomic status:
	Exp = household income category (6 = $35,000–49,999/year) Mean (SD): 7.4 (1.7) Con = household income category (6 = $35,000–49,999/year) Mean (SD): 5.8 (1.9)
	Other prenatal substance exposures reported: Not reported
	Caregiver education (years; *M*/SD): Exp = 14.5 (1.8); Con = 13.9 (1.7)
	Child protection involvement (% Yes): Exp = 80%; Con = 54.5%
	In adopted home/legal guardianship: Exp = 100%; Con = 100%
	Child birth head circumference (cm; *M*/SD): Exp = 32.3 (4.9); Con = 32.1 (2.3)
	Child birthweight (grams; *M*/SD): Exp = 2837 (1308); Con = 2444.5 (790.5)
	Child physical dysmorphology checklist score (*M*/SD): Exp = 20.0 (3.6); Con = 15.8 (7.0)
	Child general conceptual ability (*M*/SD): Exp = 89.3 (11.3); Con = 87.7 (11.3)
	Child taking medications for behaviour: Exp = 50%; Con = 70%
Interventions	Name of intervention: GoFAR
	Setting: Unclear
	Format:
	Individual face‐to‐face parenting sessions Individual child computer game sessions Parent‐and‐child therapy sessions
	Description:
	Five weekly computer game sessions (1 h) where children played GoFAR, which focuses on navigating obstacles with a character to unlock subsequent levels. Children cannot navigate freely, but must plan actions by placing symbols in environment before executing them (‘Focus and Plan’ step), then after action (‘Act’ step), they are required to recall their plan before moving to the next level (‘Reflect’ step). Length and frequency of sessions not provided in any reports of the study. Five parenting sessions (1 h) focused on learning and using the FAR methodology and techniques for managing child behaviour. These sessions ran concurrently with the child computer game sessions. Parenting sessions were conducted by a clinical psychology graduate students and postdoctoral fellows under the supervision of one study author. Session content: (a) FASD psychoeducation and impact of FASD on arousal behaviour regulation; (b) identifying child's arousal level and how to teach in calm alert state; (c) environmental modifications to promote behavioural regulation; (d) how to promote adaptive living skills and manage negative behaviours; (e) reflection on how to apply FAR process. Parents were provided with homework based on the content of the sessions. Mean time for parent sessions: 56.9 min (SD = 4.3). Five weekly Behaviour Analogue Therapy parent–child sessions focused on applying learning through activities that used toys as analogues for child's activities at home (e.g., putting doll to bed). The specific activities were individualised for each family. Children taught application of FAR techniques by therapist, under observation of parent(s), followed by parent implementation within session and weekly homework to continue application in the home environment. Length and frequency of sessions not provided in any reports of the study.
	Integrity monitoring: Child computer game sessions were monitored by staff supervised by the study authors. These staff were either an educational specialist, clinical psychological graduate student or trained undergraduate student. The following data was collected during observation: time spent playing the game, attention to the game, enthusiasm and number of prompts to stay on task. Parent therapy sessions were audio visually recorded and reviewed by study authors and therapists recorded whether homework was completed, number of pre‐defined therapy goals achieved and parents' understanding of session content.
Outcomes	Measures were administered before randomisation and after completion of the program (or equivalent for comparison group participants). Self‐report child disruptive behaviour was also measured at mid‐treatment.
	**Eligible outcomes (direct)**
	Inhibition (Coles et al., 2018)
	TOVA (errors of commission) NEPSY (inhibition/inhibition)
	Attention (visual‐spatial; Coles et al., 2018)
	TOVA (errors of omission)
	Attention (auditory; Coles et al., 2018)
	NEPSY (auditory attention scaled score)
	*Eligible outcomes (indirect)*
	Global executive function (Coles et al., 2018)
	Behavioural Rating Inventory of Executive Functioning (BRIEF, Global Executive Composite)
	Behavioural regulation (Coles et al., 2018)
	BRIEF (Behavioural Regulation Index)
	Meta‐cognition (Coles et al., 2018)
	BRIEF (Meta‐cognitive Index)
	Effortful control (Coles et al., 2018)
	Children's Behaviour Questionnaire (CBQ, effortful control composite)
Notes	Funding: National Institute of Health/National Institute on Alcohol Abuse and Alcoholism
	Study location: United States (Atlanta)

Risk of bias table

**Table:** 

Bias	Authors' judgement	Support for judgement
Randomisation	High risk	Comment: Unclear whether the allocation sequence was random or concealed. Quote: ‘Thirty families with children ages 5 to 10 years of age who were prenatally affected by alcohol were recruited and randomly assigned…control group participants served as time‐elapsed controls and were yoked to a participant in one of the treatment groups to minimise group differences in time between assessments’ (Coles et al., 2018).
		Comment: The results of baseline differences on outcomes measures not directly reported, but inspection of baseline data, reported for participants completing the study protocol, suggest small differences the TOVA and NEPSY. Quote: ‘Comparisons of demographic and family characteristics, birthweight and intellectual skills of the participants yielded no significant group differences’ (Coles et al., 2015).
Deviations from intended interventions	High risk	Comment: Due to the nature of the intervention and comparison conditions, participants and implementers likely aware of assignment. No information on whether there were deviations from the intended intervention due to the experimental context. Authors conducted ‘per‐protocol’ analysis whereby they excluded participants who did not complete the intended intervention from analyses, and this may have impacted the effect estimates.
Missing outcome data	Unclear risk	Comment: Reasons for missing data are reported by study authors and these reasons are likely unrelated to the true value of outcomes. That is, the reason for missing data is due to dropout and decisions by study authors to excluded participants who did not complete the entire intervention from analysis (see ‘deviations from intended interventions’). Quote: ‘results are based on the 25 participants completing the protocol’ (Coles et al., 2018) and ‘advanced statistical modelling of missing data was not performed as a result of the small sample size’ (Coles et al., 2015).
Outcome measurement	Low risk	Comment: Method of measurement appropriate, measurement of direct outcomes unlikely to change between groups or be influenced by knowledge of intervention received by participants as the assessment methods are standardised. Study authors likely collected outcome data, meaning they were aware of the intervention received by participants, but this was unlikely to have affected the results of direct standardised assessment methods. Indirect self‐report measures may be influenced by knowledge of the intervention.
Selection of reported result	Unclear risk	Comment: No pre‐specified analysis plan or protocol could be located. Multiple eligible outcome measures, with multiple eligible analyses across study reports (appear consistent). For direct outcomes, all available subscales for eligible outcomes appear to be reported. For indirect outcomes, some subscales are not reported (e.g., single subscales for BRIEF and CBCL).

Hutchison (2015)

**Table:** 

Methods	Research design: Single group with pre‐ and post‐intervention measures
	Recruitment: Three schools agreed to recruit children. Information packages and consent forms sent to guardians of children in the eligible age range who had been identified as having an FASD diagnosis or who had received services in relation to PAE or possible FASD. Guardians who returned consent forms were contacted by the researcher.
	Clustering: participants were enrolled across three schools (school 1 = 5; school 2 = 4; school 3 = 1)
Participants	Eligibility criteria: Aged 6–13 years, with a diagnosis of FASD or who had received services related to PAE. Children with a brain injury, other neurodevelopmental disorder diagnosis, or who could not complete baseline outcome measures were excluded from the study.
	Total referred sample: 10*
	Participants completing treatment: 9
	Other prenatal substance exposures reported: Not reported
	Age (years; *M*/SD): 10 (range: 6–12 years, no SD reported)
	Gender (% female)*: 50%
	Ethnicity: Not reported
	Cognitive ability:
	General IQ (M, range): 93 (81–106) Verbal IQ (M, range): 86 (66–113) Visual IQ (M, range): 102 (90–114)
	*Inconsistency in report. Page 28 states 9 participants, yet table data adds to 10. Data above is based on 9 participants, except where indicated.
Interventions	Name of intervention: The Caribbean Quest (CQ)
	Setting: School
	Format: Child computer game sessions (group/individual format not reported)
	Description: CQ is a serious video game designed to improve attention and working memory in children. The game consisted of five levels (mini‐games), each of which required a 90% accuracy rate or certain number of correct trials per level to pass and move onto the next level. Each level tested and trained either attention or working memory through interactive video game activities. The entire intervention lasted 12 h in total, distributed across 20–30 min sessions 2–5 times per week, over a period of 8–10 weeks.
	Integrity monitoring: Children played the video game under supervision of an Education Assistant (EA) trained in the implementation of the CQ. The lead author shadowed an initial session and modelled strategies to improve participant engagement. The author stayed in regular contact with EAs throughout the study period, to answer questions relating to implementation.
Outcomes	Measures were administered approximately 1 week before intervention commencement and 1 week after intervention completion
	*Eligible outcomes (direct)*
	Response inhibition
	NEPSY (inhibition/inhibition)
	Cognitive flexibility
	NEPSY (inhibition/switching) NEPSY (response set) NIH toolkit (dimensional change card sort)
	Verbal working memory
	WISC‐IV digit span (backwards)
	Visual working memory
	WISC‐IV spatial span (backwards)
	Attention (auditory)
	NEPSY auditory attention (combined score)
	Attention (inhibit)
	NIH toolkit (Flanker)
	*Eligible outcomes (indirect)*
	Global executive function
	Behavioural Rating Inventory of Executive Functioning (BRIEF, global executive function composite)
	Inhibition
	Behavioural Rating Inventory of Executive Functioning (BRIEF, Inhibit)
	Shifting
	BRIEF (shift)
	Behavioural regulation
	BRIEF (Behavioural Regulation Index)
	Meta‐cognitive Index
	BRIEF (Meta‐cognitive Index)
Notes	Funding: Not reported
	Study location: Canada (Keewatin and Winnipeg)

Risk of bias table

**Table:** 

Bias	Authors' judgement	Support for judgement
Randomisation	Unclear risk	
Deviations from intended interventions	Unclear risk	
Missing outcome data	Unclear risk	
Outcome measurement	Unclear risk	
Selection of reported result	Unclear risk	

Kerns et al. (2010)

**Table:** 

Methods	Research design: Single group with pre‐ and post‐intervention measures
	Recruitment: Caregivers of children with FASD were distributed invitations through the local school district
Participants	Eligibility criteria: Children who had received a diagnosis of FASD from a medical practitioner
	Total referred sample: 12
	Participants completing treatment: 10
	Other prenatal substance exposures reported: Not reported
	Age (years; *M*/SD): 12.3 (2.67)
	Gender (% female): 40%
	Ethnicity: Not reported
	Socioeconomic status: Not reported
	Cognitive ability (*M*/SD): 91 (13.17)
Interventions	Name of intervention: Computerised Progressive Attention Training
	Setting: School (library or special education area)
	Format: Individual child computer game sessions
	Description: A computerised game designed to improve attention in children aged 6 years and older. The intervention was delivered via laptop computer, facilitated by a research assistant or support teacher. The training consisted of a series of levels which challenge participants' attentional capabilities. Through the administration, deliberate use of meta‐cognitive strategies was encouraged (e.g., rehearsal strategies). The intervention lasted a total of 16 h in 4 × 30 min sessions per week.
	Integrity monitoring: Not reported
Outcomes	Measures were administered before and after the intervention (exact time‐point not reported)
	*Eligible outcomes (direct)*
	Response inhibition
	KiTAP (Happy Sad Ghost) KiTAP (Dragon's Castle) ANT‐C (Reaction Time)
	Verbal working memory
	Children's size ordering task WISC (spatial span backwards)
	Cognitive flexibility
	KiTAP (The owls)
	Attentional inhibition
	KiTAP (Ghost's Ball)
	Attention (auditory)
	TEA_Ch (Score)
	*Eligible outcomes (indirect)*
	Nil
Notes	Funding: Victoria Foundation and the Ministry of Children and Family Development, British Columbia
	Study location: Canada

Risk of bias table

**Table:** 

Bias	Authors' judgement	Support for judgement
Randomisation	Unclear risk	
Deviations from intended interventions	Unclear risk	
Missing outcome data	Unclear risk	
Outcome measurement	Unclear risk	
Selection of reported result	Unclear risk	

Leung et al. (2015)

**Table:** 

Methods	Research design: Single group with pre‐ and post‐intervention measures*
	Recruitment: Participants were recruited through a FASD clinic at a rehabilitation hospital, following a confirmed history of PAE.
	*Note: This study used an ineligible comparison group (children without FASD) and so was treated as a single group study.
Participants	Eligibility criteria: Children needed to have a confirmed history of PAE. Children were excluded if they had any of the following conditions: genetic disorders (e.g., Down's syndrome), severe neurodevelopmental disorders (e.g., autism) and/or significant motor/sensory impairments (e.g., cerebral palsy, blindness).
	Total referred sample: 27
	Participants completing treatment: 20
	Child age (years; *M*/SD): 9.2 (2.59)
	Child gender (% female): 55%
	Child ethnicity:
	Caucasian: 20% African American: 80%
	Socioeconomic status: 43.73 (10.20)—no further specification in study reports
	Other prenatal substance exposures reported: Not reported
	Placement status:
	Adopted home/legal guardianship: 75% Foster parents/other caregivers: 25%
	Number of placements (N/SD): 3.5 (2.71)
	Child cognitive ability (*M*/SD):
	General IQ = 88.55 (13.83) Verbal IQ = 86.10 (15.45) Visuospatial = 94.60 (11.36)
Interventions	Name of intervention: Cogmed^©^ Working Memory Intervention
	Setting: Family home
	Format: Individual face‐to‐face child computer game sessions
	Description: 5 × 15–45 min sessions per week over a 5‐week period (25 sessions in total) where children completed a Cogmed^©^ training module in each session, under the supervision of a caregiver. Cogmed^©^ is a computerised working memory intervention aiming to assist children with attention difficulties to improve both their attention and working memory. The JM version of the intervention for children aged 4–6 years uses shorter sessions (15 min), compared to the RM version which uses longer sessions (30–45 min) for older children aged 7–13 years.
	Integrity monitoring: Not formally monitored, however, informal reports from caregivers suggested that participants often took more time to complete the intervention components than specified in the Cogmed^©^ protocol. Reasons recorded included: need for frequent breaks, difficulty sustaining attention and school schedules.
Outcomes	Measures were administered before the intervention, after completion of the program and 5 weeks later.
	*Eligible outcomes (direct)*
	Attention (visual‐spatial)
	TOVA (errors of omission)
	Attention (auditory)
	NEPSY (auditory attention scaled score)
	Cognitive flexibility
	NEPSY (inhibition–switching) NEPSY (response set)
	Inhibition (response)
	TOVA (errors of commission) NEPSY (inhibition/inhibition)
	Working memory
	Verbal (AWMA: verbal working memory) Visual (AWMA: visual spatial working memory) Visual (WISC‐V: spatial span–backwards)
	*Eligible outcomes (indirect)*
	Behavioural Rating Inventory of Executive Functioning (BRIEF)
	Global Executive Composite Behavioural Regulation Index Meta‐cognitive Index Emotional Control Index Inhibition Shift Working memory Planning Organisation skills Monitoring skills
	Global executive function
	Conners Comprehensive Behaviour Rating Scales (executive function)
	Attention
	Conners Comprehensive Behaviour Rating Scales Behaviour Assessment System for Children (BASC‐2)
Notes	Funding: Not reported
	Study location: Canada (Alberta)

Risk of bias table

**Table:** 

Bias	Authors' judgement	Support for judgement
Randomisation	Unclear risk	
Deviations from intended interventions	Unclear risk	
Missing outcome data	Unclear risk	
Outcome measurement	Unclear risk	
Selection of reported result	Unclear risk	

Loomes et al. (2008)

**Table:** 

Methods	Research design: Matched quasi‐experimental (matched on ‘age and gender when possible’ (p. 116), allocation to conditions not described)
	Comparison condition: No treatment
	Recruitment: Child participants were recruited via a hospital FASD clinic, a medical FASD diagnostic clinics and other FASD community agencies and schools
Participants	Eligibility criteria: None specifically stated, although all children had ‘previously been diagnosed with an FASD (Alcohol‐Related Neurodevelopmental Disorder, Neurobehavioral Disorder: Alcohol Exposed, Static Encephalopathy: Alcohol Exposed)’ (p. 116).
	Total referred sample: Not reported (*n* = 33 tested)
	Participants completing treatment: Exp = 17; Con = 16
	Age (years; *M*/SD): Exp = 7 years 5 months (2.32); Con = 7 years 6 months (1.23)
	Gender (% female): Exp = 52.94%; Con = 31.25%
	Ethnicity (both groups combined):
	Indigenous: 57.58% Other: 52.42
	Socioeconomic status: Not reported
	Other prenatal substance exposures: Not reported
	Placement status (both groups combined):
	Adopted home: 54.5% Fostered: 18.2% Biological parent: 6% Grandparent carer: 21.2%
Interventions	Name of intervention: Rehearsal training
	Setting: Unclear
	Format: Individual face‐to‐face child sessions
	Description: Researchers taught children rehearsal strategies for remembering information (e.g., repeatedly whispering stimuli after presentation). Children then completed the same standardised test for working memory as completed at baseline. A second session occurred, on average, 10.6 days later (range: 6–21 days) where the child was reminded of the rehearsal strategy.
	Integrity monitoring: Throughout the experiment, assessors observed and recorded rehearsal strategies (e.g., whispering, moving lips or repeating the items) and asked children how they remembered the stimuli on the experimental tasks.
Outcomes	Measures were administered before the intervention, immediately after the first intervention session, and then immediately after the second intervention session.
	*Eligible outcomes (direct)*
	Working memory
	Working Memory Test Battery for Children (WMTB‐C)**Insufficient data for effect size calculation and data could not be sourced from study authors.
Notes	Funding: Alberta Mental Health Board and the Glenrose Hospital Clinical Research Fund
	Study location: Canada

Risk of bias table

**Table:** 

Bias	Authors' judgement	Support for judgement
Randomisation	Unclear risk	
Deviations from intended interventions	Unclear risk	
Missing outcome data	Unclear risk	
Outcome measurement	Unclear risk	
Selection of reported result	Unclear risk	

Nash (2012)

**Table:** 

Methods	Research design: Quasi‐randomised trial (alternating sequence of allocation)
	Comparison condition: Waitlist control.
	Recruitment: Undertaken at two clinics: Motherisk clinic at a children's hospital and at another FASD diagnostic facility in Ontario. Recruitment from the Motherisk clinic entailed a letter being sent to parents of children within the specified age range who had received a diagnosis along the FASD spectrum, followed by a screening interview. Recruitment from the diagnostic facility was based on parent‐initiated contact with the study coordinator following advertisement at FASD support groups.
Participants	Eligibility criteria: Child aged 8–12 years, previous diagnosis along FASD spectrum, IQ > 70.
	Total referred sample: 29 (14 assigned to Exp; 15 assigned to Con)
	Participants completing treatment: Exp = 12; Con = 13
	Age (years; *M*/SD): Exp = 10.3 (1.7); Con = 10.4 (1.3)
	Gender (% female): Exp = 50%; Con = 46%
	Ethnicity: Not reported
	Socioeconomic status:
	Low: Exp = 42%; Con = 69% Medium: Exp = 42%; Con = 8% High: Exp = 16%; Con = 23%
	Other prenatal substance exposures reported:
	Alcohol and cigarettes: Exp = 58%; Con = 77% Alcohol and secondary drugs: Exp = 67%; Con = 23%*
	Placement status:
	Adopted: Exp = 75%; Con = 54% Fostered: Exp = 17%; Con = 8% Biological relative: Exp = 8%; Con = 8%
	Birthweight (kilograms; *M*/SD): Exp = 2.9 (0.68); Con = 3.0 (0.74)
	Composite IQ: Exp = 86.3 (12.7); Con = 92.7 (15.3)
	Receptive language: Exp = 97.3 (17.1); Con = 99.5 (11.7)
	Comorbidity:
	ADHD: Exp = 42%; Con = 85%*ODD: = Exp: 25%; Con = 15% Language disorder: Exp = 50%; Con = 38% Anxiety disorder: Exp = 0%; Con = 15% Sensory disorder: Exp = 8%; Con = 8%
	Note. Demographics are for sample completing treatment, as reported in Nash (2012). Sample numbers and demographic details slightly vary across reports of this study. In addition, the eligibility criteria for the Soh et al. (2015) and Nash et al. (2017) report expand eligibility criteria so that children with a hospitalised head injury were not included. The Soh et al. (2015) report expands these criteria further to exclude children with a neurological abnormality, a debilitating/chronic medical condition or contraindication to MRI (e.g., braces).
	*Significantly different at baseline
Interventions	Name of intervention: *Alert Program for Self Regulation*®
	Setting: Hospital clinic
	Format: Individual face‐to‐face child sessions
	Description: 12 × 60 min therapy sessions across 12–14 weeks in developmentally appropriate therapy room (e.g., floor mats, large pillows, tent) without distracting visual/auditory stimuli. The Alert program focuses enhancing child self‐regulation by encouraging integration of cognitive processing, using a car engine analogy. The sessions are organised into three sequential stages: (1) identifying and labelling ‘engine levels’ through learning ‘engine words’ and ‘engine speeds’; (2) experimenting with ‘engine speeds’ and learning regulation strategies; (3) self‐selection of strategies for application outside therapy and review of learning. Before proceeding to subsequent stages, children need to pass ‘mile markers’, which represent their mastery of concepts. For this study, therapy was delivered by two senior doctoral level clinical psychology students who had completed training by the developers of the Alert program.
	Integrity monitoring: A selection of videoed sessions were observed by a clinical psychologist and the implementing therapists received bi‐weekly supervision with a senior clinician to ensure treatment was being delivered in a developmentally appropriate way for each child (e.g., use of visual tools, reinforcement strategies).
Outcomes	Measures were administered before treatment and within 2 weeks of completing the treatment program (or equivalent for comparison group participants).
	*Eligible outcomes (direct)*
	Response inhibition
	NEPSY (inhibition/inhibition combined)
	Attentional inhibition
	TEA‐Ch (Sky Search, attention score)
	Attention (visual‐spatial)
	TEA‐Ch (Sky Search, DT)
	Attention (auditory)
	TEA‐Ch (Score!)
	Cognitive flexibility
	NEPSY (inhibition/switching) CANTAB (intra/extra dimensional shift task)
	Planning
	CANTAB (stocking of Cambridge task)
	*Eligible outcomes (indirect)*
	Global executive function
	Behavioural Rating Inventory of Executive Functioning (BRIEF, Global Executive Composite)
	Behavioural regulation
	BRIEF (Behavioural Regulation Index)
	Inhibition
	BRIEF (Inhibit Index)
	Shifting
	BRIEF (Shift Index)
	Emotional control
	BRIEF (Emotional Control Index)
Notes	Funding: Charles Banting and Fredrick Best Canadian Institute of Health Research (CIHR) Canada Graduate Scholarship and CIHR operating grant.
	Study location: Canada

Risk of bias table

**Table:** 

Bias	Authors' judgement	Support for judgement
Randomisation	High risk	Comment: Allocation sequence was not random and unclear if randomisation was concealed. Quote: ‘Upon enrolment in the study, children were assigned in alternating sequence’ (Nash, 2012, p. 23).
		Comment: Baseline differences between intervention groups suggest there may have been a problem with randomisation. Quote: ‘Groups differed significantly at baseline in ADHD diagnoses and exposure to both alcohol and drugs. The DTC group had significantly more children diagnosed with ADHD…whereas the TXT group had a greater number exposed to both alcohol and drugs…There were no other significant differences between the TXT and DTC groups’ (Nash, 2012, p. 29).
Deviations from intended interventions	High risk	Comment: Participants likely aware of their assigned intervention, as they were presumably assigned following an informed consent process which detailed both conditions. Study authors appear to have implemented randomisation and also implemented the treatment intervention.
		No deviations are reported for earlier study reports (Nash, 2012; Nash et al., 2015, 2017), however the secondary report by Soh et al. (2015) indicates that one participant was re‐allocated from the treatment group to the control group due to scheduling issues for the family. The Soh et al. (2015) report also contains an additional four participants compared to the Nash et al. reports and so it is unclear whether this re‐allocated participant is in the participants reported by Nash et al. or resides within the additional four participants included in the Soh et al. (2015) report.
		The analysis was appropriate to estimate the effect of assignment to the intervention (intention‐to‐treat/modified intention‐to‐treat analyses).
Missing outcome data	High risk	Comment: Data not available for all randomised participants, results could possibly be biased by missing outcome data, and missingness may depend on its true value. Approximately 85% data available for each group. Loss of 2 of 14 participants in the treatment group and 3 of 15 in the waitlist control group. No analysis methods that correct for bias or sensitivity analyses reported by study authors. Reasons for missing data in Nash (2012) and Nash et al. (2015) appear to be unrelated to the eligible outcome data (i.e., custody/access issues, lost to follow‐up). However, reasons for missing data in Nash et al. (2017) may be related to the Go‐No‐Go outcome. Specifically two treatment cases were excluded from analyses because they refused to enter the fMRI scanner due to anxiety, whereas, two cases in the control group were excluded from analysis due to a dental implant and excessive motion. This issue likely only affects one outcome, but this cannot be verified as even though the number of participants and missing data is the same across all the Nash et al. reports, the reasons for missing data appear to differ.
Outcome measurement	Low risk	Comment: method of measurement appropriate, measurement of direct outcomes unlikely to change between groups or be influenced by knowledge of intervention received by participants as the assessment methods are standardised. Study authors likely collected outcome data, meaning they were aware of the intervention received by participants, but this was unlikely to have affected the results of direct standardised assessment methods. Indirect self‐report measures may be influenced by knowledge of the intervention.
Selection of reported result	Unclear risk	Comment: Trial registered, but does not contain a statistical analysis plan. Eligible outcomes measured in multiple ways and some analysed across multiple reports, however, results appear consistent. For direct outcomes, all available subscales for eligible outcomes appear to be reported. For indirect outcomes, some subscales are not reported (e.g., single subscales for BRIEF and CBCL).

Pei et al. (2011)

**Table:** 

Methods	Research design: Randomised controlled trial (Study 1), with comparison group from the initial RCT then used as an experimental group for a subsequent RCT (Study 2; both reported in Pei et al., 2011). Makela et al. (2019) then utilised the experimental group data from Study 2 in a single‐group pre‐post analysis.
	Comparison condition: Study 1 = TAU (computer games with no EF focus); Study 2 = Wait list control.
	Recruitment: Local schools contacted by study authors to identify children with FASD and the school sent caregivers information and consent packages to invite participation in the project.
Participants	Eligibility criteria: Not reported, all children reported to have diagnosis of FASD
	*Study 1*
	Total referred sample: 18
	Participants completing treatment: Not reported
	Other prenatal substance exposures reported: Not reported
	Age (years; *M*/range): Exp = 9.12 (range: 6–12); Con = 9.78 (6–12)
	Gender (% female): Exp = 77.77%; Con = 66.66%
	Ethnicity: Not reported
	Socioeconomic status: Not reported
	Cognitive ability (mean, no SD reported):
	General: Exp = 85.00; Con = 85.22 Visual: Exp = 92.33; Con = 94.78 Verbal: Exp = 82.33; Con = 80.22
	*Study 2*
	Total referred sample: 21 (including nine experimental participants from Study 1)
	Participants completing treatment: Exp = 7; Con = not reported in any study reports
	Other prenatal substance exposures reported: Not reported
	Age (years; *M*/range): Exp = 11.63 (range: 6–15); Con = 12.17 (6–15)
	Gender (% female): Exp = 50%; Con = 72.73%
	Ethnicity: Not reported
	Socioeconomic status: Not reported
	Cognitive ability (mean, no SD reported):
	General: Exp = 86.20; Con = 77.18 Visual: Exp = 93.40; Con = 84.92 Verbal: Exp = 83.00; Con = 76.18
Interventions	*Study 1 and 2*
	Name of intervention: Cognitive Carnival with meta‐cognitive strategy coaching
	Setting: School
	Format: Individual computer game and meta‐cognitive training sessions
	Description: 12‐week computerised intervention implement in twice weekly sessions of 30 min each. Cognitive Carnival is a computerised intervention designed to challenge and develop children's attention, inhibitory control and working memory over a series of levels. It consists of three games that challenge different EF abilities, each comprised of 15 levels requiring 90% accuracy to progress. Three research assistants were trained in the role of interventionist, who taught and encouraged children to apply meta‐cognitive strategies during game play (e.g., rehearsal to scaffold working memory). The meta‐cognitive training followed a sequence of (a) identifying the issue; (b) strategy planning; and (c) reinforcement of the specific strategy.
	Integrity monitoring: Interventionists kept record of meta‐cognitive strategies that were either taught by themselves or used spontaneously by participants.
Outcomes	Eligible outcomes were measured before and after the intervention or approximately 12 weeks after baseline
	*Eligible outcomes (direct)*
	Attention (Pei et al., 2011)
	Study 1: NEPSY (auditory attention) Study 1: Farm animals game (omission errors, computerised continuous performance task) Study 2: KiTAP (no exact specification of subtest aside from classification by authors as a measure of attention)
	Inhibition (Pei et al., 2011)
	Study 1: NEPSY (exact subtest not specified, but subtests drawn from attention & executive function domain which contains measures of inhibition) Study 1: Day/night task (paper‐based Stroop‐like task) Study 1: Computerised go/no‐go (no further specification provided in study reports) Study 1: Farm animals game (commission errors, computerised continuous performance task) Study 2: Tasks of executive control
	Working memory (Pei et al., 2011)
	Study 1 and 2: Weschler Intelligence Scale for Children (spatial span) Study 1 and 2: Working Memory Test Battery for Children (WMTB‐C, Digit Recall and Block Recall) Study 2: Tasks of executive control
	Meta‐cognition (Makela et al., 2019)
	Meta‐cognitive strategy checklist (completed during intervention only, no baseline)
	*Eligible outcomes (indirect)*
	Study 1: General executive function using BRIEF, parent and teacher report (subscales not specified) Study 1: Attention using Conor's Rating Scales Revised, parent and teacher report Study 2: Attention Deficit Disorders Evaluation Scale (ADDES), parent and teacher report (Study 2)
Notes	Funding: Project funded by the Alberta Centre for Children, Family and Community Research
	Study location: Canada (Alberta)

Risk of bias table

**Table:** 

Bias	Authors' judgement	Support for judgement
Randomisation	Unclear risk	Comment: Unclear if allocation sequence was random, as the method only specifies that the study was a randomised controlled trial (Study 1 and Study 2). No information on whether the allocation sequence was concealed (Study 1 and Study 2). No obvious baseline differences between groups based on demographics (not statistically tested) for Study 1, and no report of eligible outcome measures at baseline to determine if there were differences between groups. Some baseline balances based on demographics (not statistically tested) for Study 2, and no report of eligible outcome measures at baseline to determine if there were differences between groups.
Deviations from intended interventions	High risk	Comment: Due to the nature of the intervention and comparison conditions, participants and implementers likely aware of assignment. No information on whether there were deviations from the intended intervention due to the experimental context. No clear information reported on the statistical analysis to assess if the analysis was approach to estimate the effect of assignment to intervention.
Missing outcome data	High risk	Comment: No clear information on the statistical analysis or results to be able to assess whether (a) outcome data was available for all, or nearly all, participants randomised; or (b) whether the outcome was biased by missing outcome data.
Outcome measurement	Low risk	Comment: Method of measurement appropriate, measurement of direct outcomes unlikely to change between groups or be influenced by participants' knowledge of intervention received by participants as the assessment methods are standardised. Quote: ‘Pre‐ and post‐testing was completed by research assistants blind to group assignment’ (Pei et al., 2011, p. 13). This further reduces the bias for direct assessments of EF, however, indirect self‐report measures may be influenced by knowledge of the intervention.
Selection of reported result	High risk	Comment: No a‐priori protocol or analysis plan could be located. Multiple eligible outcome measures for EF, but insufficient detail reported on the analysis and results to assess for risk of bias associated with selective reporting. Some graphical figures and discussion of results in Pei et al. (2011) report, however only for a select number of all measured outcomes specified in the methodology section of the report.

Petrenko et al. (2017)

**Table:** 

Methods	Research design: Randomised controlled trial with block randomisation (blocks: age [4–5 and 6–8 years] and gender)
	Comparison condition: Treatment‐as‐usual (neuropsychological evaluation and customised community referrals)
	Recruitment: Purposive sampling via health practitioner referrals and promotional activities (e.g., flyers, presentations at parenting groups)
	Clustering: Yes (two study sites, recruitment over 2 years, two groups per year with three to four participants per group)
Participants	Eligibility criteria: Participants were required to meet three criteria: (1) FASD diagnosis based on diagnostic guidelines (Revised Institute of Medicine, Hoyme et al., 2005) or ‘confirmed history of PAE’; (2) child aged 4–8 years of age; and (3) family resided within feasible distance of two study sites.
	Total eligible referred participants: 30 (Exp = 19; Con = 11)
	Participants beginning treatment: Exp = 16; Con = 10
	Participants completing treatment: Exp = 15; Con = 9
	Other prenatal substance exposures reported: No
	*Child participants*
	Formal FAS/pFAS diagnosis: Exp = 5.8%; Con = 35.7%
	Age (years; *M*/SD): Exp = 6.52 (1.31); Con = 6.59 (1.28)
	Gender (% female): Exp = 37.5%; Con = 7.1%
	Ethnicity:
	Caucasian: Exp = 81.3%; Con = 85.7% African American: Exp = 25%; Con = 21.4% Hispanic: Exp = 12.5%; Con = 14.3% Native American: Exp = 6.3%; Con = 0% Asian: Exp = 6.3%; Con = 0%
	Placement status:
	Foster/adoptive: Exp = 81.3%; Con = 71.4% Relative: Exp = 18.8%; Con = 28.6%
	*Caregiver participants*
	Socioeconomic status (*M/*SD): Exp = $90,132 ($49,378); Con = $74,128 ($28,386)
	Age (years; *M*/SD): Exp = 45.77 (8.97); Con = 48.19 (8.19)
	Gender (% female): Exp = 93.8%; Con = 78.6%
	Ethnicity:
	Caucasian: Exp = 87.5%; Con = 100% African American: Exp = 6.3%; Con = 7.1% Hispanic: Exp = 6.3%; Con = 0% Native American: Exp = 6.3%; Con = 0%
	Education:
	High school: Exp = 6.3%; Con = 35.7% Associates degree: Exp = 18.8%; Con = 35.7% Bachelor's degree: Exp = 31.3%; Con = 21.4% Graduate degree: Exp = 37.6%; Con = 7.1%
	Marital status:
	Married/living with partner: Exp = 68.8%; Con = 78.5% Single parent: Exp = 31.2%; Con = 21.5%
	Note: Authors re‐allocated three participants assigned to the experimental condition who did not complete the treatment to the comparison condition. Therefore, reported participant demographics are based on 16 experimental participants and 14 comparison participants (three of which were originally allocated to the experimental condition).
Interventions	Name of intervention: Families on track
	Setting: Family home (parent consultation component) and university/community settings (child skills group component)
	Format:
	Individual face‐to‐face parent consultations (weekly, 90‐min each) Group face‐to‐face child skills groups (fortnightly, 90‐min each)
	Description: 30‐week program comprised of two components delivered by either the first author of the study or advanced graduate clinical or school psychology students.
	Child skills sessions using an adaptation of the Preschool/Kindergarten Promoting Alternative Thinking Strategies (PATHS) curriculum. Groups were comprised of study participants and other children who were not study participants (e.g., siblings and typically developing same‐aged peers). Each session was co‐led, with support provided by two undergraduate students, and adhered to the following agenda: (a) welcome activity; (b) PATHS lesson; (c) break (snack); (d) activities designed to encourage application of skills; and (e) free play. Parent consultations using an adaptation of the Families Moving Forward (FMF) program, typically delivered by child's PATHS group leader. Sessions generally adhered to the following agenda: (a) review of PATHS skills taught in child groups; (b) discussion of ways to apply skills in the home; and (c) specific behavioural observations of the child from PATHS group, including adaption to behavioural plans.
	Integrity monitoring: Individual and group supervision meetings with between study first author and practitioners implementing intervention components (2–3 h per week).
Outcomes	Measures were administered by blinded research assistants at baseline (unclear if before or after randomisation), approximately 12 and 18 months later.
	*Eligible outcomes (indirect)*
	Behavioural regulation:
	Emotion Regulation Checklist (Lability/Negativity Scale)
Notes	Funding: National Institute of Alcohol Abuse and Alcoholism (K01AA020486)
	Study location: United States

Risk of bias table

**Table:** 

Bias	Authors' judgement	Support for judgement
Randomisation	Unclear risk	Comment: Unclear whether the allocation sequence was random or concealed. Quote: ‘Families were randomised to either Families on Track Integrated Preventive Intervention or an active control of neuropsychological assessment and personalised community referrals’ (p. 1340). ‘Randomisation was stratified by child sex and age (4‐5, 6‐8)’ (p. 1344). Comment: No other information on randomisation reported.
		Comment: Baseline differences between intervention groups do not suggest there was a problem with the randomisation process. Quote: ‘There were no statistically significant differences in participant characteristics or baseline levels of functioning’ (p. 1349)
Deviations from intended interventions	High risk	Comment: Due to the nature of the intervention and comparison conditions, participants and implementers likely aware of assignment (Quote: ‘All intervention components were delivered by the first author and advanced graduate students in clinical or school psychology’ [p. 1350]).
		Deviations from the intended protocol reported by authors. Three participants who allocated to the intervention, but that declined the intervention, were combined with nine participants in the comparison group at the post‐intervention time‐point. Fidelity was monitored, with no issues reported. However, if the three reassigned participants had higher or lower levels on eligible outcomes, the estimate of the differences between groups may have been impacted. No specific data comparing the three re‐assigned participants are reported by authors.
		Authors conducted ‘as treated’ analysis whereby they excluded participants who did not complete the intended intervention from analyses. Failure to analyse participants as they were randomised may have impacted the results (10% [3/30] of the allocated sample analysed in a different group than originally assigned).
Missing outcome data	Low risk	Data reported for all randomised participants for initial study report, which was used to allocate the risk of bias rating for this domain. There was a loss of 6 participants in the follow‐up report (*n* = 5 experimental; *n* = 1 comparison). Based on the changes in means for the eligible outcome between the initial study report and the follow‐up report at baseline and post‐intervention, the follow‐up results may be impacted by the missing data. It is unclear if the missingness is due the true value of the outcome. Risk of bias was not downgraded for the overall study to ensure the studies included in the risk of bias analysis were comparable. That is, all other studies had baseline and post‐intervention outcome measures only, and downgrading this study based on the follow‐up attrition would render the studies less comparable on this domain.
Outcome measurement	High risk	Comment: method of measurement for the eligible outcome appropriate and unlikely to have differed between groups (standardised self‐report instruments). Outcome assessors possibly aware of the intervention received by participants: ‘Trained research assistants completed visits, which averaged 2 h. Attempts were made to keep research assistants blind to intervention condition’ (p. 1351). Assessment of the outcome may have been influenced by knowledge of the intervention as participants (parents) completed a self‐report measure of the eligible outcome and would have known their assigned condition. It could be argued that parents in the treatment group would have expectations or be vigilant to improvements in the eligible outcome (emotion regulation) and those in the comparison condition may expect no change.
Selection of reported result	Unclear risk	Comment: No a‐priori protocol or analysis plan could be located, all data on the single eligible outcome reported by authors. However, there were two eligible analyses of the outcome, results of which differed for the baseline‐post intervention effect. The difference, however is due to attrition rather than selective reporting.

Reid et al. (2017)

**Table:** 

Methods	Research design: Single group with pre‐ and post‐intervention measures
	Recruitment: Treating clinicians at a FASD diagnostic service referred families to the study
Participants	Eligibility criteria: Children were required to live locally near the referring service and have a diagnosis of FASD (all were diagnosed with a 4‐digit Diagnostic Code).
	Total eligible referred participants: Not reported
	Participants beginning treatment: 3
	Participants completing treatment: 2
	Other prenatal substance exposures reported: Not reported
	*Child participants*
	Age (years): Child 1 = 9; Child 2 = 12; Child 3 = 11
	Gender (% female): 100%
	Ethnicity: Not reported
	Cognitive ability: Child 1 = low average; Child 2 and 3 = average
	Adaptive behaviour: All in extremely low range
	Child behaviour (CBCL): All in clinical range
	*Caregiver participants (2 per child)*
	Age (years): Ranged from 43 to 57.
	Gender (% female): 50%
	Education: 5 = high school 1 = university
	Type of caregiver: 1 kinship family; 1 adoptive family; 1 biological family
	Existing mental health: present in all families (included depression, PTSD, panic disorder)
Interventions	Name of intervention: Parents under Pressure (PuP), adapted
	Setting: Family home/clinic (family's choice)
	Format: Parent‐and‐child therapy sessions
	Description: Weekly to fortnightly sessions lasting 1–2 h (Family 1 = 17 sessions total; Family 2 = 21 sessions total) with each family. Treatment begins with a holistic assessment of the family's needs, which guides the conceptualisation and tailored treatment approach. The usual PuP model was adapted by adding psychoeducation about the neurobehavioral impacts of FASD and specific self‐regulation strategies. Each session began with a review of the prior week's events and then proceeded to (a) discussion, application and problem‐solving in relation to self‐regulation strategies; and (b) discussion, application and problem‐solving in relation other issues experienced by the family. The implementing psychologist worked with parents to support the implementation of self‐regulation strategies between sessions, and also directly with children to teach and encourage the use of self‐regulation strategies. Example exercises included age‐appropriate mindfulness exercises taught through books, in‐situ practice and applications on electronic devices. The standardised Parent Workbook was used to guide strategies to support overall family wellbeing.
	Integrity monitoring: Fortnightly supervision with program developer and explicit fidelity checks in to the intervention, asking participants to discuss how strategies had been implemented throughout training.
Outcomes	Measures were administered at baseline, post‐intervention and follow‐up (3 months).
	*Eligible outcomes (direct)*
	Inhibition
	NEPSY (inhibition/inhibition combined)
	Cognitive flexibility
	NEPSY (inhibition/switching)
	*Eligible outcomes (indirect)*
	Global executive function
	Behavioural Rating Inventory of Executive Functioning (BRIEF, Global Executive Composite)
	Behavioural regulation
	BRIEF (Behavioural Regulation Index)
	Meta‐cognition
	BRIEF (Meta‐cognitive Index)
Notes	Funding: First author was supported by University PhD Scholarship and Australian Government Research Training Program Scholarship program
	Study location: Australia

Risk of bias table

**Table:** 

Bias	Authors' judgement	Support for judgement
Randomisation	Unclear risk	
Deviations from intended interventions	Unclear risk	
Missing outcome data	Unclear risk	
Outcome measurement	Unclear risk	
Selection of reported result	Unclear risk	

Vernescu (2008)

**Table:** 

Methods	Research design: Randomised controlled trial (matched pairs randomised to condition)
	Comparison condition: Treatment‐as‐usual (same amount of individualised contact as treatment for children, with academic concepts being taught with a multi‐modal approach (e.g., art, games, worksheets), with some individualisation based on child needs and skills).
	Recruitment: Not reported.
Participants	Eligibility criteria: Previous diagnosis of FASD made by a physician, absence of any other developmental disorder diagnoses.
	Total referred sample: 20
	Participants completing treatment: Exp = 10; Con = 10
	Age (years; *M*/SD): Exp = 9.54 (1.58); Con = 9.5 (1.62)
	Gender (% female): 55% (group split not reported)
	Ethnicity: Not reported
	Socioeconomic status: Not reported
	Other prenatal substance exposures reported: Not reported
Interventions	Name of intervention: *Pay Attention!* Program
	Setting: School
	Format: Individual face‐to‐face child sessions
	Description: Twelve daily 30‐min sessions during the school day or after school, with the total intervention period spanning 2.5–3.5 weeks. Teachers administered training to children in a small, quiet room in the school. The intervention following the *Pay Attention!* protocol, with some minor adjustments to some of the materials to suit the participants age and developmental ability and use of visual and auditory sustained attention tasks only. Example tasks included: sorting cards into categories or listening to auditory stimuli and pressing a buzzer when hearing the target stimuli. Tasks are hierarchically structured so that children only progressed to more difficult tasks once they reached a specific performance threshold for a task (e.g., 90% accuracy on each timed task). Children were not encouraged to practice outside of training sessions and were not taught any specific attention strategies to use during sessions.
	Integrity monitoring: Unclear
Outcomes	Measures were administered 1 week before the intervention and within 1 week after completion of the program.
	*Eligible outcomes (direct)*
	Attentional inhibition
	TEA‐Ch Map Mission*TEA‐Ch Sky Search (attention score)
	Response inhibition
	KiTAP Dragon's Castle (commission errors) KiTAP Happy‐Sad Ghost
	Attention (visual‐spatial)
	KiTAP (Ghost's Ball, correct responses) TEA‐Ch Sky Search (DT)*
	Attention (auditory)
	TEA‐Ch (Code Transmission, correct responses 0–14 min) TEA‐Ch (Score!)
	Cognitive flexibility
	TEA‐Ch (Creature Counting, scaled score) KiTAP (The owls)*
	*Eligible outcomes (indirect)*
	All subscales of the Behavioural Rating Inventory of Executive Functioning (BRIEF). However, only baseline data was reported, along with results of *t*‐tests for some of the subscales. Therefore, this outcome was not included in meta‐analyses.
	*Insufficient or no data reported by authors to calculate effect sizes, author could not be contacted.
Notes	Funding: Newfoundland and Labrador Centre for Applied Health Research (NLCAHR) and the Scottish Rite Charitable Foundation of Canada
	Study location: Canada

Risk of bias table

**Table:** 

Bias	Authors' judgement	Support for judgement
Randomisation	Unclear risk	Comment: Unclear if the allocation sequence was random or concealed. Quote: ‘Children were randomly assigned to training and control conditions’ (p. 62) within pairs matched on age and non‐verbal IQ. No other randomisation procedures specified.
		Baseline differences between intervention groups suggest there was not problems with randomisation, as independent *t*‐tests indicated no statistically significant differences between groups on outcome measures. However, there was no formal testing of between group differences on demographics and minimal demographic data reported between groups.
Deviations from intended interventions	Unclear risk	Comment: Participants likely aware of their assigned intervention, as they were assigned following an informed consent process which detailed both conditions. Study author delivered the intervention and so was aware of the participants' assignment. No deviations are reported for the intended intervention. The analysis was appropriate to estimate the effect of assignment to the intervention (intention‐to‐treat/modified intention‐to‐treat analyses).
Missing outcome data	High risk	Comment: Data was not available for all, or nearly all, participants randomised, whereby post‐intervention data was not consistently reported for all outcomes measured. It is unclear whether this missing data may have impacted the results.
Outcome measurement	Low risk	Comment: Method of measurement appropriate, measurement of direct outcomes unlikely to change between groups or be influenced by knowledge of intervention received by participants as the assessment methods are standardised. Study author likely collected outcome data, meaning they were aware of the intervention received by participants, but this was unlikely to have affected the results of direct standardised assessment methods. Indirect self‐report measures may be influenced by knowledge of the intervention.
Selection of reported result	High risk	Comment: No a‐priori protocol or analysis plan could be located. Multiple eligible outcome measures, but appears to be only one eligible analyses of the data. All subscales for eligible outcomes reported for baseline time‐point, but only statistically significant post‐intervention results are reported, thereby suggesting selective reporting.

Wells et al. (2012)

**Table:** 

Methods	Research design: Randomised controlled trial
	Comparison condition: No treatment
	Recruitment: Participants were sourced from the general child welfare population of the Illinois Department of Children and Family Services (DCFS). Consent was obtained from the child's foster/adoptive parent and also (as relevant) the Office of the Guardian of DCFS.
Participants	Eligibility criteria: Children aged 6–11:9 years who met criteria for FAS, partial FAS or ARND. Children were ineligible for participation if they had a history of serious head trauma, evidence of lead poisoning (current or historical) or genetic dysmorphic syndrome other than one related to PAE. Of the enrolled children, 21 had a diagnosis of foetal alcohol syndrome, 10 had a diagnosis of partial foetal alcohol syndrome and 47 had a diagnosis of alcohol‐related neurodevelopmental disorder.
	Total referred sample: 90
	Total beginning and completing treatment: Exp = 40; Con = 38
	Age (years; *M*/SD): Exp = 8.12 (1.48); Con = 9.24 (1.36)
	Gender (% female): Exp = 32.5%; Con = 31.6%
	Ethnicity:
	Caucasian: Exp = 30%; Con = 44.7% African American: Exp = 47.5%; Con = 36.8% Hispanic: Exp = 7.5%; Con = 0% Native America: Exp = 0%; Con = 2.6% Mixed: Exp = 15%; Con = 15.8%
	Socioeconomic status: Not reported
	Other prenatal substance exposures:
	Tobacco: Exp = 36.4%; Con = 37.5% Cocaine: Exp = 56.8%; Con = 44.1% Other: Exp = 22.5%; Con = 21.1%
	Placement status:
	Adoptive: Exp = 84.6%; Con = 84.2% Fostered: Exp = 27%; Con = 13.2%
	Comorbidity:
	ADHD: Exp = 67.5%; Con = 73.7% Anxiety disorder: Exp = 5.0%; Con = 7.9% Attachment disorder: Exp = 12.5; Con = 10.5% Disruptive disorder: Exp = 10.0%; Con = 5.3% Elimination disorder: Exp = 12.5%; Con = 15.8% Mood disorder: Exp = 10.0%; Con = 26.3% PTSD: Exp = 22.5%; Con = 21.1%
Interventions	Name of intervention: Neurocognitive Habilitation Program
	Setting: Unclear
	Format:
	Group face‐to‐face caregiver psychoeducational sessions Group face‐to‐face child intervention sessions Caregiver‐and‐child practice sessions
	Description:
	12‐week program comprised of 12 × 75 min sessions delivered by doctoral and master's‐level therapists, where by parent and child sessions ran concurrently and the final 15–30 min was dedicated to parent–child practising of the skills learnt. Caregiver psychoeducational sessions focused on providing information on FASD and skills‐based content: (a) for recognising their child's arousal level; (b) strategies to moderate child arousal and behaviour; (c) developmental accommodations for their child. Child sessions incorporated elements of the Alert program (e.g., engine to represent arousal, self‐regulation strategies) and other strategies used to treat traumatic brain injuries (e.g., strategies to improve memory). Each session followed a consistent structure: check‐in and review, group activity or learning concept, sensory snack and a ‘wind down’; activity (e.g., art) before joining with their caregivers.
	Integrity monitoring: Treatment manual guided intervention and practitioners met to discuss implementation and fidelity issues.
Outcomes	Measures were administered before randomisation and within 7 months from baseline (or equivalent for comparison group participants).
	*Eligible outcomes (indirect)*
	Inhibition
	Behavioural Rating Inventory of Executive Functioning (BRIEF, Inhibit Index)
	Shifting
	BRIEF (Shift Index)
	Emotional control
	BRIEF (Emotional Control Index)
	Initiation
	BRIEF (Initiate Index)
	Working memory
	BRIEF (Working Memory Index)
	Planning and organisation
	BRIEF (Plan/Organise Index)
	Organisation
	BRIEF (Organisation of Materials Index)
	Monitoring
	BRIEF (Monitor Index)
Notes	Funding: Grant U84/CCU520164 from the Centres for Disease Control and Prevention, US Department of Health and Human Services
	Study location: United States

Risk of bias table

**Table:** 

Bias	Authors' judgement	Support for judgement
Randomisation	High risk	Comment: evidence for random allocation, but unclear if randomisation was concealed. Quote: ‘The children were then placed into groups using simple random assignment by means of a table of odd and even random numbers. No predetermined allocation sequence was used’ (p. 212).
		Baseline differences between intervention groups suggest there was not problems with randomisation. Quote: ‘The demographic characteristics of the treatment and control groups, including race and ethnicity as classified by the children's primary caregiver and DCFS, were similar, except that the mean age of the control group was significantly greater than that of the study group’ (p. 29).
Deviations from intended interventions	Unclear risk	Comment: Participants likely aware of their assigned intervention, as they were assigned following an informed consent process which detailed both conditions. Intervention was implemented by postgraduate clinicians and it is unclear if these clinicians are also authors of or involved in the evaluation. No deviations from intended intervention reported. The analysis was appropriate to estimate the effect of assignment to the intervention (intention‐to‐treat/modified intention‐to‐treat analyses).
Missing outcome data	Low risk	Data reported for all randomised participants.
Outcome measurement	High risk	Comment: method of measurement appropriate, measurement of direct outcomes unlikely to change between groups or be influenced by knowledge of intervention received by participants as the assessment methods are standardised. Outcome assessors unaware of assignment. Quote: ‘Repeated follow‐up measures were administered 7 months after enrolment, which was usually 2 to 3 months after treatment concluded, by psychologists blinded to the child's group assignment’ (p. 23). However, intervention participants (parents) completed a self‐report measure of the outcome eligible for this review and would have known their assigned condition. It could be argued that parents in the treatment group would have expectations or be vigilant to improvements in the eligible outcome (behavioural indicators of executive function) and those in the comparison condition may expect no change.
Selection of reported result	Unclear risk	Comment: Although trial was registered before publication of results, the trial register does not list the executive function measures reported. Only one measure of the eligible outcome and data from all time‐points reported by authors. No analysis intentions could be located, however, all data reported for the eligible outcome in terms of means, standard deviations and statistical analyses examining within and between subjects effects.

Additional participant sociodemographic data is included beyond that in the coding form (Supporting Information: Appendix 3), if reported by study authors.

**Characteristics of excluded studies**

**Table:** 

Study	Reason for exclusion
Baker (2013)	Ineligible intervention (not focused on EF)
Chandrasena et al. (2009)	Ineligible intervention (not focused on EF)
Coles et al. (2009)	Ineligible intervention (not focused on EF)
Coles (2005)	Ineligible intervention (not focused on EF)
Coriale et al. (2013)	Ineligible intervention (no intervention evaluated)
Cyr (2011)	Ineligible intervention (no intervention evaluated)
Davis et al. (2011)	Ineligible intervention (no intervention evaluated)
De villiers (2005)	Ineligible intervention (not focused on EF)
Dorrie et al. (2014)	Ineligible intervention (no intervention evaluated)
Fernández‐Jaén (2011)	Ineligible intervention (no intervention evaluated)
Hollar (2012)	Ineligible intervention (no intervention evaluated)
Kable et al. (2012)	Ineligible intervention (not focused on EF)
Kerns et al. (2017)	Sample comprised of children with ASD and FASD, with no separation of results by diagnosis
Kodituwakku (2010)	Ineligible intervention (no intervention evaluated)
Koren et al. (2014)	Ineligible intervention (no intervention evaluated)
Kully‐Martens et al. (2012)	Ineligible intervention (no intervention evaluated)
Kully‐Martens et al. (2018)	Ineligible intervention (not focused on EF)
Kully‐Martens et al. (2013)	Ineligible intervention (not focused on EF)
Millians and Coles (2014)	Ineligible intervention (cannot isolate EF component from other ineligible components)
Moore and Tolle (2008)	Ineligible intervention (no intervention evaluated)
Mulvihill (2008)	Ineligible intervention (not focused on EF)
Olson and Montague (2011)	Ineligible intervention (no intervention evaluated)
Paley and O'Connor (2009)	Ineligible intervention (no intervention evaluated)
Paley and O'Connor (2011)	Ineligible intervention (no intervention evaluated)
Peadon and Elizabeth (2010)	Ineligible intervention (no intervention evaluated)
Petrenko et al. (2017)	Ineligible intervention (no intervention evaluated)
Reid et al. (2019)	Ineligible intervention (not focused on EF)
Sabou et al. (2012)	Ineligible intervention (no intervention evaluated)
Senturias and Burns (2014)	Ineligible intervention (no intervention evaluated)
Singal (2018)	Ineligible intervention (no intervention evaluated)
Timler et al. (2005)	Ineligible intervention (not focused on EF)
Turner et al. (2015)	Ineligible intervention (no intervention evaluated)
Watson and Westby (2003)	Ineligible intervention (no intervention evaluated)
Zarnegar et al. (2016)	Ineligible intervention (intervention not explicitly focused on EF—cannot isolate EF component from other ineligible components)
Zevenbergen and Ferraro (2001)	Ineligible intervention (not focused on EF)

**Characteristics of ongoing studies**

**Table:** 

Louw (2019)	
Study name	A randomised control trial of a custom developed computer game to improve executive functioning in 4‐ to 6‐year‐old children exposed to alcohol in utero
Methods	Randomised control trial
Participants	Children (4–6 years) with PAE and unexposed children
Interventions	Therapeutic computer game
Outcomes	Brain function
Starting date	February 2018 to October 2018
Contact information	Mr Jaco Louw, 42 Bloemhof Road, Belville, Cape Town 7535, South Africa
Notes	
Luow (2019)	
Study name	Evaluation of a custom‐developed computer game to improve executive functioning in 4‐ to 6‐year‐old children exposed to alcohol in utero: protocol for a feasibility randomised controlled trial
Methods	Randomised control trial
Participants	Children aged 4–6 years
Interventions	Computer‐based cognitive training
Outcomes	Neurodevelopmental assessments
Starting date	NA
Contact information	Mr Jaco Louw, 42 Bloemhof Road, Belville, Cape Town 7535, South Africa
Notes	
Patrenko (2017)	
Study name	Development and evaluation of an evidence‐based mobile health caregiver intervention for FASD
Methods	Effectiveness trial
Participants	Caregivers and children aged 3–12
Interventions	Families moving forward connect application
Outcomes	Implementation outcomes
Starting date	
Contact information	Christie Petrenko: christie_petrenko@urmc. rochester.edu University of Rochester
Notes	
Petrenko et al. (2017)	
Study name	A Study of Parent and Child Emotions in Foetal Alcohol Spectrum Disorder
Methods	Randomized trial
Participants	Children with FASD and caregivers
Interventions	Tuning in to Kids
Outcomes	Multiple measures of parental emotional experience (e.g., awareness/acceptance), child RSA, child emotional regulation
Starting date	August 13, 2018
Contact information	Christie Petrenko: christie_petrenko@urmc. rochester.edu
Notes	
University of Alberta (2018)	
Study name	Self‐regulation in adolescents with FASD: the efficacy of a targeted intervention
Methods	Non‐randomised trial
Participants	Adolescents (11–17 years) with FASD
Interventions	Alert program
Outcomes	d‐KEFS, Iowa gambling test, Whack a mole test, monetary choice questionnaire, dot probe task, Ray complex figure task, BRIEF, Adolescent self‐regulatory inventory, ABAS‐2, CBCL, health habit questionnaire, paediatric sleep questionnaire, fingernail cortisol
Starting date	September 2016
Contact information	University of Alberta
Notes	
Wozniack (2017)	
Study name	Cognitive Training and tDCS for Children With FASD
Methods	Randomized control trial
Participants	Children and adolescents with PAE (10–16 years)
Interventions	Cognitive remediation training
Outcomes	Brain HQ learning rate, d‐KEFS, Flanker task
Starting date	NA
Contact information	Jeffrey Wozniak, University of Minnesota
Notes	

**Data and analyses**

**Visual attention (direct)**

**Table:** 

**Outcome or subgroup title**	**No. of studies**	**No. of participants**	**Statistical method**	**Effect size**
1.1 Visual attention (direct)	2	32	Std. mean difference (IV, random, 95% CI)	0.90 [−1.41, 3.21]

**Auditory attention (direct)**

**Table:** 

**Outcome or subgroup title**	**No. of studies**	**No. of participants**	**Statistical method**	**Effect size**
2.1 Auditory attention (direct, ICC = 0.00)	3	62	Std. mean difference (IV, Random, 95% CI)	0.06 [−1.06, 1.18]
2.2 Auditory attention (direct, ICC = 0.02)	3	62	Std. mean difference (IV, random, 95% CI)	0.06 [−1.09, 1.21]
2.3 Auditory attention (direct, ICC = 0.03)	3	62	Std. mean difference (IV, random, 95% CI)	0.06 [−1.10, 1.22]
2.4 Auditory attention (direct, ICC = 0.1)	3	62	Std. mean difference (IV, random, 95% CI)	0.06 [−1.18, 1.31]

**Cognitive flexibility (direct)**

**Table:** 

**Outcome or subgroup title**	**No. of studies**	**No. of participants**	**Statistical method**	**Effect size**
3.1 Cognitive flexibility (direct, ICC = 0.00)	2	45	Std. mean difference (IV, random, 95% CI)	0.23 [−0.40, 0.86]
3.2 Cognitive flexibility (direct, ICC = 0.02)	2	45	Std. mean difference (IV, random, 95% CI)	0.25 [−0.41, 0.91]
3.3 Cognitive flexibility (direct, ICC = 0.03)	2	45	Std. mean difference (IV, random, 95% CI)	0.26 [−0.41, 0.93]
3.4 Cognitive flexibility (direct, ICC = 0.1)	2	45	Std. mean difference (IV, random, 95% CI)	0.31 [−0.41, 1.03]

**Attentional inhibition (direct)**

**Table:** 

**Outcome or subgroup title**	**No. of studies**	**No. of participants**	**Statistical method**	**Effect size**
4.1 Attentional inhibition (direct, ICC = 0.00)	2	45	Std. mean difference (IV, random, 95% CI)	0.04 [−0.58, 0.65]
4.2 Attentional inhibition (direct, ICC = 0.02)	2	45	Std. mean difference (IV, random, 95% CI)	0.06 [−0.57, 0.69]
4.3 Attentional inhibition (direct, ICC = 0.03)	2	45	Std. mean difference (IV, random, 95% CI)	0.07 [−0.56, 0.70]
4.4 Attentional inhibition (direct, ICC = 0.1)	2	45	Std. mean difference (IV, random, 95% CI)	0.13 [−0.55, 0.82]

**Response inhibition (direct)**

**Table:** 

**Outcome or subgroup title**	**No. of studies**	**No. of participants**	**Statistical method**	**Effect size**
5.1 Response inhibition (direct, ICC = 0.00)	3	62	Std. mean difference (IV, random, 95% CI)	0.47 [−0.04, 0.99]
5.2 Response inhibition (direct, ICC = 0.02)	3	62	Std. mean difference (IV, random, 95% CI)	0.48 [−0.05, 1.01]
5.3 Response inhibition (direct, ICC = 0.03)	3	62	Std. mean difference (IV, random, 95% CI)	0.49 [−0.05, 1.02]
5.4 Response inhibition (direct, ICC = 0.1)	3	62	Std. mean difference (IV, random, 95% CI)	0.50 [−0.07, 1.08]

**Global executive function (indirect)**

**Table:** 

**Outcome or subgroup title**	**No. of studies**	**No. of participants**	**Statistical method**	**Effect size**
7.1 BRIEF—Global Executive Composite	2	42	Std. mean difference (IV, random, 95% CI)	0.21 [−0.40, 0.82]
7.2 Global Executive Composite (indirect, ICC = 0.02)	2	42	Std. mean difference (IV, random, 95% CI)	0.21 [−0.43, 0.85]
7.3 Global Executive Composite (indirect, ICC = 0.03)	2	42	Std. mean difference (IV, random, 95% CI)	0.21 [−0.44, 0.86]
7.4 Global Executive Composite (indirect, ICC = 0.1)	2	42	Std. mean difference (IV, random, 95% CI)	0.20 [−0.52, 0.92]

**Behavioural regulation (indirect)**

**Table:** 

**Outcome or subgroup title**	**No. of studies**	**No. of participants**	**Statistical method**	**Effect size**
9.1 Behavioural regulation (indirect)	2	42	Std. mean difference (IV, random, 95% CI)	0.18 [−0.43, 0.79]
9.2 Behavioural regulation (indirect, ICC = 0.02)	2	42	Std. mean difference (IV, random, 95% CI)	0.17 [−0.46, 0.80]
9.3 Behavioural regulation (indirect, ICC = 0.03)	2	42	Std. mean difference (IV, random, 95% CI)	0.16 [−0.48, 0.81]
9.4 Behavioural regulation (indirect, ICC = 0.1)	2	42	Std. mean difference (IV, random, 95% CI)	0.14 [−0.58, 0.85]

**Emotional control (indirect)**

**Table:** 

**Outcome or subgroup title**	**No. of studies**	**No. of participants**	**Statistical method**	**Effect size**
11.1 Emotional control (indirect)	3	130	Std. mean difference (IV, random, 95% CI)	0.01 [−0.33, 0.36]
11.2 Emotional control (indirect, ICC = 0.02)	3	130	Std. mean difference (IV, random, 95% CI)	0.00 [−0.35, 0.35]
11.3 Emotional control (indirect, ICC = 0.03)	3	130	Std. mean difference (IV, random, 95% CI)	−0.00 [−0.36, 0.35]
11.4 Emotional control (indirect, ICC = 0.1)	3	130	Std. mean difference (IV, random, 95% CI)	−0.02 [−0.39, 0.35]

**Inhibition (indirect)**

**Table:** 

**Outcome or subgroup title**	**No. of studies**	**No. of participants**	**Statistical method**	**Effect size**
12.1 Inhibition (indirect)	2	103	Std. mean difference (IV, random, 95% CI)	−0.08 [−0.47, 0.31]
12.2 Inhibition (indirect, ICC = 0.02)	2	103	Std. mean difference (IV, random, 95% CI)	−0.09 [−0.48, 0.31]
12.3 Inhibition (indirect, ICC = 0.03)	2	103	Std. mean difference (IV, random, 95% CI)	−0.09 [−0.49, 0.30]
12.4 Inhibition (indirect, ICC = 0.1)	2	103	Std. mean difference (IV, random, 95% CI)	−0.11 [−0.52, 0.30]

**Shifting (indirect)**

**Table:** 

**Outcome or subgroup title**	**No. of studies**	**No. of participants**	**Statistical method**	**Effect size**
13.1 Shifting (indirect)	2	103	Std. mean difference (IV, random, 95% CI)	0.04 [−0.35, 0.43]
13.2 Shifting (indirect, ICC = 0.02)	2	103	Std. mean difference (IV, random, 95% CI)	0.04 [−0.36, 0.43]
13.3 Shifting (indirect, ICC = 0.03)	2	103	Std. mean difference (IV, random, 95% CI)	0.04 [−0.36, 0.43]
13.4 Shifting (indirect, ICC = 0.1)	2	103	Std. mean difference (IV, random, 95% CI)	0.03 [−0.38, 0.45]

## SOURCES OF SUPPORT

**Internal sources**

- PhD Scholarship, Australia
  Paid to the lead author as part of grant: Drug and Alcohol Program: Foetal Alcohol Spectrum Disorder (FASD) Diagnostic Services and Models of Care Grant Opportunity—H1617G038.

**External sources**

- Drug and Alcohol Program: Foetal Alcohol Spectrum Disorder (FASD) Diagnostic Services and Models of Care Grant Opportunity—H1617G038, Commonwealth Government, Australia

## Supporting information

Supporting information.
