# Supplementary material for: Interventions for improving executive functions in children with foetal alcohol spectrum disorder (FASD): A systematic review
Source: Campbell Syst Rev. 2022 Nov 3;18(4):e1258. doi: 10.1002/cl2.1258 (PMC9634003; doi:10.1002/cl2.1258)
Supplement: Supplementary file 1 — Supporting information. [file CL2-18-e1258-s001.docx]

# Appendices

## 1 Search Record

**Generic Search Structure**

((executive* N/3 (function* OR control* OR atten*)) OR (self N/3 regulat*) OR "effortful control" OR "working memory" OR (cognitive N/3 flexib*) OR (emotion* N/3 (regulat* OR inhibit*)) OR "set shift*" OR reasoning OR planning OR attention OR inhibit*)

AND

((alcohol* N/3 (prenatal* OR fetal OR foetal OR fetus* OR foetus* OR "neurodevelopment disorder*" OR "birth defect" OR "spectrum disorder*") OR "sentinel facial feature*")

AND

(RCT OR randomi* OR trial* OR experiment* OR interven* OR therap* OR treat* OR program* OR review* OR "meta-analy*" OR "meta analy*")

**ELECTRONIC DATABASES**

**Campbell Collaboration Library (Wiley Journal Website)**

Dates: 24/08/2018, 03/07/2020, 20/12/2021 (search results limited up to 31/12/2020)

Syntax (≤2019): "fetal alcohol" OR "prenatal alcohol" OR "executive functions"

Syntax (≥2019): alcohol*

**Cochrane Collaboration Library (including CENTRAL)**

Dates: 22/08/2018, 03/07/2020, 20/12/2021 (search results limited up to 31/12/2020)

Syntax:

1. executive* NEAR/3 (function* OR control* OR atten*)
2. self NEAR/3 regulat*
3. emotion* NEAR/3 (regulat* OR inhibit*)
4. cognitive NEAR/3 flexib*
5. "effortful control" OR "working memory" OR "set shift*" OR reasoning OR planning OR attention OR inhibit*
6. #1 OR #2 OR #3 OR #4 OR #5
7. alcohol* NEAR/3 (prenatal* OR fetal OR foetal OR fetus* OR foetus* OR "neurodevelopment disorder*" OR "birth defect" OR "spectrum disorder*")
8. "sentinel facial feature*"
9. MeSH descriptor: [Fetal Alcohol Spectrum Disorders] this term only
10. #7 OR #8 OR #9
11. RCT OR randomi* OR trial* OR experiment* OR interven* OR therap* OR treat* OR program* OR review* OR "meta-analy*" OR "meta analy*"
12. #6 AND #10 AND #11

**CINAHL (EBSCOhost)**

Dates: 22/08/2018, 03/07/2020, 20/12/2021 (search results limited up to 31/12/2020)

Syntax:

1. TI ( ((executive* N3 (function* OR control* OR atten*)) ) OR AB ( ((executive* N3 (function* OR control* OR atten*)) ) OR SU ( ((executive* N3 (function* OR control* OR atten*)) )
2. TI (self N3 regulat*) OR AB (self N3 regulat*) OR SU (self N3 regulat*)
3. TI ( (emotion* N3 (regulat* OR inhibit*)) ) OR AB ( (emotion* N3 (regulat* OR inhibit*)) ) OR SU ( (emotion* N3 (regulat* OR inhibit*)) )
4. TI (cognitive N3 flexib*) OR AB (cognitive N3 flexib*) OR SU (cognitive N3 flexib*)
5. TI ( (“effortful control” OR “working memory” OR “set shift*” OR reasoning OR planning OR attention OR inhibit*) ) OR AB ( (“effortful control” OR “working memory” OR “set shift*” OR reasoning OR planning OR attention OR inhibit*) ) OR SU ( (“effortful control” OR “working memory” OR “set shift*” OR reasoning OR planning OR attention OR inhibit*) )
6. S1 OR S2 OR S3 OR S4 OR S5
7. TI ( ((alcohol* N3 (prenatal* OR fetal OR foetal OR fetus* OR foetus* OR “neurodevelopment disorder*” OR “birth defect” OR “spectrum disorder*”) ) OR AB ( ((alcohol* N3 (prenatal* OR fetal OR foetal OR fetus* OR foetus* OR “neurodevelopment disorder*” OR “birth defect” OR “spectrum disorder*”) ) OR SU ( ((alcohol* N3 (prenatal* OR fetal OR foetal OR fetus* OR foetus* OR “neurodevelopment disorder*” OR “birth defect” OR “spectrum disorder*”) )
8. TI (“sentinel facial feature*”) OR AB (“sentinel facial feature*”) OR SU (“sentinel facial feature*”)
9. S7 OR S8
10. TI ( (RCT OR randomi* OR trial* OR experiment* OR interven* OR therap* OR treat* OR program* OR review* OR “meta-analy*” OR “meta analy*”) ) OR AB ( (RCT OR randomi* OR trial* OR experiment* OR interven* OR therap* OR treat* OR program* OR review* OR “meta-analy*” OR “meta analy*”) ) OR SU ( (RCT OR randomi* OR trial* OR experiment* OR interven* OR therap* OR treat* OR program* OR review* OR “meta-analy*” OR “meta analy*”) )
11. S6 AND S9 AND S10

**Embase (Elseiver)**

Dates: 22/08/2018, 03/07/2020, 20/12/2021 (search results limited up to 31/12/2020)

Syntax:

1. (executive* NEAR/3 (function* OR control* OR atten*)):ti,ab,kw
2. (emotion* NEAR/3 (regulat* OR inhibit*)):ti,ab,kw
3. (self NEAR/3 regulat*):ti,ab,kw
4. (cognitive NEAR/3 flexib*):ti,ab,kw
5. 'effortful control':ti,ab,kw OR 'working memory':ti,ab,kw OR 'set shift*':ti,ab,kw OR 'reasoning':ti,ab,kw OR 'planning':ti,ab,kw OR 'attention':ti,ab,kw OR 'inhibit*':ti,ab,kw
6. #1 OR #2 OR #3 OR #4 OR #5
7. (alcohol* NEAR/3 (prenatal* OR fetal OR foetal OR fetus* OR foetus* OR 'neurodevelopment disorder*' OR 'birth defect' OR 'spectrum disorder*')):ti,ab,kw
8. 'sentinel facial feature*':ti,ab,kw
9. 'fetal alcohol syndrome'
10. #7 OR #8 OR #9
11. rct:ti,ab,kw OR randomi*:ti,ab,kw OR trial*:ti,ab,kw OR experiment*:ti,ab,kw OR interven*:ti,ab,kw OR therap*:ti,ab,kw OR treat*:ti,ab,kw OR program*:ti,ab,kw OR review*:ti,ab,kw OR 'meta-analy*':ti,ab,kw OR 'meta analy*':ti,ab,kw
12. #6 AND #10 AND #11
13. #12 AND [humans]/lim

**ProQuest (Dissertations & Theses Global)**

Dates: 10/08/2018, 03/07/2020, 20/12/2021 (search results limited up to 31/12/2020)

Syntax: (noft((executive* NEAR/3 (function* OR control* OR atten*))) OR noft((self NEAR/3 regulat*)) OR noft((cognitive NEAR/3 flexib*)) OR noft((emotion* NEAR/3 (regulat* OR inhibit*))) OR noft(("effortful control" OR "working memory" OR "set shift*" OR reasoning OR planning OR attention OR inhibit*))) AND (noft((alcohol* NEAR/3 (prenatal* OR embry* OR fetal OR foetal OR fetus* OR foetus* OR "neurodevelopment disorder*" OR "birth defect" OR "spectrum disorder*"))) OR noft("sentinel facial feature*")) AND noft((RCT OR randomi* OR trial* OR experiment* OR interven* OR therap* OR treat* OR program* OR review* OR "meta-analy*" OR "meta analy*"))

**ProQuest (Family Health)**

Dates: 22/08/2018, 03/07/2020 (search results limited up to 31/12/2019); no longer available for 2020 top-up search

Syntax: (noft((executive* NEAR/3 (function* OR control* OR atten*))) OR noft((self NEAR/3 regulat*)) OR noft((cognitive NEAR/3 flexib*)) OR noft((emotion* NEAR/3 (regulat* OR inhibit*))) OR noft(("effortful control" OR "working memory" OR "set shift*" OR reasoning OR planning OR attention OR inhibit*))) AND (noft((alcohol* NEAR/3 (prenatal* OR embry* OR fetal OR foetal OR fetus* OR foetus* OR "neurodevelopment disorder*" OR "birth defect" OR "spectrum disorder*"))) OR noft("sentinel facial feature*")) AND noft((RCT OR randomi* OR trial* OR experiment* OR interven* OR therap* OR treat* OR program* OR review* OR "meta-analy*" OR "meta analy*"))

**ProQuest (Health & Medical Complete)**

Dates: 22/08/2018, 03/07/2020, 20/12/2021 (search results limited up to 31/12/2020)

Syntax: (noft((executive* NEAR/3 (function* OR control* OR atten*))) OR noft((self NEAR/3 regulat*)) OR noft((cognitive NEAR/3 flexib*)) OR noft((emotion* NEAR/3 (regulat* OR inhibit*))) OR noft(("effortful control" OR "working memory" OR "set shift*" OR reasoning OR planning OR attention OR inhibit*))) AND (noft((alcohol* NEAR/3 (prenatal* OR embry* OR fetal OR foetal OR fetus* OR foetus* OR "neurodevelopment disorder*" OR "birth defect" OR "spectrum disorder*"))) OR noft("sentinel facial feature*")) AND noft((RCT OR randomi* OR trial* OR experiment* OR interven* OR therap* OR treat* OR program* OR review* OR "meta-analy*" OR "meta analy*"))

**ProQuest (Nursing & Allied Health)**

Dates: 22/08/2018, 03/07/2020, 20/12/2021 (search results limited up to 31/12/2020)

Syntax: (noft((executive* NEAR/3 (function* OR control* OR atten*))) OR noft((self NEAR/3 regulat*)) OR noft((cognitive NEAR/3 flexib*)) OR noft((emotion* NEAR/3 (regulat* OR inhibit*))) OR noft(("effortful control" OR "working memory" OR "set shift*" OR reasoning OR planning OR attention OR inhibit*))) AND (noft((alcohol* NEAR/3 (prenatal* OR embry* OR fetal OR foetal OR fetus* OR foetus* OR "neurodevelopment disorder*" OR "birth defect" OR "spectrum disorder*"))) OR noft("sentinel facial feature*")) AND noft((RCT OR randomi* OR trial* OR experiment* OR interven* OR therap* OR treat* OR program* OR review* OR "meta-analy*" OR "meta analy*"))

**ProQuest (Psychology Journals)**

Dates: 22/08/2018, 03/07/2020, 20/12/2021 (search results limited up to 31/12/2020)

Syntax: (noft((executive* NEAR/3 (function* OR control* OR atten*))) OR noft((self NEAR/3 regulat*)) OR noft((cognitive NEAR/3 flexib*)) OR noft((emotion* NEAR/3 (regulat* OR inhibit*))) OR noft(("effortful control" OR "working memory" OR "set shift*" OR reasoning OR planning OR attention OR inhibit*))) AND (noft((alcohol* NEAR/3 (prenatal* OR embry* OR fetal OR foetal OR fetus* OR foetus* OR "neurodevelopment disorder*" OR "birth defect" OR "spectrum disorder*"))) OR noft("sentinel facial feature*")) AND noft((RCT OR randomi* OR trial* OR experiment* OR interven* OR therap* OR treat* OR program* OR review* OR "meta-analy*" OR "meta analy*"))

**ProQuest (Social Services Abstracts)**

Dates: 22/08/2018, 03/07/2020, 20/12/2021 (search results limited up to 31/12/2020)

Syntax: (noft((executive* NEAR/3 (function* OR control* OR atten*))) OR noft((self NEAR/3 regulat*)) OR noft((cognitive NEAR/3 flexib*)) OR noft((emotion* NEAR/3 (regulat* OR inhibit*))) OR noft(("effortful control" OR "working memory" OR "set shift*" OR reasoning OR planning OR attention OR inhibit*))) AND (noft((alcohol* NEAR/3 (prenatal* OR embry* OR fetal OR foetal OR fetus* OR foetus* OR "neurodevelopment disorder*" OR "birth defect" OR "spectrum disorder*"))) OR noft("sentinel facial feature*")) AND noft((RCT OR randomi* OR trial* OR experiment* OR interven* OR therap* OR treat* OR program* OR review* OR "meta-analy*" OR "meta analy*"))

**PsycEXTRA (OvidSp)**

Dates: 20/08/2018, 03/07/2020, 20/12/2021 (search results limited up to 31/12/2020)

Syntax:

1. (executive* adj3 (function* or control* or atten*)).ab,hw,id,ot,ti.
2. (self adj3 regulat*).ab,hw,id,ot,ti.
3. (emotion* adj3 (regulat* or inhibit*)).ab,hw,id,ot,ti.
4. (cognitive adj3 flexib*).ab,hw,id,ot,ti.
5. ("effortful control" or "working memory" or "set shift*" or reasoning or planning or attention or inhibit*).ab,hw,id,ot,ti.
6. 1 or 2 or 3 or 4 or 5
7. (alcohol* adj3 (prenatal* or fetal or foetal or fetus* or foetus* or "neurodevelopment disorder*" or "birth defect" or "spectrum disorder*")).ab,hw,id,ot,ti.
8. "sentinel facial feature*".ab,hw,id,ot,ti.
9. fetal alcohol spectrum disorders.mh.
10. 7 or 8 or 9
11. (RCT or randomi* or trial* or experiment* or interven* or therap* or treat* or program* or review* or "meta-analy*" or "meta analy*").ab,hw,id,ot,ti.
12. 6 and 10 and 11

**PsycINFO (OvidSp)**

Dates: 20/08/2018, 03/07/2020, 20/12/2021 (search results limited up to 31/12/2020)

Syntax:

1. (executive* adj3 (function* or control* or atten*)).ab,hw,id,ot,ti.
2. (self adj3 regulat*).ab,hw,id,ot,ti.
3. (emotion* adj3 (regulat* or inhibit*)).ab,hw,id,ot,ti.
4. (cognitive adj3 flexib*).ab,hw,id,ot,ti.
5. ("effortful control" or "working memory" or "set shift*" or reasoning or planning or attention or inhibit*).ab,hw,id,ot,ti.
6. 1 or 2 or 3 or 4 or 5
7. (alcohol* adj3 (prenatal* or fetal or foetal or fetus* or foetus* or "neurodevelopment disorder*" or "birth defect" or "spectrum disorder*")).ab,hw,id,ot,ti.
8. "sentinel facial feature*".ab,hw,id,ot,ti.
9. fetal alcohol spectrum disorders.mh.
10. 7 or 8 or 9
11. (RCT or randomi* or trial* or experiment* or interven* or therap* or treat* or program* or review* or "meta-analy*" or "meta analy*").ab,hw,id,ot,ti.
12. 6 and 10 and 11

**PubMed**

Date: 23/08/2018, 03/07/2020, 20/12/2021 (search results limited up to 31/12/2020)

Syntax:

1. "executive function*"[Title/Abstract] OR "executive control [Title/Abstract] OR "executive attention"[Title/Abstract] OR "self regulat*"[Title/Abstract] OR "emotional control"[Title/Abstract] OR "emotion regulation"[Title/Abstract] OR "emotional regulation" [Title/Abstract] OR "emotion inhibit*"[Title/Abstract] OR "cognitive flexibility"[Title/Abstract] OR "effortful control"[Title/Abstract] OR "working memory"[Title/Abstract] OR "set shift*"[Title/Abstract] OR "reasoning"[Title/Abstract] OR "planning"[Title/Abstract] OR "attention"[Title/Abstract] OR "inhibit*"[Title/Abstract]
2. "Prenatal alcohol exposure"[Title/Abstract] OR "fetal alcohol syndrome*"[Title/Abstract] OR "fetal alcohol spectrum disorder*"[Title/Abstract] OR "foetal alcohol syndrome*"[Title/Abstract] OR "foetal alcohol spectrum disorder*"[Title/Abstract] OR "alcohol birth defect*"[Title/Abstract] OR "alcohol related neurodevelopmental disorder*"[Title/Abstract] OR "sentinel facial feature*"[Title/Abstract]
3. "fetal alcohol spectrum disorders"[MeSH Major Topic]
4. #2 OR #3
5. RCT[Title/Abstract] OR randomi*[Title/Abstract] OR trial*[Title/Abstract] OR experiment*[Title/Abstract] OR interven*[Title/Abstract] OR therap*[Title/Abstract] OR treat*[Title/Abstract] OR program*[Title/Abstract] OR review*[Title/Abstract] OR meta-analy*[Title/Abstract] OR "meta analy*"[Title/Abstract])
6. 1 AND 4 AND 5

**Scopus**

Date: 20/08/2018, 03/07/2020, 20/12/2021 (search results limited up to 31/12/2020)

Syntax:

1. TITLE-ABS-KEY ( executive* W/3 ( function* OR control* OR atten* ) )
2. TITLE-ABS-KEY ( self W/3 regulat* )
3. TITLE-ABS-KEY ( cognitive W/3 flexib* )
4. TITLE-ABS-KEY ( emotion* W/3 ( regulat* OR inhibit* ) )
5. TITLE-ABS-KEY ( "effortful control" OR "working memory" OR "set shift*" OR reasoning OR planning OR attention OR inhibit* )
6. 1 OR 2 OR 3 OR 4 OR 5
7. TITLE-ABS-KEY ( alcohol* W/3 ( prenatal* OR fetal OR foetal OR fetus* OR foetus* OR "neurodevelopment disorder*" OR "birth defect" OR "spectrum disorder*" ) )
8. TITLE-ABS-KEY ( "sentinel facial feature*" )
9. 7 OR 8
10. TITLE-ABS-KEY ( rct OR randomi* OR trial* OR experiment* OR interven* OR therap* OR treat* OR program* OR review* OR "meta-analy*" OR "meta-analy*" )
11. 6 AND 9 AND 10

*Searches ≤2018 excluded non-English articles prior to export. Searches ≥2019 did not exlcude non-English articles prior to export. To exclude the large amount of non-research articles and animal research, the final top-up search used the following restrictions prior to export: AND ( LIMIT-TO ( DOCTYPE , "ar" ) OR LIMIT-TO ( DOCTYPE , "re" ) OR LIMIT-TO ( DOCTYPE , "ch" ) OR LIMIT-TO ( DOCTYPE , "cp" ) OR LIMIT-TO ( DOCTYPE , "bk" ) ) AND ( EXCLUDE ( EXACTKEYWORD , "nonhuman" ) OR EXCLUDE ( EXACTKEYWORD , "animal" ) OR EXCLUDE ( EXACTKEYWORD , "animals" ) OR EXCLUDE ( EXACTKEYWORD , "animal tissue" ) OR EXCLUDE ( EXACTKEYWORD , "mouse" ) OR EXCLUDE ( EXACTKEYWORD , "rat" ) OR EXCLUDE ( EXACTKEYWORD , "mice" ) OR EXCLUDE ( EXACTKEYWORD , "rats" ) OR EXCLUDE ( EXACTKEYWORD , "disease models, animal" ) OR EXCLUDE ( EXACTKEYWORD , "rats, wistar" ) ).

**Web of Science (Core Collection*)**

Date: 22/08/2018, 03/07/2020, 20/12/2021 (search results limited up to 31/12/2020)

Syntax:

1. TOPIC: ((executive* NEAR/3 (function* OR control* OR atten*)))
2. TOPIC: ((self NEAR/3 regulat*))
3. TOPIC: ((cognitive NEAR/3 flexib*))
4. TOPIC: ((emotion* NEAR/3 (regulat* OR inhibit*)))
5. (("effortful control" OR "working memory" OR "set shift*" OR reasoning OR planning OR attention OR inhibit*))
6. #5 OR #4 OR #3 OR #2 OR #1
7. TOPIC: (alcohol* NEAR/3 (prenatal* OR embry* OR fetal OR foetal OR fetus* OR foetus* OR "neurodevelopment disorder*" OR "birth defect" OR "spectrum disorder*"))
8. TOPIC: ("sentinel facial feature*")
9. #8 OR #7
10. TOPIC: (RCT OR randomi* OR trial* OR experiment* OR interven* OR therap* OR treat* OR program* OR review* OR "meta-analy*" OR "meta analy*")
11. #10 AND #9 AND #6

*Indexes=SCI-EXPANDED, SSCI, A&HCI, CPCI-S, CPCI-SSH, BKCI-S, BKCI-SSH, ESCI, CCR-EXPANDED, IC Timespan=1973-2020.

**GREY LITERATURE, CONFERENCE PROCEEDINGS, TRIAL REGISTERS**

| **Grey Literature Source** | **Date** | **Search Approach** |
| --- | --- | --- |
| Alcohol and Alcohol Science Database (ETOH) | - | No longer exists to search |
| Alcohol Concern (UK) | 23/08/2018; 20/12/2021 | 2018: Examined all research reports (*n* = 33), none about FASD/PAE.  2021: Now Alcohol Change: https://alcoholchange.org.uk/. Examined all publications in Research Hub between 2018 and 2020 (*n* = 19). None were about FASD or prenatal alcohol exposure. |
| Alcohol and Drug Foundation (Australia) | 23/08/2018; 20/12/2021 | 2018: As per generic search string (*n* = 34), 4 related to FASD/PAE and imported to EndNote.  2021: Used the following terms in the ADF library (low search functionality and unclear if wild cards work): ab:(fetal OR prenatal) AND ab:(alcohol) AND ab:(RCT OR randomised OR randomisation OR trial OR trials OR trialled OR experiment OR experimental OR intervention OR interventions OR therapy OR therapies OR treatment OR treated OR treating OR treated OR program OR programs OR review OR reviews OR meta-analysis OR meta-analyses). Also searched under ‘Publications’, none were about FASD or prenatal alcohol exposure. |
| National Institute on Alcohol Abuse and Alcoholism | 23/08/2018; 20/12/2021 | 2018: No search function, found 25 articles in their FASD journal (https://www.arcr.niaaa.nih.gov/topics.htm), 3 about FASD/PAE and imported into EndNote.  2021: No search function, screened their 2019 and 2020 open access journal. One mentioned FASD, but no interventions evaluated so not imported into EndNote. |
| Foundation for Alcohol Research and Education (FARE) | 23/08/2018; 20/12/2021 | 2018: No complex search functionality, searched "fetal alcohol" in search bar, located 163 records. None about FASD/PAE.  2021: Under ‘Research papers’ used ‘fetal’ in the search bar. Seventeen results and none between 2019 – 2021. |
| The Alcohol Pharmacology Education Partnership | 23/08/2018; 20/12/2021 | No research papers indexed on website. |
| Australian Centre for Child Protection | 23/08/2018; 20/12/2021 | 2018: No research papers indexed on website.  2021: All publications screened for relevancy. Three in 2020 related to FASD, but were not evaluations of interventions, so were not exported for screening in SysReview. Two in 2014 related to FASD (see below). The first one provides a narrative review of potentially eligible impact evaluations and these were already identified by the systematic search. Two records related to FASD in 2013, but these were not impact evaluations of eligible interventions. None imported to EndNote. |
| Royal Australasian College of Surgeons | 23/08/2018; 20/12/2021 | No complex search functionality, searched "fetal alcohol", no records found. |
| Society for Research on Child Development (SCRD) | 23/08/2018; 20/12/2021 | 2018: No complex search functionality, searched alcohol and screened all 20 results, none about FASD/PAE.  2021: No complex search functionality, searched alcohol and screened 2019 - 2020 results, not about FASD/PAE. |
| Social Science Research Network | 23/08/2018; 20/12/2021 | 2018: No complex search functionality, searched "fetal alcohol" and located 54 records, none about FASD/PAE.  2021: SNo complex search functionality, searched "fetal alcohol", which returned 16 results. Those in the update period were not relevant for the review. |
| Australian Research Alliance for Children and Youth | 23/08/2018; 20/12/2021 | 2018: Scanned publications and resources page, no publications were about FASD/PAE.  2021: Searched publications and research library, using the word 'fetal’. None were relevant to the review. |
| NoFASD Australia | 23/08/2018; 20/12/2021 | Research linked to FASDHub Australia. |
| FASDHub Australia | 23/08/2018; 20/12/2021 | 2018: Located 150 publications, 4 deemed eligible using full-text screening criteria. However 3 were known duplicates already captured. One record imported into EndNote.  2021: As of 20/12/21, there were 297 publications. Sorted on year and examined all titles published between 2018 and 2021. None were impact evaluations of eligible interventions. |
| NoFAS.org | 23/08/2018; 20/12/2021 | Examined entire website, no publications or research located. |
| Russell Family Fetal Alcohol Disorders Association (RFFADA) | 23/08/2018; 20/12/2021 | Examined entire website, no publications or research located. |
| CanFASD (Canada FASD Research Network) | 23/08/2018; 20/12/2021 | 2018: Four potentially eligible documents located in resources/research library. However, 2 were known duplicates already captured. Two records imported into EndNote.  2021: Examined the annotated bibliographies for 2016 – 2020 ( https://canfasd.ca/topics/top-papers/). All but one were either already in the review or captured by the systematic search, or not eligible. |
| FASD outreach (British Columbia) | 23/08/2018; 20/12/2021 | 2018: One potentially eligible document located in resources/research library. However, this was a known duplicate already captured. No records imported into EndNote.  2021: Examined publications under ‘Resources’, but these seemed to be focused on practice recommendations rather than empirical research or evaluations of eligible interventions (https://www.fasdoutreach.ca/resources/). Also examined records under the Executive Function heading and found no evaluations of interventions: https://www.fasdoutreach.ca/resources/topics/executive-function. |
| Fetal Alcohol Network NZ | 23/08/2018; 20/12/2021 | Examined entire website, no publications or research located. |

| **Conference Proceedings** | **Date** | **Search Approach** |
| --- | --- | --- |
| Australasian FASD Conference | 24/08/2018; 20/12/2021 | Handsearched publication and resources page, no documents fit eligiblity criteria. No conferences have taken place since 2018 harvest. |
| International Research Conference on Adolescents and Adults with FASD | 24/08/2018; 20/12/2021 | Handsearched conference presentation section, no documents fit eligiblity criteria. No conferences have taken place since 2018 harvest. |
| International Conference on FASD | 24/08/2018; 20/12/2021 | 2018: Examined all program documents on the following website: https://interprofessional.ubc.ca/fasd-conferences/. No unique records fit eligibility criteria.  2021: Examined program document for 2019 (most recent publication). |

| **Trial Register / Handsearch Source** | **Date** | **Search Approach** |
| --- | --- | --- |
| Australia and New Zealand Clinical Trials Registry | 24/08/2018 | Limited search functionality, ran three truncated searches:   - executive function* AND ("fetal alcohol" OR FASD) - "fetal alcohol" OR FASD OR FAS OR "prenatal alcohol" - (FASD OR "fetal alcohol") AND intervention   *Not updated in 2021, as this source is captured by CENTRAL. |
| ClinicalTrials.gov | 24/08/2018 | Limited search functionality, ran three truncated searches:   - executive function* AND ("fetal alcohol" OR FASD) - "fetal alcohol" OR FASD OR FAS OR "prenatal alcohol" - (FASD OR "fetal alcohol") AND intervention   *Not updated in 2021, as this source is captured by CENTRAL. |
| Clinical Trials Results | 24/08/2018 | Limited search functionality. Handsearched by topic.  *Not updated in 2021, as this source is captured by CENTRAL. |
| Cochrane Central Register of Controlled Trials (CENTRAL) | 24/08/2018 | Syntax: 'executive function in Title Abstract Keyword AND fetal alcohol spectrum disorder in Title Abstract Keyword - (Word variations have been searched).  *Search equivalent to Cochrane search for 2021 |
| ISRCTN Registry (controlled-trials.com) | 24/08/2018 | Limited search functionality, ran two truncated searches:   - "executive function*" AND "fetal alcohol" - "fetal alcohol" AND intervention   *Not updated in 2021, as this source is captured by CENTRAL. |
| NIH RePORTER | 24/08/2018;  22/12/2021 | Limited search functionality, ran two truncated searches:   - "fetal alcohol" AND intervention / "foetal alcohol" AND intervention - "fetal alcohol" AND "executive function*" / "foetal alcohol" AND "executive function*" |
| Trials Register of Health Interventions (TRoPHI) | 24/08/2018;  22/12/2021 | 1. Freetext (All but Authors): "Prenatal alcohol" OR "fetal alcohol" OR "foetal alcohol" OR "alcohol birth defect*" OR "alcohol related neurodevelopmental disorder*" 2. Freetext (All but Authors): "executive function*" OR "executive control" OR "self regulat*" OR "emotion* regulation" 3. 1 AND 2 |
| UK Clinical Research Network (UKCRN Study Portfolio) | 24/08/2018 | Handsearched publications section of website. |
| WHO International Clinical Trials Registry | - | Captured by Cochrane Collaboration search (CENTRAL). |
| Alcoholism: Clinical and Experimental Research  Applied Neuropsychology: Child  Child Neuropsychology  Developmental Neurorehabilitation  Research in Developmental Disabilities | 24/08/2018; 03/07/2020 | Used generic search on journal titles in Web of Science, limited to 2018 - 2019. All results imported into EndNote for processing. |

## 2 Screening Form

| 1. Study ID#: _ _ _ |
| --- |
| 2. Screening date: _ _-_ _-_ _ _ _ |
| 3. First author: ______________________  4. Document is a duplicate:  □ No  □ Unsure  □ **Yes (EXCLUDE)**  5. Document type:  □ Journal article  □ Book  □ Book chapter  □ Editorial  □ **None (EXCLUDE)** |
| 6. Year of publication:  □ After 1972  □ **1972 or earlier (EXCLUDE)**  7. Sample age:  □ Children (average age 3-16 years)  □ **Other (EXCLUDE)**  8. Sample clinical status:  □ FASD  □ FAS  □ pFAS  □ ARND  □ At risk of FASD  □ FAS based on facial dysmorphology alone  □ Confirmed or suspected prenatal alcohol exposure  □ Other PAE diagnosis (describe)________________  □ **None (EXCLUDE)** |
| 9. Intervention structure:  □ Structured  □ **Unstructured (EXCLUDE)**  10. Intervention delivery:  □ Face-to-face  □ Computerised  □ Both  □ **Neither/none (EXCLUDE)**  11. Intervention administered to:  □ Only child  □ Children and caregivers  □ Families  □ **None (EXCLUDE)** |
| 12. Reported outcomes (at least one measure of EF)  □ Cognitive flexibility  □ Working memory  □ Inhibition  □ Attention  □ Planning  □ Reasoning  □ Problem-solving  □ Other EF (describe) ____________________  □ **None (EXCLUDE)**  13. Study design:  □ RCT  □ Quasi-experimental  □ Single group pre-post  □ Systematic review and/or meta-analysis  □ Cluster-randomised trial  □ **None (EXCLUDE)** |
| 14. Is this study eligible for the review?  □ No, why?_________________________________  □ Yes  □ Need more information |
| 15. Comments |

## 3 Coding form

| **Interventions for executive functions in FASD review coding form** |
| --- |

**Section A: Document description**

1. Document ID#: __ __ __

2. Document type

□ 1. Journal article

□ 2. Thesis

□ 3. Unpublished source

□ 4. Book/chapter

□ 5. Conference

□ 6. Other (Specify):_____________________

3. Country

□ 1. Australia

□ 2. USA

□ 3. Canada

□ 4. Europe

□ 5. Asia

□ 6. Africa

□ 7. Other:_________________________

4. Year of publication? (text box)

**Section B: Methodological issues**

1. Study design?

□ 1. RCT

□ 2. Quasi-experimental

□ 3. Cluster-randomised

□ 4. Single group pre-post

□ 5. Other (specify) ______________________

2. What type of comparison condition was used?

□ 1. Treatment as usual (specify) ______________

□ 2. Waitlist control

□ 3. Comparison condition (specify)________________

□ 4. No treatment

□ 5. Alternative treatment (specify)________________

□ 6. Other (specify) ________________

3. How were groups formed?

□ 1. Pre-existing groups

□ 2. Random allocation

□ 3. Matching (specify variables)____________

□ 4. Other (specify)____________

□ 5. Unclear

4. What was the unit of allocation?

□ 1. Dyads

□ 2. Participant

□ 3. Family

□ 4. Service site

□ 5. Other (specify)_______________

□ 6. Unclear

5. How were participants randomised? (specify)

6. Was randomisation consistent across sites?

□ 1. Yes

□ 2. No

□ 3. Unclear

□ 4. Other ________________

7. Were assessments of pre-intervention differences between groups assessed?

□ 1. Yes

□ 2. No

□ 3. Unclear

□ 4. Other (specify) ________________

8. Were there significant pre-existing differences pre-intervention?

□ 1. Yes

□ 2. No

□ 3. Unclear

□ 4. Other (specify) ________________

**Section C: Participants**

1. Who participated?

□ 1. Only child

□ 2. Child and mother

□ 3. Family (at least child, mother and father)

□ 4. Child and other caregiver (foster/grandparent)

□ 5. Child and father

□ 6. Other (specify) ________________

2. Reported clinical status of the child?

□ 1. FASD

□ 2. FAS

□ 3. pFAS

□ 4. ARND

□ 5. At risk of FASD

□ 6. Facial dysmorphology alone

□ 7. Confirmed PAE

□ 8. Suspected PAE

□ 9. Other (specify) ________________

3. Which diagnostic system was used (if any)?

□ 1. Institute of Medicine/Hoyme revision

□ 2. 4 Digit Code/Washington method

□ 3. Canadian Guidelines

□ 4. Australian Guidelines

□ 5. Other (specify)____________________

□ 6. Unclear

4. Other types of prenatal substance exposure?

□ 1. Yes (question 4)

□ 2. No

□ 3. Unclear

□ 4. Other (specify) ________________

5. Type of other prenatal substance exposure

□ 1. Tobacco

□ 2. Marijuana

□ 3. Amphetamines

□ 4. Benzodiazepines

□ 5. Marijuana

□ 6. Opioids

□ 7. Other psychoactive substances (specify) ________________

□ 8. Other (specify) ________________

6. How were participants recruited? (Text box)

7. Was attrition a cause for concern?

□ 1. Yes (specify) ________________

□ 2. No

□ 3. Unclear (specify) ________________

8. Sample characteristics

| Characteristics | Treatment | Comparison | Total |
| --- | --- | --- | --- |
| Age (M; SD; range) |  |  |  |
| Child Gender (*N* male; female) |  |  |  |
| Primary diagnosis |  |  |  |
| Comorbidities |  |  |  |
| Ethnicity |  |  |  |
| SES |  |  |  |
| Alcohol only |  |  |  |
| Alcohol plus Opioids only |  |  |  |
| Alcohol plus psychomotor stimulants (cocaine amphet) |  |  |  |
| Alcohol plus both opioids and psychomotor stimulants |  |  |  |
| Alcohol plus opioids and psychomotor stimulants and other |  |  |  |
| Alcohol plus other |  |  |  |

9. Number of participants

| *N* | Treatment | Comparison | Total |
| --- | --- | --- | --- |
| Referred |  |  |  |
| Consented |  |  |  |
| Assigned |  |  |  |
| Began intervention |  |  |  |
| Completed intervention |  |  |  |
| Follow-up 1 |  |  |  |
| Follow-up 2 |  |  |  |
| Follow-up 3 |  |  |  |

10. Any other comments on sampling? (Text box)

**Section D: Intervention**

1. To whom was the intervention administered?

□ 1. Children only

□ 2. Children and one caregiver

□ 3. Children plus more than one caregiver

□ 4. Unclear

□ 5. Other (specify)________________

2. Was the intervention individual or group format?

□ 1. Individual

□ 2. Group

□ 3. Both

□ 4. Other (specify)________________

3. How was the intervention administered?

□ 1. Face-to-face

□ 2. Computerised

□ 3. Both (F2F and Computerised)

□ 4. Other (specify)________________

4. What was the name of the intervention? (text box)

5. What was the setting for the intervention?

□ 1. health clinic

□ 2. Home

□ 3. School

□ 4. Other (specify)_______________

6. In what year was the intervention conducted? (text box)

7. Describe the intervention (text box)

8. Duration of intervention (minimum, maximum, average if known)? (text box)

9. Intensity of intervention (frequency of contact and length of contact)? (text box)

10. Who implemented the intervention?

□ 1. Psychologist/psychiatrist

□ 2. Occupational therapist

□ 3. Medical practitioner

□ 4. Other allied health practitioner

□ 5. Academic/researcher

□ 6. Other (specify)________________

□ 7. Unclear

11. Was there multiple intervention sites?

□ 1. Yes

□ 2. No

□ 3. Unclear

□ 4. Other (specify)________________

12. Was the intervention delivered as expected (ie were there any concerns around fidelity?

□ 1. Yes

□ 2. No

□ 3. Unclear

□ 4. Other (specify)________________

**Section E: Outcome measures (Collect for each outcome measure)**

1. What are the outcomes being measured?

□ 1. Inhibition

□ 2. Working memory

□ 3. Cognitive flexibility/shifting

□ 4. Planning

□ 5. Reasoning

□ 6. Attention

□ 7. Problem-solving

□ 8. Other (specify)________________

2. How was the outcome measured?

□ 1. Individual testing using behavioural/neuropsychological measures (e.g. Flanker task, DCCS)

□ 2. Observation (e.g. BRIEF, CBCL)

□ 3. Standardised assessment

□ 4. Self/other report

□ 5. Unclear

□ 6. Other (specify)________________

3. What was the name of the measure used? (text box)

4. Who completed the measure?

□ 1. Child

□ 2. Caregiver

□ 3. Teacher

□ 4. Practitioner

□ 5. Other (specify)________________

5. What time-point was the outcome measured?

□ 1. Baseline

□ 2. Post-baseline, pre-intervention

□ 3. Post intervention

□ 4. Short follow-up (0-3 months)

□ 5. Medium follow-up (>3 months – 6 months)

□ 6. Long-term follow-up (>6 months)

□ 7. Other (specify)________________

6. Was this outcome collected in the same manner across different groups?

□ 1. Yes

□ 2. No

□ 3. Unclear

□ 4. Other (specify)________________

7. Which condition recorded the more favourable outcome (ignoring significance)?

□ 1. Experimental

□ 2. Comparison

□ 3. No difference

□ 4. Unclear

□ 5. Other (specify)________________

8. If relevant, which direction did the outcome change from pre-post intervention (in experimental group)?

□ 1. Improved

□ 2. Diminished

□ 3. No change

□ 4. Unclear

□ 5. Other (specify)________________

9. Were differences for this outcome statistically significant?

□ 1. Yes

□ 2. No

□ 3. Not tested

□ 4. Unclear

□ 5. Other (specify)________________

10. Author’s conclusions about the outcome? (text box)

**Section F: Effect size data (collect for each outcome measure)**

1. Page number the effect size is reported on? (text box)

2. Type of effect size?

□ 1. Baseline difference between groups

□ 2. Post-intervention between groups

□ 3. Short-term follow-up between groups

□ 4. Medium follow-up between groups

□ 5. Long-term follow-up between groups

□ 6. Pre-post difference (single group)

□ 7. Pre-short term follow-up (single group)

□ 8. Pre-medium follow-up (single group)

□ 9. Pre-long-term follow-up (single group)

□ 10. Other (specify)________________

3. How was the effect size obtained?

□ 1. Reported in document

□ 2. Calculated

□ 3. Other (specify)________________

4. Effect size. (text box)
